# Impact of Traditional and Alternative Nicotine-Containing Product Use on Non-Surgical Periodontal Therapy: A Systematic Review and Network Meta-Analysis

**DOI:** 10.3390/dj14100616

**Published:** 2026-09-22

**Authors:** Orsolya Vámos, Péter Komora, Noémi Gede, Kata Kelemen, Márta Losonczi, Alexander Schulze Wenning, Gábor Varga, Péter Hegyi, Barbara Kispélyi

**Affiliations:** 1 Department of Prosthodontics, Semmelweis University, 1088 Budapest, Hungary; 2 Centre for Translational Medicine, Semmelweis University, 1085 Budapest, Hungary; 3 Department of Restorative Dentistry and Endodontics, Semmelweis University, 1088 Budapest, Hungary; 4 Institute for Translational Medicine, Medical School, University of Pécs, 7624 Pécs, Hungary; 5 Faculty of Dentistry, Semmelweis University, 1088 Budapest, Hungary; 6 Department of Oral Biology, Semmelweis University, 1089 Budapest, Hungary; 7 Institute of Pancreatic Diseases, Semmelweis University, 1083 Budapest, Hungary

**Keywords:** periodontitis, subgingival instrumentation, smoking, e-cigarette, smokeless tobacco

## Abstract

**Background/Objectives**: Alternative nicotine-containing products have gained popularity in recent years. Our aim is to compare clinical periodontal outcomes following non-surgical periodontal therapy (NSPT) among patients with periodontitis according to nicotine exposure status: cigarette smokers (CS), former smokers (FS), quitters (Q), users of electronic cigarettes (EC), smokeless tobacco users (ST), and non-smokers (NS). **Methods**: A systematic search of four electronic databases (PubMed, EMBASE, Web of Science, CENTRAL) was performed until January 2025. Sixty-seven studies were included in the qualitative synthesis, of which 56 studies were included in a frequentist network meta-analysis. Two researchers independently collected data on probing pocket depth (PPD), clinical attachment loss, bleeding on probing, plaque index, full mouth plaque score, gingival index, and gingival crevicular fluid volume. **Results**: NS had significantly higher change in PPD compared with CS (MD: 0.25; CI: 0.13, 0.37; 50 studies; 1359 patients; moderate certainty), EC (MD: 0.98; CI: 0.47, 1.49; 2 studies; 56 patients; very low certainty), and ST (MD: 0.99; CI: 0.49, 1.49; 2 studies; 82 patients; low certainty). Q (MD: 0.02; CI: −0.56, 0.60; 2 studies; 52 patients; low certainty) and FS (MD: 0.03; CI: –0.62, 0.69; 4 studies; 267 patients; low certainty) did not differ significantly from NS. In secondary outcomes, NS, FS, or Q were ranked first. **Conclusions**: FS and Q showed no statistically significant differences from NS; EC and ST generally had less favorable treatment rankings than CS. The certainty of evidence varied across exposure groups and outcomes. Further studies are needed to support firm clinical recommendations.

## 1. Introduction

Smoking is widely recognized as an environmental risk factor for periodontal disease, increasing the risk of both its development and progression [1,2]. Smokers have an approximately 85% (risk ratio: 1.85, 95% confidence interval: 1.5–2.2) higher likelihood of developing periodontal disease compared to non-smokers [2]. Clinically, this manifests as accelerated periodontal destruction, characterized by greater probing depth, increased clinical attachment loss, and more pronounced alveolar bone resorption, which can result in tooth loss [3].

Tobacco smoke impairs the host’s protective response to dental plaque biofilm, while increasing the production of destructive inflammatory cytokines and enzymes [4,5]. Smoking may alter the composition of the subgingival biofilm, promoting a higher prevalence of periodontal pathogenic species [5]. Additionally, tobacco use may hinder both gingival vascular responses through suppressed angiogenesis and changes in the vascular dynamics and cellular metabolism, and reduce the reparative capacity of periodontal cells [6].

While global smoking rates are decreasing [7], alternative nicotine-containing products (e.g., electronic cigarettes, heated tobacco products, smokeless tobacco) have gained popularity in recent years, especially among younger populations [8]. Alternative nicotine-containing products are usually considered to be a relatively safer option than conventional cigarettes due to the absence of combustion and fewer toxicants [9,10]. E-cigarettes have proven to be more effective in smoking cessation compared to conventional nicotine-replacement therapy [11], although recently there has been growing concern over their safety for periodontal health [12].

Subgingival instrumentation, also known as non-surgical periodontal therapy (NSPT), utilizes hand instruments, including ultrasonic scalers and curettes, and it is frequently used for the management of periodontal diseases [13]. It is recognized that the results of periodontal therapy are compromised in cigarette smokers when compared with those who do not consume any form of tobacco [14,15].

Chang et al. [14] compared the effect of NSPT in traditional cigarette smokers and non-smokers in a recent meta-analysis and found that smoking has a significant adverse effect on clinical outcomes of NSPT. Heasman et al. [16] reviewed the evidence for the relative clinical responses to periodontal treatment in cigarette smokers, non-smokers and ex-smokers and concluded that quitting smoking is likely to be beneficial to periodontal health. Shah et al. [17] reported a less favorable response to periodontal treatment among e-cigarette users compared with non-smokers, while Charde et al. [18] summarized the available evidence on the periodontal effects of e-cigarette use, including treatment outcomes. However, no comprehensive analysis has compared conventional and alternative nicotine-containing products, as well as smoking cessation, following NSPT.

Therefore, this systematic review and network meta-analysis aimed to address the following PECOS question: Among patients with periodontitis undergoing non-surgical periodontal therapy, how does the use of different nicotine-containing products (cigarette smoking, electronic cigarette use, smokeless tobacco use), as well as smoking cessation (former smokers and quitters), compare with non-smoking in terms of changes in clinical periodontal outcomes including probing pocket depth and other periodontal outcomes based on evidence from two- or multi-armed randomized and non-randomized interventional studies? We hypothesized that NSPT is more effective in non-smokers than in smokers of any nicotine-containing product, with ex-smokers and quitters exhibiting a response that is between the two groups.

## 2. Materials and Methods

### 2.1. Protocol and Registration

Our systematic review and meta-analysis are reported in accordance with the recommendations of the PRISMA 2020 guideline [19] (Supplementary Table S4), and the Cochrane Handbook [20] has been followed. The study protocol was registered on the PROSPERO International Prospective Register of Systematic Reviews (registration number CRD42025635881). This work was carried out as part of the Systems Education Program at Semmelweis University [21,22].

### 2.2. Eligibility Criteria

#### 2.2.1. Inclusion Criteria

Studies assessing the effect of non-surgical periodontal therapy in various nicotine-containing product users and non-smokers were considered eligible for our systematic review and meta-analysis. We defined a question and created a PECOS (patient/population-exposure-control-outcome) study design framework:

- Population (P): patients with periodontitis undergoing non-surgical periodontal therapy
- Exposure (E): different types of nicotine-containing products:
  E1: traditional cigarette smokers (CS): exclusive users, dual users not included
  E2: electronic cigarette users (EC): exclusive users, dual users not included
  E3: former smokers (FS): traditional cigarette smokers who stopped smoking at least 6 months prior to the baseline examination
  E4: quitters (Q): traditional cigarette smokers who were willing to quit after baseline examination
  E5: smokeless tobacco users (ST): exclusive users, dual users not included
- Control (C): non-smokers (non-users of any nicotine and tobacco product)
- Outcome (O):
  Primary outcome: Probing Pocket Depth (PPD) measured in millimeters;
  Secondary outcomes: Clinical Attachment Loss (CAL), Bleeding on Probing (BOP), Plaque Index (PI), Full mouth Plaque score (FMPS), Gingival Index (GI), gingival crevicular fluid (GCF) volume.
- Study design (S): two- or multi-armed randomized and non-randomized interventional studies

#### 2.2.2. Exclusion Criteria

We excluded case reports, case series, animal studies, in vitro research, review articles, abstracts, posters, letters, and editorials. Studies of patients with self-reported systemic diseases that can influence periodontal health, e.g., uncontrolled diabetes mellitus, and individuals taking medications that could contribute to drug-induced gingival overgrowth (cyclosporin, amlodipine, or phenytoin) were excluded. Studies including patients diagnosed with aggressive periodontitis, as defined by earlier classification systems [23], were excluded. We excluded studies with follow-up periods of less than one month or more than one year.

### 2.3. Information Sources and Search Strategy

A systematic search of four electronic databases (PubMed (MEDLINE), EMBASE, Web of Science, and Cochrane Library (Cochrane Central Register of Controlled Trials) (CENTRAL) was performed in January 2025. Our search strategy corresponded to the PECOS framework. Also, the reference lists of eligible publications and review articles were evaluated. A detailed search key can be found in Supplementary Document S1.

### 2.4. Selection Process

After conducting a systematic search, the references were imported into a citation management program (Rayyan; https://www.rayyan.ai/, accessed on 12 January 2025) [24] and screened for duplicates, which were deleted both automatically and manually. Following a calibration exercise to harmonize the screening approach, two review authors (VO and KP) independently examined titles, abstracts, and full texts against specified eligibility criteria. Throughout the selection procedure, Cohen’s kappa coefficient [25] was calculated to determine inter-rater reliability. A third reviewer (KK) resolved the disagreements.

### 2.5. Data Collection Process and Data Items

Using a standardized data collection form, two separate reviewers (VO and KP) extracted data, and a third independent reviewer (KK) settled any discrepancies. The following data were extracted from each eligible article: first author, year of publication, country, digital object identifier, study design, study period, patient demographics, type of nicotine-containing product used, duration and frequency of smoking, follow-up period, and mean and standard deviation of the outcomes. Additionally, information on periodontal status, periodontal treatment, and examined sites were collected. Study authors were contacted in case of missing or unclear information.

### 2.6. Study Risk of Bias Assessment

The ROBINS-E tool [26] was used for assessing risk of bias in non-randomized studies; the Risk of Bias 2 (RoB 2) tool [27] was used to assess the risk of bias in randomized controlled trials. Two independent review authors (VO and KP) evaluated the included records separately, and any disagreements between them were discussed until a consensus was reached. A third review author (KK) was consulted to resolve conflicts.

### 2.7. Synthesis Methods and Effect Measures

The mean difference (MD) for continuous data using a frequentist method was used to perform pairwise meta-analyses and network meta-analyses (NMAs) applying a random-effects model with 95% confidence intervals (95% CI). Subgroup analyses were based on the examined sites. Sensitivity analyses were conducted excluding high-risk studies and including only randomized clinical trials. Forest plots were used to examine consistency, representing the relationship among direct, indirect, and network point estimates. An evidence plot shows the direct and indirect estimation ratio in network meta-analysis. Funnel plots were created for each outcome (PPD, CAL, BOP, PI, FMPS, GI, and GCF volume), and Egger’s tests were performed to assess small-study effects. P-scores rank treatments, which measure the certainty that one treatment is better than another, averaged over all competing treatments. Calculations were performed using the R package (version 4.1.1), Netmeta (version 2.7-0) and Pairwise (version 0.6.0-0).

### 2.8. Reporting Bias Assessment

Potential reporting bias and small-study effects were assessed using funnel plots and Egger’s regression test for each network meta-analysis where sufficient studies were available. Funnel plot asymmetry was visually inspected, and Egger’s test was used to statistically assess potential small-study effects. A *p*-value < 0.05 was considered indicative of statistically significant asymmetry.

### 2.9. Certainty Assessment

The Confidence in Network Meta-Analysis (CINeMA) [28] web application was used to determine the certainty of the evidence. The following factors were assessed: within-study bias, reporting bias, indirectness, imprecision, heterogeneity, and incoherence.

## 3. Results

### 3.1. Search and Selection

After the selection process (Figure 1), a total of 67 studies (Supplementary Table S1) were included in the qualitative synthesis [17,29,30,31,32,33,34,35,36,37,38,39,40,41,42,43,44,45,46,47,48,49,50,51,52,53,54,55,56,57,58,59,60,61,62,63,64,65,66,67,68,69,70,71,72,73,74,75,76,77,78,79,80,81,82,83,84,85,86,87,88,89,90,91,92,93,94]. A comprehensive list of studies excluded at the full-text screening stage, along with the specific reasons for exclusion, is provided in Supplementary Table S2. Out of the 67 included studies, 56 were suitable for inclusion in the quantitative synthesis [17,29,32,33,34,35,36,37,39,40,43,44,45,46,47,49,50,51,52,53,54,55,56,58,59,60,61,62,63,64,65,66,67,68,70,72,73,74,75,77,78,79,80,81,82,83,84,85,86,87,88,89,90,91,93,94], as only studies reporting data as mean ± standard deviation or mean ± standard error were eligible for analysis. The reference lists of the included articles were analyzed using a citation chaser program. Following a comprehensive process of backward and forward chasing, two additional eligible articles were included in our analysis.

### 3.2. Basic Characteristics of the Included Studies

All included studies were published in English between 1985 and 2024. Randomized clinical trials and non-randomized interventional studies were included in our analysis [17,29,30,31,32,33,34,35,36,37,38,39,40,41,42,43,44,45,46,47,48,49,50,51,52,53,54,55,56,57,58,59,60,61,62,63,64,65,66,67,68,69,70,71,72,73,74,75,76,77,78,79,80,81,82,83,84,85,86,87,88,89,90,91,92,93,94]. All participants were diagnosed with periodontitis; however, the extent and severity of the disease varied across studies. According to the 2017 classification of periodontal diseases, the study populations had at least localized or generalized Stage II periodontitis, with Grade A in non-smokers and Grade B in smokers, although this classification was not explicitly reported in all included studies [95]. More detailed information regarding the characteristics of the included studies—such as study design, country, number of participants, mean age, smoking status, periodontal status, non-surgical treatment, examined sites, follow-up time, and reported outcomes—is presented in the basic characteristics table (Supplementary Table S1) [17,29,30,31,32,33,34,35,36,37,38,39,40,41,42,43,44,45,46,47,48,49,50,51,52,53,54,55,56,57,58,59,60,61,62,63,64,65,66,67,68,69,70,71,72,73,74,75,76,77,78,79,80,81,82,83,84,85,86,87,88,89,90,91,92,93,94]. A summary of the results of all included studies is presented in Supplementary Table S3 [17,29,30,31,32,33,34,35,36,37,38,39,40,41,42,43,44,45,46,47,48,49,50,51,52,53,54,55,56,57,58,59,60,61,62,63,64,65,66,67,68,69,70,71,72,73,74,75,76,77,78,79,80,81,82,83,84,85,86,87,88,89,90,91,92,93,94].

### 3.3. Risk of Bias

The results of study-level and domain-level risk of bias are shown in Supplementary Document S2. Most RCTs carried no risk, whereas most non-randomized studies raised some concerns, mainly because of bias arising from measurement of the exposure, selection of the participants, or due to confounding or missing outcome data.

### 3.4. Synthesis of the Results

Sixty-seven studies were included in the qualitative synthesis, of which 56 studies were included in a frequentist network meta-analysis. Network characteristic tables, including the most important data for each group, were used to describe the results of each analysis (Supplementary Document S3).

#### 3.4.1. Primary Outcome—Probing Pocket Depth (PPD)

The network (Figure 2A) includes 52 studies [17,29,32,33,34,35,36,37,39,40,43,44,45,46,47,49,50,53,54,55,56,58,59,60,61,62,63,64,65,66,67,68,70,73,74,75,77,78,79,80,81,82,83,84,85,86,87,88,89,90,91,93,94]. The total number of patients in the network is 3209. We ranked smoking and nicotine exposures using P-scores (Figure 2B), where higher values indicate a greater likelihood of being associated with the most favorable outcomes between baseline and re-evaluation. Under the random-effects model, NS (P-score = 0.8301) is likely to have the biggest change in PPD. Q (P-score = 0.7570), FS (P-score = 0.7431), CS (P-score = 0.4660), and EC (P-score = 0.1038) were ranked in that order. ST (P-score = 0.1001) had the least favorable treatment response. The league table (Figure 2C) presents both direct (upper right triangle) and network (lower left triangle) comparisons between smoking and nicotine exposures. Overall, NS had significantly higher change in PPD compared with CS (MD = 0.25, CI: 0.13, 0.37), EC (MD = 0.98, CI: 0.47, 1.49), and ST (MD = 0.99, CI: 0.49, 1.49). Q (MD = 0.02, CI: −0.56, 0.60) and FS (MD = 0.03, CI: –0.62, 0.69) did not differ significantly from NS.

#### 3.4.2. Secondary Outcomes

##### Clinical Attachment Loss (CAL)

The network (Figure 3A) includes 44 studies [17,29,32,33,34,35,36,37,39,40,43,46,47,49,50,53,55,56,58,59,60,61,62,63,64,66,67,68,75,77,79,80,81,82,83,84,86,87,88,89,90,93,94]. The total number of patients in the network is 2323. We ranked smoking and nicotine exposures using P-scores (Figure 3B), where higher values indicate a greater likelihood of being associated with the most favorable outcomes between baseline and re-evaluation. Under the random-effects model, NS (P-score = 0.8355) is likely to have the biggest change in CAL. Q (P-score = 0.6182), FS (P-score = 0.5843), CS (P-score = 0.4698), and ST (P-score = 0.2594) were ranked, respectively. EC (P-score = 0.2326) had the least favorable treatment response. The league table (Figure 3C) presents both direct (upper right triangle) and network (lower left triangle) comparisons between smoking and nicotine exposures. Overall, NS had significantly greater change in CAL compared with CS (MD = 0.13, CI: 0.02, 0.25). There were no significant differences between other exposure groups.

##### Bleeding on Probing (BOP)

The network (Figure 4A) includes 25 studies [17,33,34,35,36,37,49,50,54,56,60,62,66,67,70,72,76,77,78,79,81,82,86,90,91]. The total number of patients in the network is 1231. We ranked smoking and nicotine exposures using P-scores (Figure 4B), where higher values indicate a greater likelihood of being associated with the most favorable outcomes between baseline and re-evaluation. Under the random-effects model, FS (P-score = 0.8616) is likely to have the biggest change in BOP. NS (P-score = 0.7562), Q (P-score = 0.5664), CS (P-score = 0.3734), and EC (P-score = 0.3020) were ranked, respectively. ST (P-score = 0.1404) had the least favorable treatment response. The league table (Figure 4C) presents both direct (upper right triangle) and network (lower left triangle) comparisons between smoking and nicotine exposures. Overall, NS had significantly greater change in BOP compared with CS (MD = 10.48, CI: 3.90, 17.5). There were no significant differences between other exposure groups.

##### Other Clinical Parameters and GCF Volume

Results of the analysis of Plaque index, Gingival index, Full mouth plaque score, and GCF volume are presented in Supplemental Document S4. The most important network comparisons of all outcomes are summarized in the Summary of Findings table (Table 1).

#### 3.4.3. Subset and Sensitivity Analyses

Subset analysis based on the examined sites was conducted when sufficient data were available. Subset 0 included studies where full mouth indices were measured [17,29,32,33,34,35,36,37,39,44,46,49,50,51,52,53,54,55,56,58,59,60,61,62,64,65,66,67,68,72,73,74,75,78,79,81,82,86,87,88,89,90,91,94], while subset 1 included studies where only deep pockets (PPD ≥ 5 mm) were examined [36,40,43,45,47,49,53,59,63,66,70,72,74,75,77,78,79,83,84,85,91,93]. The subset analysis revealed no major differences in ranking probability. Subgroup division and analysis results are detailed in Supplementary Document S5. First sensitivity analysis (Supplementary Document S7) was conducted excluding high-risk studies [17,32,44,45,61,62]. All studies including EC users were assessed as high risk of bias; therefore, this group was excluded from the sensitivity analysis. For the remaining comparisons, the sensitivity analysis revealed no substantial changes in ranking probabilities. A second sensitivity analysis (Supplementary Document S8) was conducted including only randomized clinical trials [33,34,35,43,47,63,68,71,80,83,85,87,90]. As no RCTs included quitters or electronic cigarette users, these exposure groups could not be included in the sensitivity analysis. Gingival crevicular fluid (GCF) volume was assessed in only one study; therefore, a network meta-analysis could not be performed for this outcome. Furthermore, only one RCT included former smokers (FS) and only one included smokeless tobacco (ST) users, resulting in weak or poorly connected networks for these exposure categories. The Plaque index (PI) and gingival index (GI) analyses included only non-smokers (NS) and cigarette smokers (CS), as no RCTs were available for the other exposure groups. The sensitivity analysis resulted in some changes in the treatment rankings compared with the primary network meta-analysis. Notably, ST ranked higher than CS for CAL and BOP, while FS showed lower ranking positions for CAL. However, these changes should be interpreted with considerable caution, as only one randomized clinical trial contributed to the FS and ST groups.

### 3.5. Reporting Bias

Funnel plots were visually inspected for potential asymmetry, and Egger’s regression tests were performed to assess possible small-study effects. The results of these assessments are presented for each outcome where the number of included studies was sufficient to allow assessment (Supplementary Document S4).

### 3.6. Certainty of Evidence

Using the CINeMA tool, the quality of evidence was found to be moderate to very low. The confidence rating was at most moderate due to the inclusion of non-randomized studies. The evidence was also downgraded due to within-study bias, heterogeneity, and incoherence (Supplementary Document S6). The characteristics of studies contributing to direct comparisons were assessed to ensure sufficient clinical and methodological similarity for valid indirect comparisons. Minor incoherence was observed in a few direct versus indirect comparisons, but these represented only a small proportion of the network. The majority of comparisons were consistent, supporting the overall validity of the network estimates.

## 4. Discussion

To the best of our knowledge, this is the first comprehensive systematic review and network meta-analysis assessing the effects of conventional and alternative nicotine-containing products, as well as smoking cessation, on clinical outcomes after non-surgical periodontal therapy. The findings of the current investigation suggest that current nicotine-containing product users may respond less favorably to NSPT than non-smokers. However, the certainty of evidence varied across exposure categories, with moderate-certainty evidence for cigarette smokers, low-certainty evidence for smokeless tobacco users, and very low-certainty evidence for electronic cigarette users. Former smokers and quitters did not show significant differences from non-smokers in the assessed outcomes, although the certainty of evidence was low in these exposure groups as well. The results of the present study are consistent with previous narrative [16,96] and systematic reviews [14,97,98] and meta-analyses [14,97,98]; however, those studies did not consider alternative nicotine-containing products.

Our analysis revealed that NS achieved the most substantial improvements in periodontal parameters, including greater reductions in PPD and CAL gains. Interestingly, former smokers and quitters did not differ significantly from NS in any of the assessed outcomes, suggesting a potentially more favorable periodontal treatment response following smoking cessation. These findings may indicate that the detrimental effects of smoking on periodontal tissues may be, at least partially, reversible following cessation [99,100]. Several biological mechanisms have been proposed to underlie this potential improvement, including a shift toward a less pathogenic subgingival microbiota, improved gingival microcirculation, normalization of neutrophil activity and cellular metabolism, attenuation of the heightened immune response, and re-establishment of cytokine homeostasis both locally and systemically [16].

The effect of CS has not been entirely consistent across studies. While the majority of studies indicate that NS experience greater PPD reductions and CAL gains following therapy, several investigations have failed to detect significant differences between the two groups [50,55,86]. D’Aiuto et al. [44] proposed that local disease-related parameters—such as the extent of posterior tooth involvement, number of interproximal lesions, baseline mobility, and initial pocket depth—may exert a more substantial influence on clinical outcomes than smoking status alone. In the present NMA, CS exhibited statistically significantly smaller reductions in PPD and less CAL gain than NS. Nevertheless, the mean differences between groups (0.25 mm in PPD and 0.13 mm in CAL) were relatively small, indicating that smoking may be associated with a less favorable therapeutic response, although the magnitude of the observed difference was relatively small [14]. It is important to note that, while non-smokers demonstrated superior responses to periodontal therapy (ΔPPD = 1.38 mm), evidence suggests that smokers also benefit from treatment (ΔPPD = 0.99 mm). These findings indicate that smokers also benefit from NSPT, although the observed improvement was smaller than that seen in non-smokers.

Alternative nicotine-containing products are often perceived as safer than traditional cigarettes [9]. In our analysis, EC and ST users generally showed less favorable ranking positions than CS across the assessed outcomes. However, these findings should be interpreted with caution, as the networks for EC and ST were sparse and were based on only one or two studies for several outcomes, with low or very low certainty of evidence. Therefore, the observed ranking differences should be considered exploratory and are insufficient to establish whether these products provide any clinical advantage or disadvantage compared with conventional cigarettes. It has been shown that while periodontally healthy EC users, especially with good oral hygiene, may exhibit periodontal parameters comparable to those of non-smokers [101,102], this does not appear to be the case in patients with periodontitis, where EC use may be associated with a less favorable response to periodontal therapy. Sundar et al. [103] in an in vitro study found that flavoring agents used in electronic cigarettes can enhance oxidative stress and promote pro-inflammatory and pro-senescence responses—such as DNA damage and reduced histone deacetylase activity—which may contribute to impaired repair mechanisms in PDL cells. Findings from clinical studies suggest that vaping affects the periodontium through three main mechanisms: impaired immune and inflammatory responses, a microbial shift favouring periodontal pathogens, and reduced soft tissue healing capacity [18,32,104,105]. In the case of smokeless tobacco, in vitro studies [106] found that smokeless tobacco extract directly inhibited the normal function of periodontal ligament and gingival fibroblasts, suggesting that these products may affect cellular mechanisms involved in periodontal repair. Payne et al. [107] reported that the use of smokeless tobacco products reduces macrophage volume and density and is associated with erythema, ulceration, and white striations in oral tissues [90].

In our study, NS had significantly greater improvement in BOP than CS, and greater change in GI than CS or EC. The vasoconstrictive effect of nicotine may reduce peripheral blood flow and thereby attenuate clinical signs such as bleeding and edema in smokers [108,109]. This potential masking effect may reduce the visibility of clinical signs of gingival inflammation, while potentially affecting the resolution of inflammation in the marginal tissues after therapy [14,97].

Clinical outcomes following NSPT are known to be influenced by patient-related factors, including the quality of oral care and compliance during treatment. In the present study, no significant differences in FMPS were observed among the groups; however, EC exhibited a significantly smaller improvement in PI compared to all other groups. It has been shown that EC users tend to have a lower plaque index than CS [101]; however, in the articles included in our analysis, EC had baseline plaque index values comparable to those of CS.

In our analysis, CS and EC had significantly smaller reductions in GCF volume than NS. Hao et al. [110] reported that following periodontal therapy, smokers with periodontitis exhibited significantly higher GCF IL-1β levels than non-smokers; however, no significant differences in baseline cytokine levels were observed between the two groups.

Sites with deeper PPD typically show greater reduction after SRP compared to shallow sites [111]. When sufficient data were available, a site-specific subgroup analysis (PPD ≥ 5 mm) was conducted, which revealed no substantial changes in the ranking probabilities. A dose–response relationship is often seen between the amount of cigarette use and the severity of its negative effects on periodontal health [112]. However, many included studies did not clearly report duration or daily frequency of smoking; therefore, we could not evaluate this impact. Only a few studies verified smoking status using biochemical markers such as cotinine, while most relied on self-reports. This represents an important source of exposure misclassification, as self-reported smoking status may not accurately reflect current nicotine exposure, intermittent or dual use, which could therefore have influenced the observed differences between groups. Most EC users are former traditional cigarette smokers who have quit smoking [113]; this was also the case in the studies included in our analysis. This factor may have influenced the results, as cumulative smoking exposure could have affected periodontal status and treatment response.

### 4.1. Strengths and Limitations

Regarding the strengths of the study, this was the first systematic review and network meta-analysis investigating the effects of traditional and alternative nicotine-containing products as well as smoking cessation on clinical outcomes after non-surgical periodontal treatment. Our study adhered to a preregistered protocol, employed rigorous methodology, and incorporated both univariate and multivariate analyses to ensure objectivity. Furthermore, a comprehensive assessment of multiple clinical outcomes was conducted, and we were able to establish direct comparisons among most of the products.

Some limitations of this meta-analysis should be acknowledged. For certain outcomes, only a limited number of studies were available, resulting in weak or incomplete networks. The network included both randomized and non-randomized studies, and RCTs were not available for quitters and electronic cigarette users, limiting the ability to draw causal inferences. The sensitivity analysis restricted to RCTs resulted in some changes in treatment rankings compared with the primary network meta-analysis, with ST users ranking higher than CS and FS ranking lower. However, these findings should be interpreted cautiously, as only one RCT contributed to the FS and ST groups, resulting in weak networks. The observed changes in ranking may therefore reflect the limited evidence base rather than true differences in treatment response. Furthermore, incomplete reporting and adjustment for potential confounders across the included studies may have resulted in residual confounding, meaning that observed differences between exposure groups may not be attributable solely to smoking or nicotine exposure status. Differences in baseline characteristics, previous smoking exposure, oral health behaviors, and other unmeasured factors may have influenced the findings. This limitation is particularly relevant when interpreting the lack of statistically significant differences between former smokers or quitters and non-smokers, as these findings should not be interpreted as evidence of equivalent treatment responses or a causal benefit of smoking cessation. Finally, the absence of biochemical validation in most studies represents an important source of exposure misclassification and further limits the certainty of the results.

### 4.2. Implication for Practice

The integration of innovative scientific discoveries is essential for community dentistry [114,115]. The findings of this review may provide useful context for healthcare professionals; however, clinical recommendations regarding alternative nicotine-containing products cannot be firmly established based on the currently available evidence. Although EC and ST users generally showed less favorable treatment rankings than CS, the evidence for these comparisons was sparse and of low or very low certainty. Therefore, these findings should be interpreted as exploratory. Smoking cessation was associated with treatment outcomes comparable to those observed in non-smokers, although the certainty of evidence was limited for several comparisons. These findings should not be interpreted as evidence of equivalent treatment responses or a causal benefit of smoking cessation, particularly given the limitations of the available evidence.

### 4.3. Implication for Research

Well-designed, controlled multi-armed prospective clinical studies and randomized clinical trials are needed for direct comparison of different products. Further research is required about electronic cigarettes and smokeless tobacco products. Moreover, future studies should also investigate newer nicotine-containing products, such as heated tobacco products and tobacco-free nicotine pouches, as no studies have yet evaluated their effects on periodontal treatment outcomes. It is important to increase the transparency of confounding factors in the context of oral care and local disease-related parameters. Furthermore, we suggest the use of objective indicators of smoking exposure, such as cotinine levels and exhaled carbon monoxide, rather than relying solely on patient self-reports.

## 5. Conclusions

Current nicotine-containing product users generally showed less favorable responses to non-surgical periodontal therapy than non-smokers. Former smokers and quitters did not differ significantly from NS, while EC and ST users generally showed less favorable treatment rankings than CS. However, these findings are based on sparse evidence, with low or very low certainty for several comparisons, and should therefore be interpreted cautiously. More studies are needed to establish firm clinical recommendations regarding the comparative periodontal effects of alternative nicotine-containing products.

## Figures and Tables

**Figure 1:**
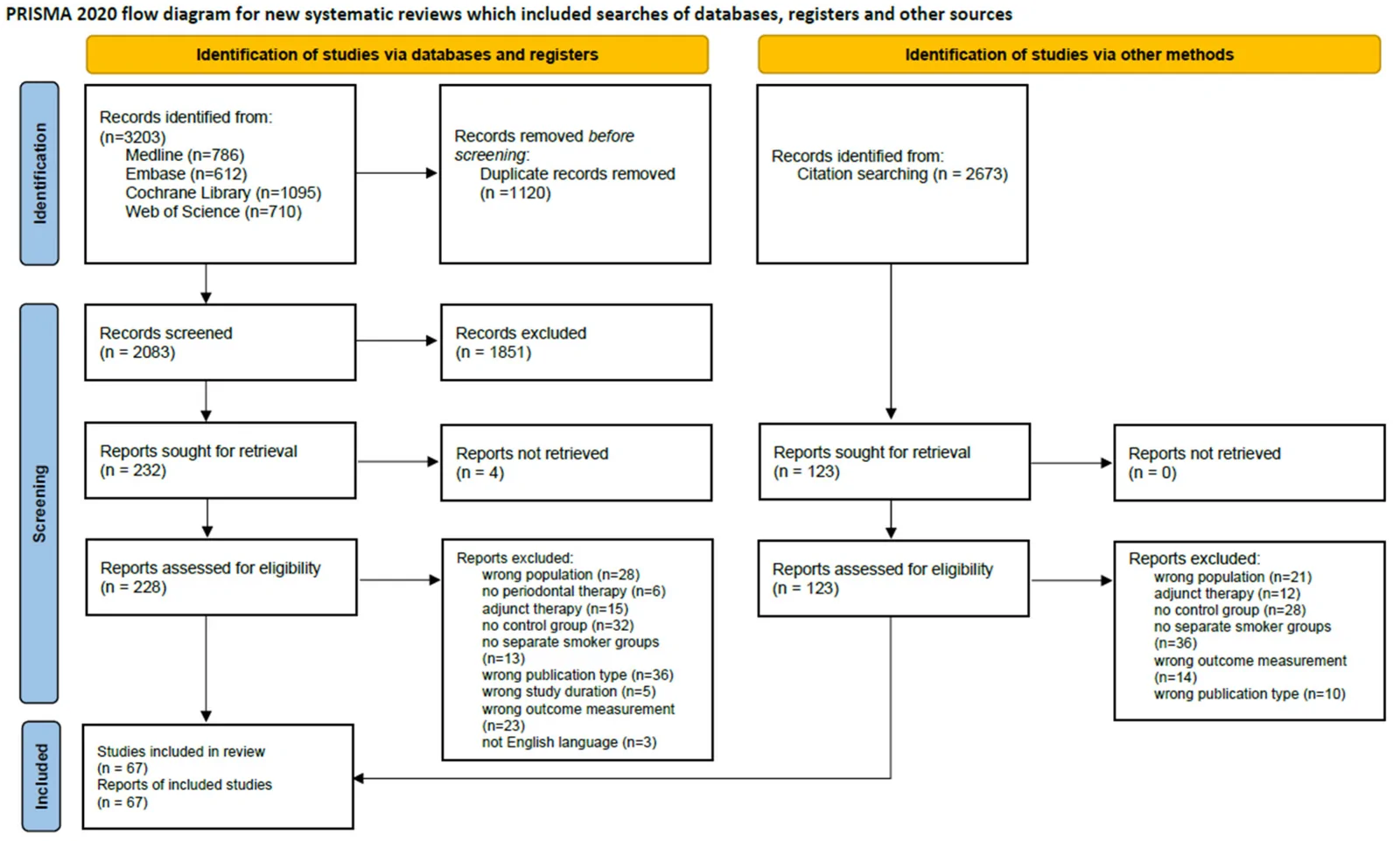
Study selection process, PRISMA flowchart [19] containing results of systematic search and article selection.

**Figure 2:**
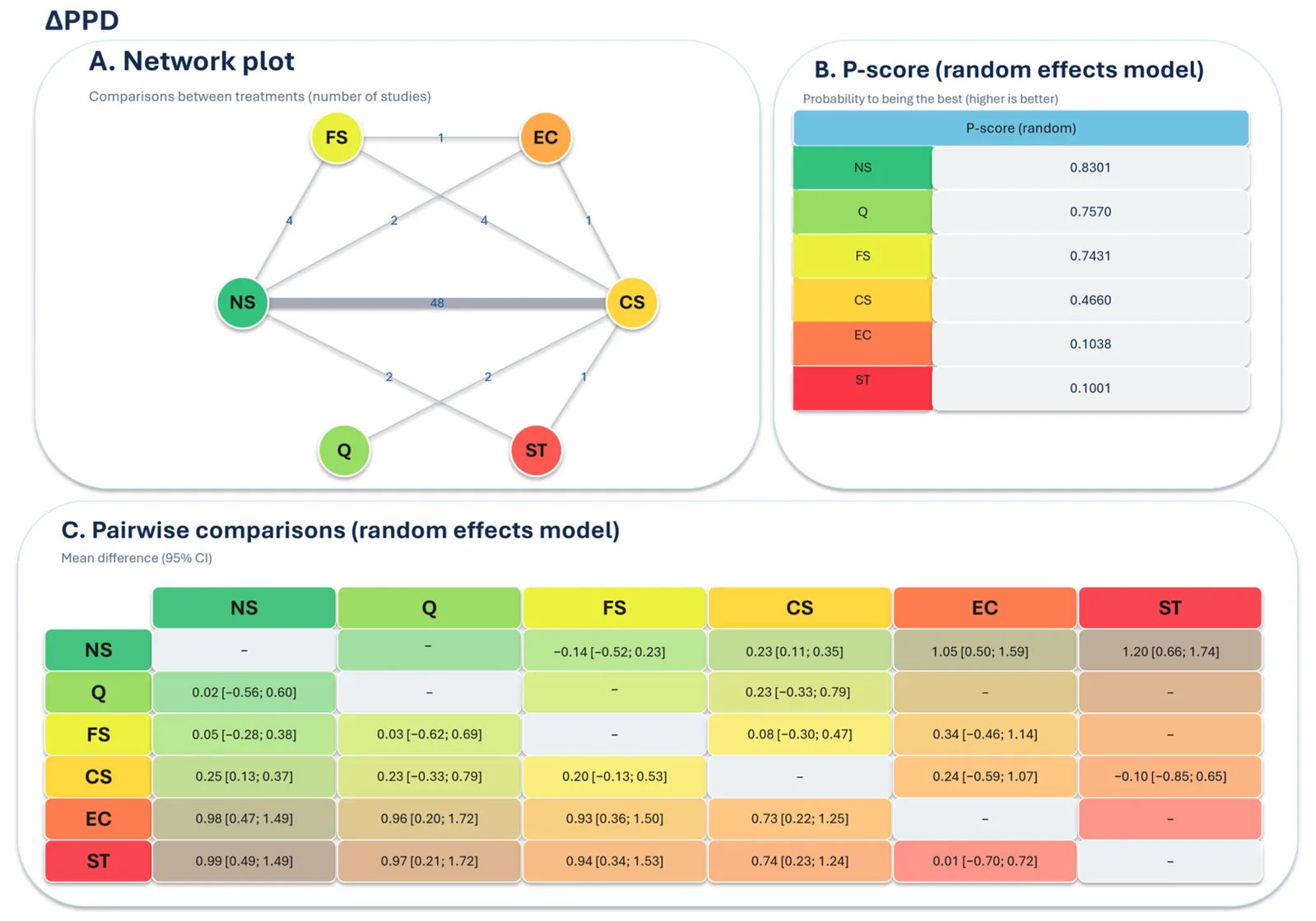
(**A**), Network geometry of the eligible comparisons of change in probing pocket depth (PPD) in different nicotine-containing product user groups and non-smokers; (**B**), Treatment ranking with P-scores under the random-effects model where higher values indicate larger outcome values, associated with favorable outcome between baseline and re-evaluation; (**C**), League table shows the mean difference and 95% confidence interval for direct (upper right triangle) and network (lower left triangle) comparisons; NS, non-smokers; Q, quitters; FS, former smokers; CS, cigarette smokers; EC, electronic cigarette; ST, smokeless tobacco.

**Figure 3:**
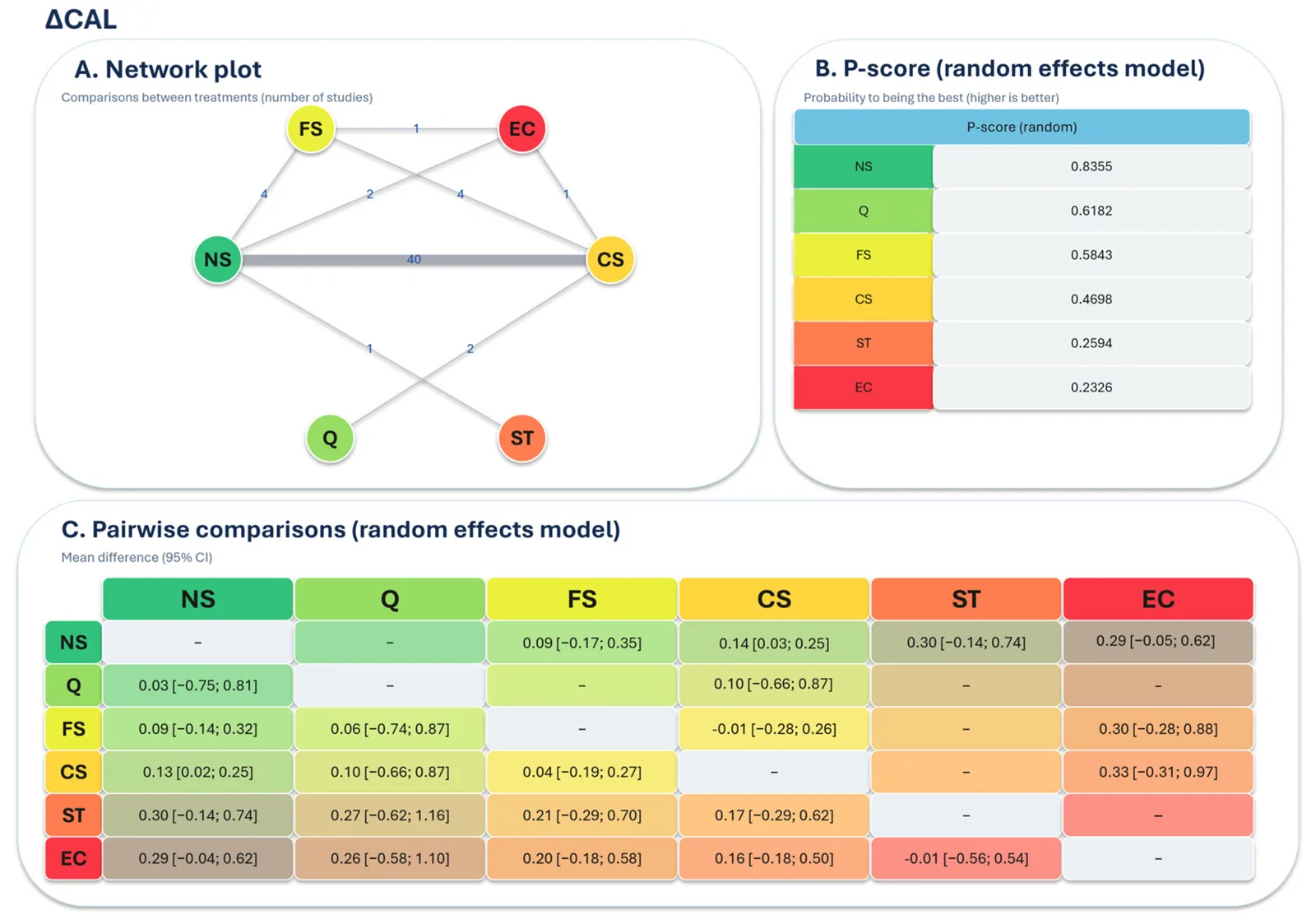
(**A**), Network geometry of the eligible comparisons of change in clinical attachment loss (CAL) in different nicotine-containing product user groups and non-smokers; (**B**), Treatment ranking with P-scores under the random-effects model, where higher values indicate larger outcome values, associated with favorable outcomes between baseline and re-evaluation. (**C**), League table shows the mean difference and 95% confidence interval for direct (upper right triangle) and network (lower left triangle) comparisons; NS, non-smokers; Q, quitters; FS, former smokers; CS, cigarette smokers; EC, electronic cigarette; ST, smokeless tobacco.

**Figure 4:**
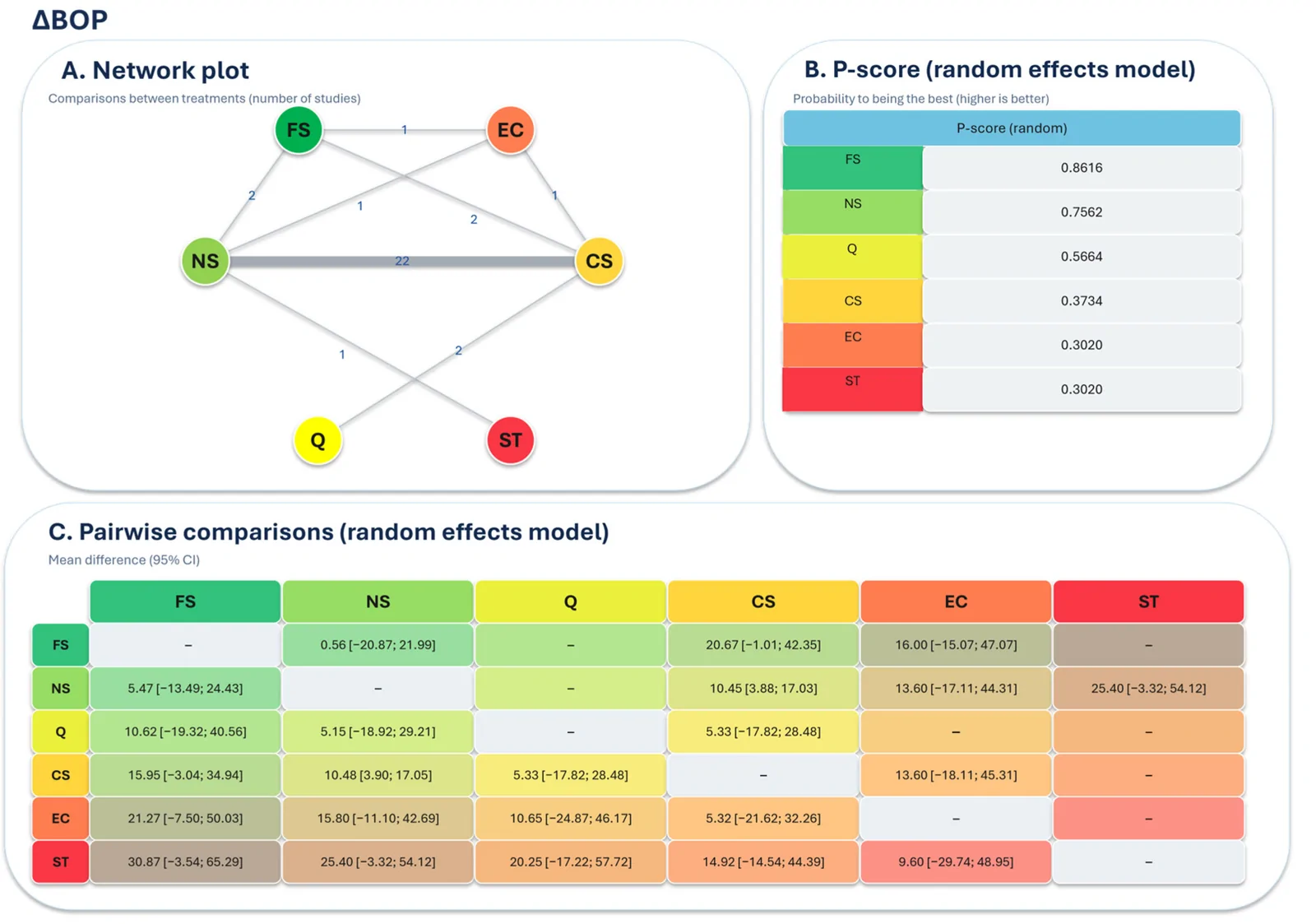
(**A**), Network geometry of the eligible comparisons of change in bleeding on probing (BOP) in different nicotine-containing product user groups and non-smokers; (**B**), Treatment ranking with P-scores under the random-effects model, where higher values indicate larger outcome values, associated with favorable outcomes between baseline and re-evaluation. (**C**), League table shows the mean difference and 95% confidence interval for direct (upper right triangle) and network (lower left triangle) comparisons; NS, non-smokers; Q, quitters; FS, former smokers; CS, cigarette smokers; EC, electronic cigarette; ST, smokeless tobacco.

**Table 1:** Summary of Findings table: Summary of the most important network comparisons for each outcome between baseline and re-evaluation following non-surgical periodontal therapy. PPD, probing pocket depth; CAL, clinical attachment loss; BOP, bleeding on probing; PI, plaque index; GI, gingival index; FMPS, full-mouth plaque score; GCF, gingival crevicular fluid; NS, non-smokers; CS, cigarette smokers; EC, electronic cigarette users; FS, former smokers; Q, quitters; ST, smokeless tobacco users; MD, mean difference; CI, confidence interval; mm, millimeter.

Outcome	Comparison	No. of Studies/ Direct Comparisons	Effect (MD, 95% CI)	Certainty (CINeMA)	Interpretation
ΔPPD measured in mm	NS vs. CS	48 (12 RCTs)	0.25 (0.13 to 0.37)	Moderate ^1^	Statistically significant greater PPD reduction in NS compared with CS, indicating a more favorable treatment response
	NS vs. EC	2	0.98 (0.47 to 1.49)	Very low ^1^	Significantly greater PPD reduction in NS, limited evidence
	NS vs. ST	2 (1 RCT)	0.99 (0.49 to 1.49)	Low ^1^	Significantly greater PPD reduction in NS compared with ST users
	NS vs. FS	4	0.05 (−0.28 to 0.38)	Low ^1^	No statistically significant difference between NS and FS, suggesting similar treatment response
	NS vs. Q	0	0.02 (−0.56 to 0.60)	Low ^2,3^	No statistically significant difference between NS and Q, no direct evidence, only network comparison
ΔCAL measured in mm	NS vs. CS	40 (11 RCTs)	0.13 (0.02 to 0.25)	Moderate ^1^	Statistically significant greater CAL gain in NS compared with CS
	NS vs. EC	2	0.29 (−0.04 to 0.62)	Very low ^1^	No statistically significant difference, limited evidence
	NS vs. ST	1 (1 RCT)	0.30 (−0.14 to 0.74)	Low ^1^	No statistically significant difference
	NS vs. FS	4	0.09 (−0.14 to 0.32)	Low ^1^	No statistically significant difference between NS and FS
	NS vs. Q	0	0.03 (−0.75 to 0.81)	Low ^2^	No statistically significant difference between NS and Q, no direct evidence, only network comparison
ΔBOP measured in %	NS vs. CS	22 (5 RCTs)	10.48 (3.90 to 17.5)	Low ^1,3^	Statistically significant greater reduction in BOP in NS compared with CS
	NS vs. EC	1	15.80 (−11.10 to 42.69)	Very low ^1^	No statistically significant difference; limited evidence
	NS vs. ST	1 (1 RCT)	25.40 (−3.32 to 54.12)	Low ^3^	No statistically significant difference
	NS vs. FS	2	5.47 (−13.49 to 24.43)	Low ^1^	No statistically significant difference between NS and FS
	NS vs. Q	0	5.15 (−18.92 to 29.21)	Low ^2,3^	No statistically significant difference between NS and Q, no direct evidence, only network comparison
ΔPI measured in a scale of 0–3	NS vs. CS	31 (5 RCTs)	0.06 (−0.05 to 0.17)	Moderate ^1^	No statistically significant difference in plaque index between NS and CS
	NS vs. EC	1	0.92 (0.37 to 1.47)	Very low ^1^	Significant difference: NS showed greater improvement; limited evidence
	NS vs. FS	1	0.24 (−0.26 to 0.74)	Low ^1^	No statistically significant difference
	NS vs. ST	1	0.09 (−0.41 to 0.59)	Low ^1^	No statistically significant difference
ΔGI measured in a scale of 0–3	NS vs. CS	25 (5 RCTs)	0.30 (0.17 to 0.42)	Low ^1^	Significant difference: NS showed greater improvement
	NS vs. EC	1	1.67 (1.12 to 2.22)	Very low ^1^	Significant difference: NS showed greater improvement
	NS vs. ST	1	0.43 (−0.09 to 0.94)	Low ^1^	No statistically significant difference
ΔFMPS measured in %	NS vs. CS	12 (4 RCTs)	2.90 (−3.36 to 9.16)	Low ^1^	No statistically significant difference
	NS vs. FS	2	1.73 (−11.35 to 14.81)	Low ^1^	No statistically significant difference
	NS vs. Q	0	5.24 (−14.49 to 24.98)	Low ^2^	No statistically significant difference, no direct evidence, only network comparison
	NS vs. EC	1	4.44 (−14.91 to 23.78)	Very low ^1^	No statistically significant difference; limited evidence
	NS vs. ST	1 (1 RCT)	12.00 (−6.94 to 30.94)	Moderate	No statistically significant difference
ΔGCF volume measured in μL	NS vs. CS	5 (1 RCT)	0.11 (0.01 to 0.21)	Low ^1^	NS demonstrated greater improvement, suggesting better inflammatory resolution
	NS vs. EC	1	0.57 (0.32 to 0.82)	Very low ^1,4^	NS demonstrated significantly greater improvement, limited evidence
	NS vs. ST	1	0.06 (−0.10 to 0.22)	Low ^1^	No statistically significant difference

^1^ Evidence downgraded due to within-study bias; ^2^ Evidence downgraded due to indirectness; ^3^ Evidence downgraded due to incoherence; ^4^ Evidence downgraded due to heterogeneity.

## Data Availability

The datasets used in this study can be found in full-text articles included in the systematic review and meta-analysis.

## Supplementary Materials

The following supporting information can be downloaded at: https://www.mdpi.com/article/10.3390/dj14100616/s1. Document S1: Search key; Document S2: Risk of bias assessment; Document S3: Network characteristics; Document S4: Additional figures for primary and secondary outcomes; Document S5: Results of the subgroup analysis; Document S6: Results of the CINeMA assessment; Document S7: Results of the sensitivity analysis excluding high risk studies; Document S8: Results of the sensitivity analysis including only RCTs; Table S1: Basic characteristics of the included studies; Table S2: List of excluded studies; Table S3: Summary of included studies; Table S4: PRISMA 2020 checklist.

## Abbreviations

The following abbreviations are used in this manuscript:

BOP	bleeding on probing
CAL	clinical attachment loss
CI	confidence interval
CS	cigarette smoker
EC	electronic cigarette
FMPS	full mouth plaque score
GCF	gingival crevicular fluid
GI	gingival index
HTP	heated tobacco product
NMA	network meta-analysis
MBL	marginal bone loss
MD	mean difference
NS	non-smoker
NSPT	non-surgical periodontal therapy
PECO	Population–Exposure–Control–Outcome framework
PI	plaque index
PPD	probing pocket depth
SD	standard deviation
ST	smokeless tobacco
