# Supplementary material for: Impact of Traditional and Alternative Nicotine-Containing Product Use on Non-Surgical Periodontal Therapy: A Systematic Review and Network Meta-Analysis
Source: Dent J (Basel). 2026 Sep 22;14(10):616. doi: 10.3390/dj14100616 (PMC13703454; doi:10.3390/dj14100616)
Supplement: Supplementary file 1 [file dentistry-14-00616-s001.zip › Document S1 (search key).pdf]

For each database, the following search query was employed:

EMBASE, COCHRANE:

(periodont*) AND ((non-surgical AND (therapy OR treatment)) OR NSPT OR scaling OR (root AND planing) OR SRP OR (mechanical AND debridement)) AND ((electronic AND cigarette) OR e-cigarette OR ENDS OR vaping OR nicotine OR cigarette OR smoking OR smoker OR tobacco)

PubMed:

(periodont*) AND ((non-surgical AND (therapy OR treatment)) OR NSPT OR scal* OR "root planing" OR SRP OR "mechanical debridement") AND ("electronic cigarette" OR "e-cigarette" OR ENDS OR "vaping" OR "nicotine" OR "cigarette" OR smok* OR "tobacco")

Web of Science:

(periodont*) AND ("non-surgical AND (therapy OR treatment)" OR "NSPT" OR scal* OR "root planing" OR "SRP" OR "mechanical debridement") AND ("electronic cigarette" OR "e-cigarette" OR "ENDS" OR "vaping" OR "nicotine" OR "cigarette" OR smok* OR "tobacco")

No filters were applied to any of the databases.
