# Supplementary material for: Impact of Traditional and Alternative Nicotine-Containing Product Use on Non-Surgical Periodontal Therapy: A Systematic Review and Network Meta-Analysis
Source: Dent J (Basel). 2026 Sep 22;14(10):616. doi: 10.3390/dj14100616 (PMC13703454; doi:10.3390/dj14100616)
Supplement: Supplementary file 1 [file dentistry-14-00616-s001.zip › Document S2 (ROB).pdf]

PPD

ROBINS-E:

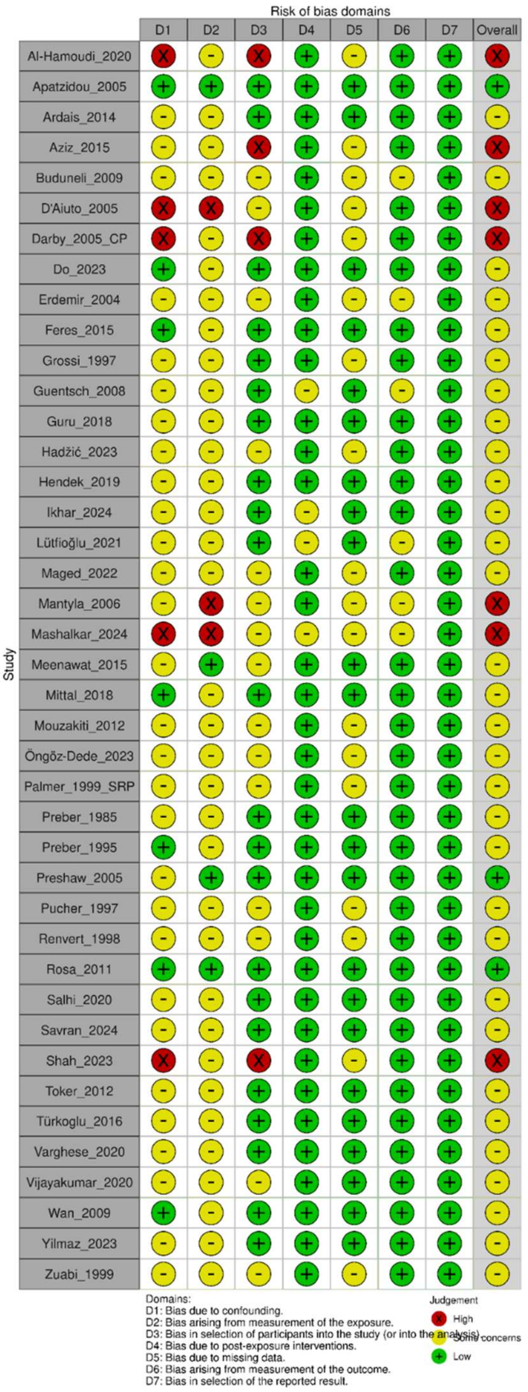

RoB 2.0:

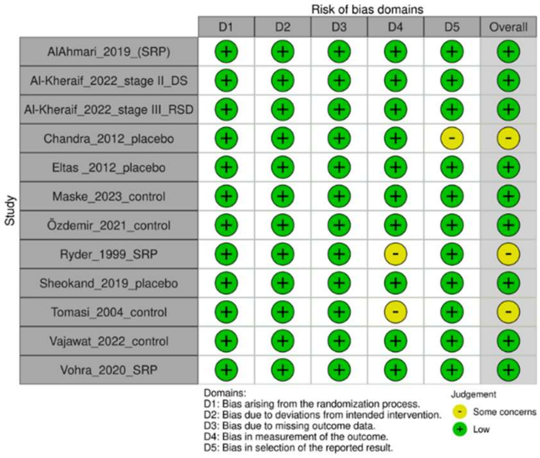

Figure S2.1. Risk of bias assessment of included articles for probing pocket depth (PPD). ROBINS-E tool was used for assessing risk of bias in non-randomized studies, RoB 2 tool was used to assess risk of bias in randomized controlled trials.

CAL

ROBINS-E:

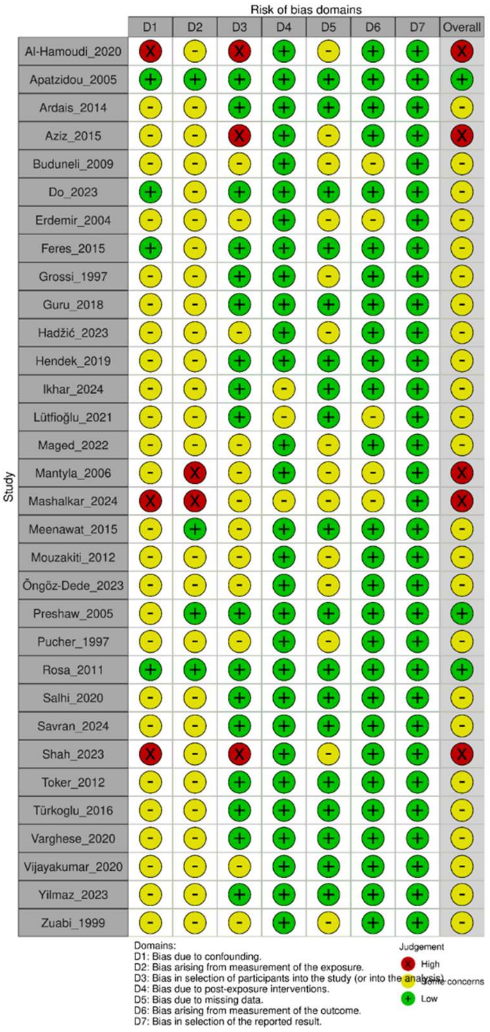

RoB 2.0:

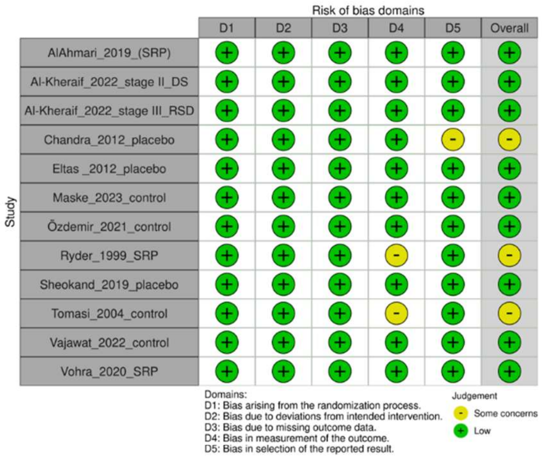

Figure S2.2. Risk of bias assessment of included articles for clinical attachment loss (CAL). ROBINS-E was used for assessing risk of bias in non-randomized studies, RoB 2 tool was used to assess risk of bias in randomized controlled trials.

BOP

ROBINS-E:

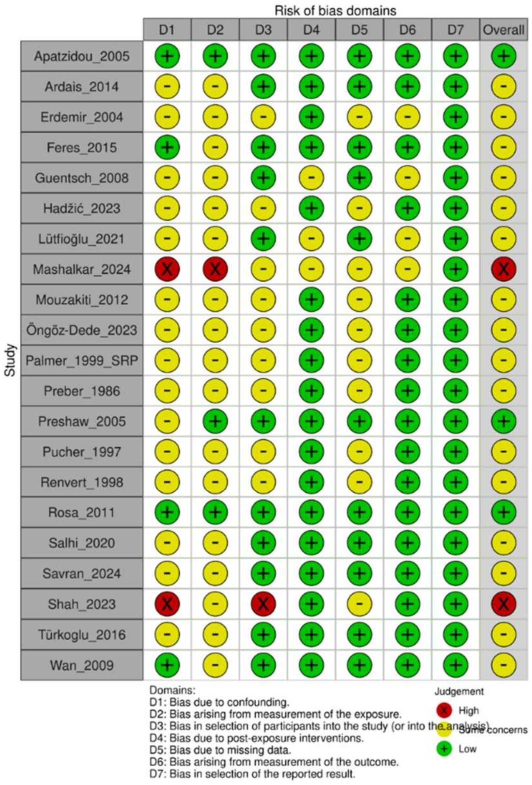

RoB 2.0:

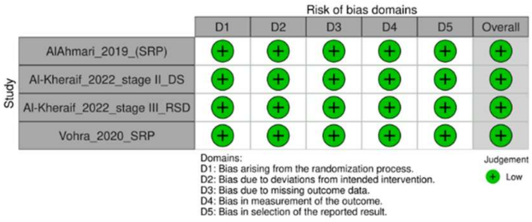

Figure S2.3. Risk of bias assessment of included articles for bleeding on probing (BOP). ROBINS-E was used for assessing risk of bias in non-randomized studies, RoB 2 tool was used to assess risk of bias in randomized controlled trials.

PI

ROBINS-E:

RoB 2.0:

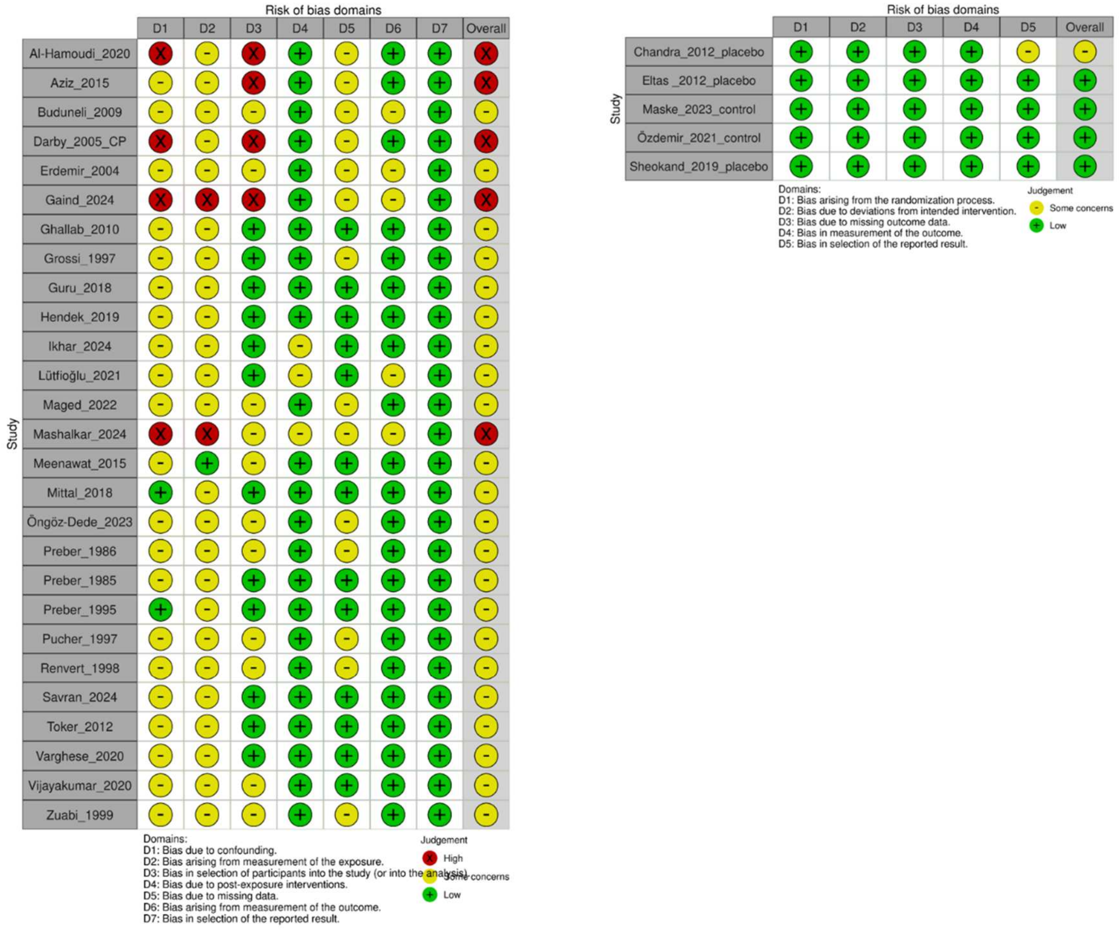

Figure S2.4. Risk of bias assessment of included articles for plaque index (PI). ROBINS-E was used for assessing risk of bias in non-randomized studies, RoB 2 tool was used to assess risk of bias in randomized controlled trials.

GI

ROBINS-E: RoB 2.0:

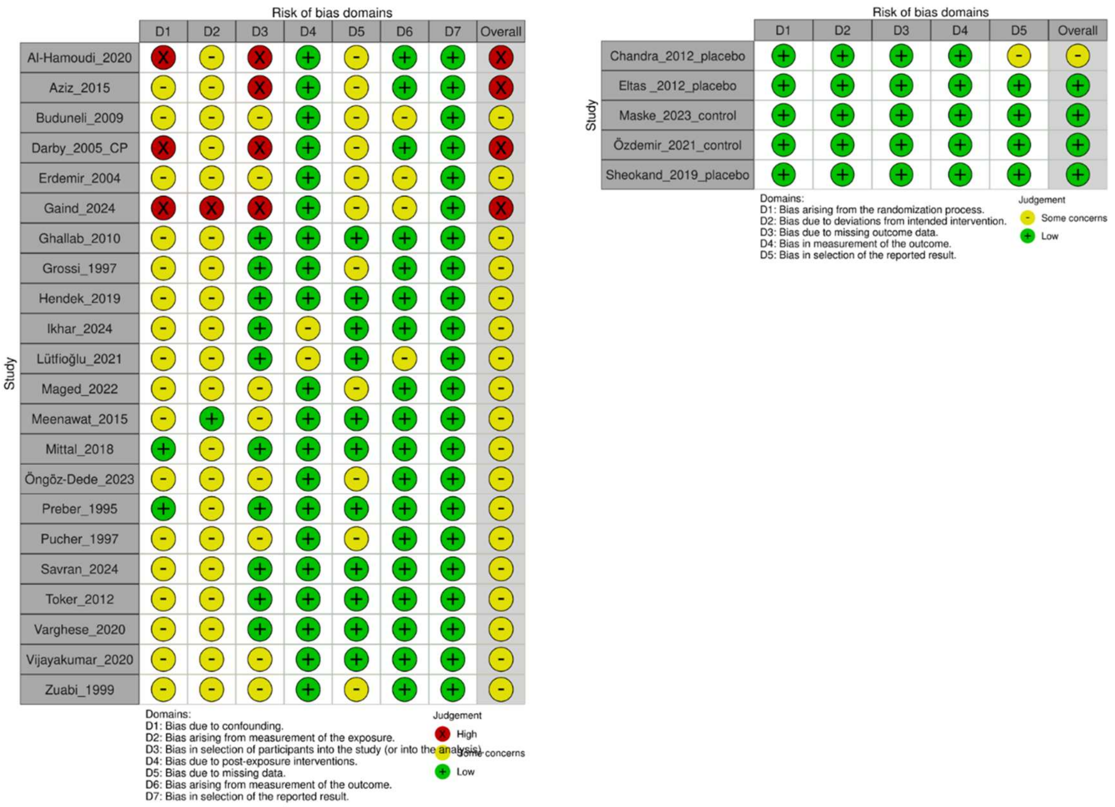

Figure S2.5. Risk of bias assessment of included articles for gingival index (GI). ROBINS-E was used for assessing risk of bias in non-randomized studies, RoB 2 tool was used to assess risk of bias in randomized controlled trials.

FMPS

ROBINS-E:

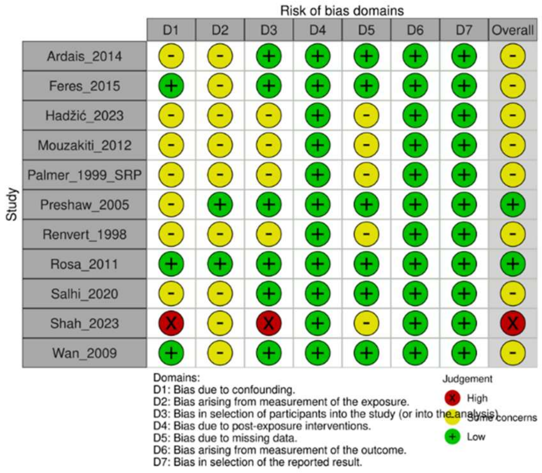

RoB 2.0:

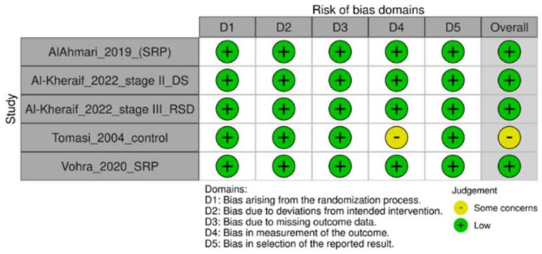

Figure S2.6. Risk of bias assessment of included articles for full mouth plaque score (FMPS). ROBINS-E was used for assessing risk of bias in non-randomized studies, RoB 2 tool was used to assess risk of bias in randomized controlled trials.

GCF volume

ROBINS-E:

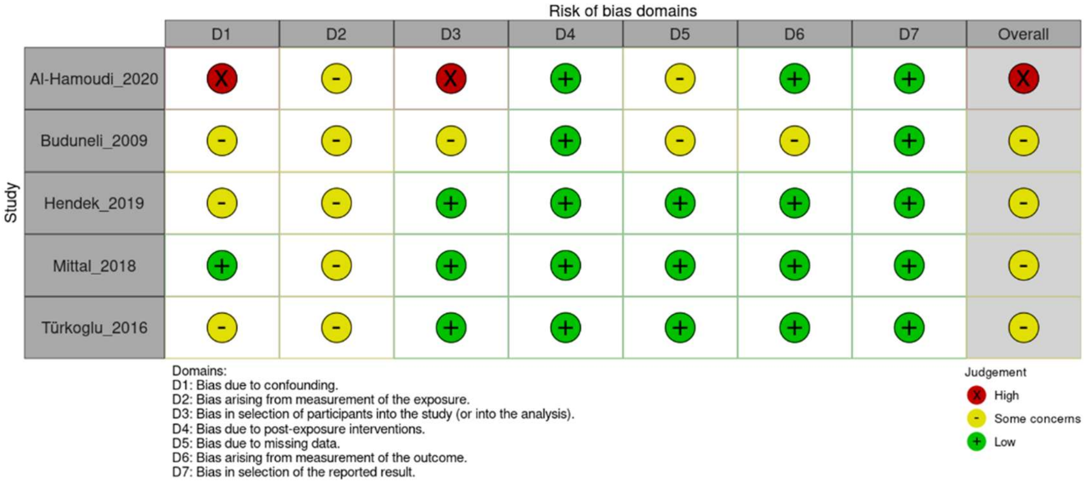

RoB 2.0:

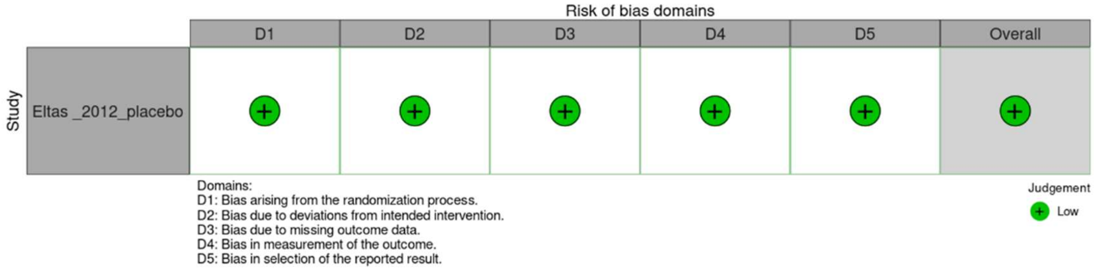

Figure S2.7. Risk of bias assessment of included articles for gingival crevicular fluid (GCF) volume. ROBINS-E was used for assessing risk of bias in non-randomized studies, RoB 2 tool was used to assess risk of bias in randomized controlled trials.
