# Supplementary material for: Impact of Traditional and Alternative Nicotine-Containing Product Use on Non-Surgical Periodontal Therapy: A Systematic Review and Network Meta-Analysis
Source: Dent J (Basel). 2026 Sep 22;14(10):616. doi: 10.3390/dj14100616 (PMC13703454; doi:10.3390/dj14100616)
Supplement: Supplementary file 1 [file dentistry-14-00616-s001.zip › Document S3 (Networks).pdf]

Network plots and characteristic tables of each analysis

A network plot was created for each of the groups to check whether the resulting network is fully connected, or not. The network plots of the eligible groups applicable for NMA are presented below.

The basic characteristics of each analysis were reported using network characteristic tables, containing the most important statistics from each subgroup.

1. Change in probing pocket depth (PPD)

Characteristic	*Value*
Number of Interventions	6
Number of Studies	52
Total Number of Patients in Network	3209
Total Possible Pairwise Comparison	15
Total Number of Pairwise Comparison with Direct Data	9
Number of Two-Arm Studies	47
Number of Multi-Arms Studies	5

Exposure	Number of studies	Number of patients	Average outcome
**CS**	50	1359	0.995
**EC**	2	56	1.1
**FS**	4	267	0.464
**NS**	50	1393	1.38
**Q**	2	52	0.963
**ST**	2	82	0.857

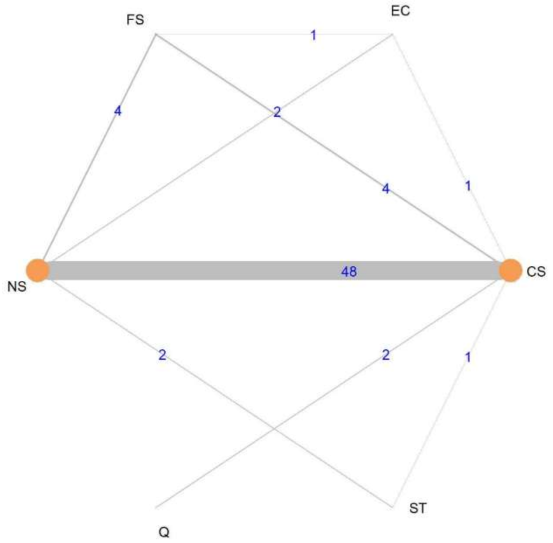

Figure S3.1. *Network plot and network characteristic table of probing pocket depth*; NS, non-smoker; CS, cigarette smoker; Q, quitter; FS, former smoker; EC, electronic cigarette; ST, smokeless tobacco

2. Change in clinical attachment loss (CAL)

Characteristic	*Value*
Number of Interventions	6
Number of Studies	44
Total Number of Patients in Network	2323
Total Possible Pairwise Comparison	15
Total Number of Pairwise Comparison with Direct Data	8
Number of Two-Arm Studies	40
Number of Multi-Arms Studies	4

Exposure	Number of studies	Number of patients	Average outcome
**CS**	42	958	0.821
**EC**	2	56	0.164
**FS**	4	212	0.760
**NS**	42	1038	0.933
**Q**	2	27	0.445
**ST**	1	32	-0.100

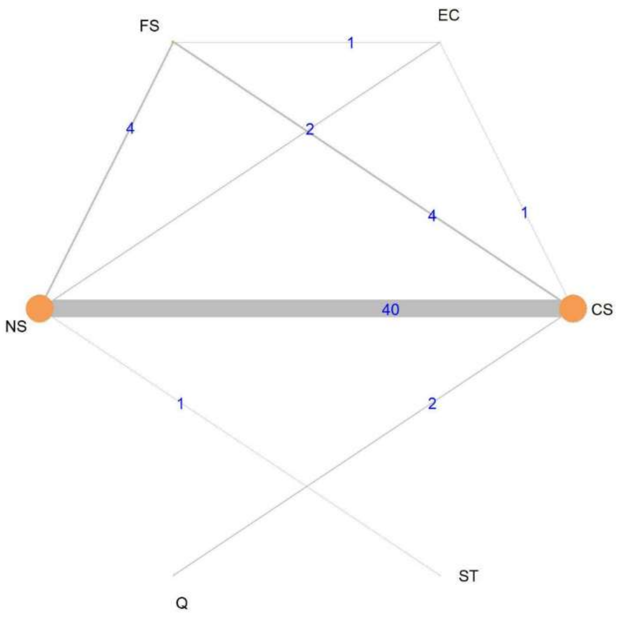

Figure S3.2. *Network plot and network characteristic table of clinical attachment loss;* NS, non-smoker; CS, cigarette smoker; Q, quitter; FS, former smoker; EC, electronic cigarette; ST, smokeless tobacco

3. Change in bleeding on probing (BOP)

Characteristic	*Value*
Number of Interventions	6
Number of Studies	25
Total Number of Patients in Network	1231
Total Possible Pairwise Comparison	15
Total Number of Pairwise Comparison with Direct Data	8
Number of Two-Arm Studies	23
Number of Multi-Arms Studies	2

Exposure	Number of studies	Number of patients	Average outcome
**CS**	24	473	23.6
**EC**	1	20	7.6
**FS**	2	85	31.8
**NS**	23	594	33.4
**Q**	2	27	10.4
**ST**	1	32	12.8

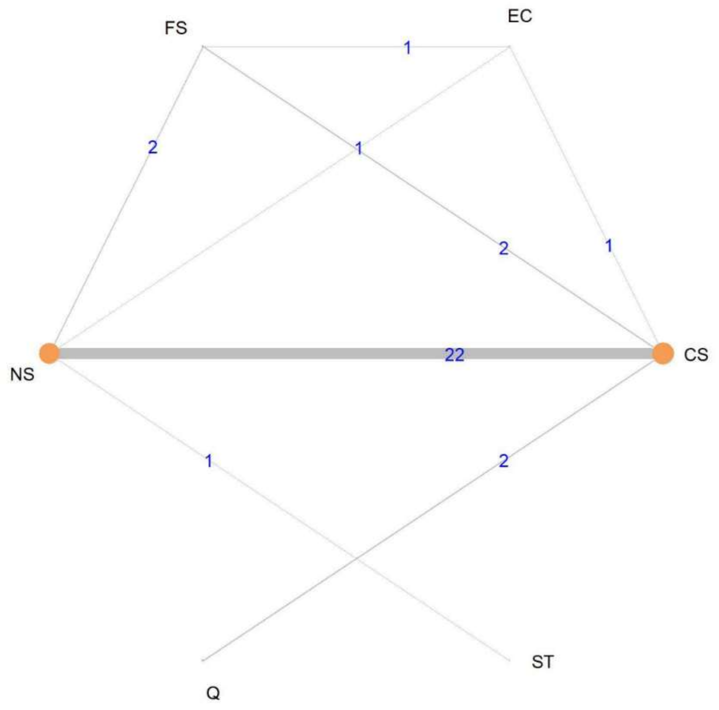

Figure S3.3. *Network plot and network characteristic table of bleeding on probing;* NS, non-smoker; CS, cigarette smoker; Q, quitter; FS, former smoker; EC, electronic cigarette; ST, smokeless tobacco

4. Change in plaque index (PI)

Characteristic	*Value*
Number of Interventions	5
Number of Studies	32
Total Number of Patients in Network	1671
Total Possible Pairwise Comparison	10
Total Number of Pairwise Comparison with Direct Data	6
Number of Two-Arm Studies	30
Number of Multi-Arms Studies	2

**Exposure**	**Number of studies**	**Number of patients**	**Average outcome**
**CS**	31	770	0.89
**EC**	1	36	0.71
**FS**	1	55	0.69
**NS**	32	759	0.91
**ST**	1	50	0.38

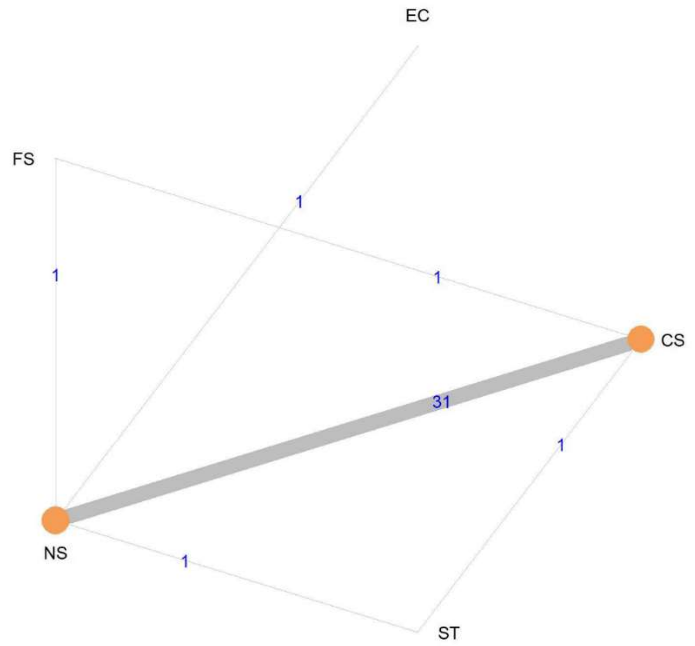

Figure S3.4. *Network plot and network characteristic table of plaque index;* NS, non-smoker; CS, cigarette smoker; FS, former smoker; EC, electronic cigarette; ST, smokeless tobacco

5. Change in gingival index (GI)

Characteristic	*Value*
Number of Interventions	4
Number of Studies	26
Total Number of Patients in Network	1294
Total Possible Pairwise Comparison	6
Total Number of Pairwise Comparison with Direct Data	4
Number of Two-Arm Studies	25
Number of Multi-Arms Studies	1

Exposure	Number of studies	Number of patients	Average outcome
**CS**	25	592	0.78
**EC**	1	36	0.02
**NS**	26	616	1.09
**ST**	1	50	0.32

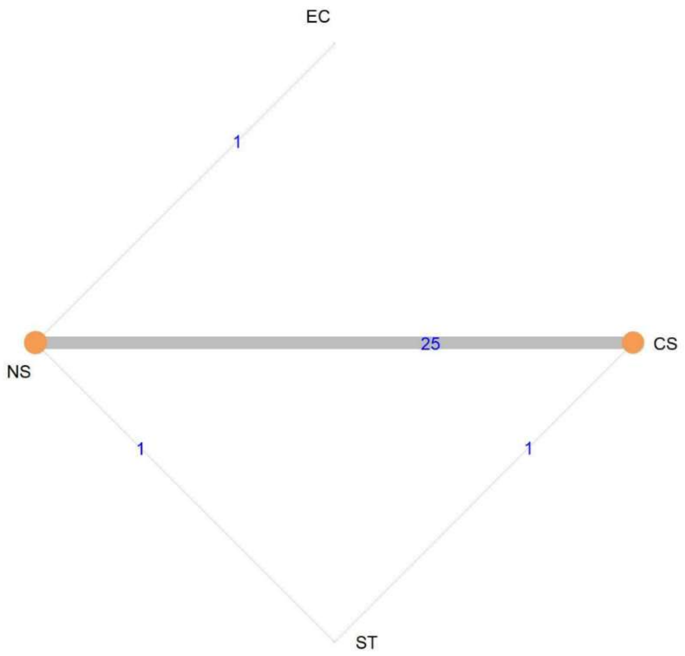

Figure S3.5. *Network plot and network characteristic table of gingival index;* NS, non-smoker; CS, cigarette smoker; EC, electronic cigarette; ST, smokeless tobacco

6. Change in full mouth plaque score (FMPS)

Characteristic	*Value*
Number of Interventions	6
Number of Studies	15
Total Number of Patients in Network	740
Total Possible Pairwise Comparison	15
Total Number of Pairwise Comparison with Direct Data	8
Number of Two-Arm Studies	13
Number of Multi-Arms Studies	2

Exposure	Number of studies	Number of patients	Average outcome
**CS**	14	239	29.6
**EC**	1	20	20.8
**FS**	2	85	37.9
**NS**	13	337	32.2
**Q**	2	27	22.0
**ST**	1	32	11.1

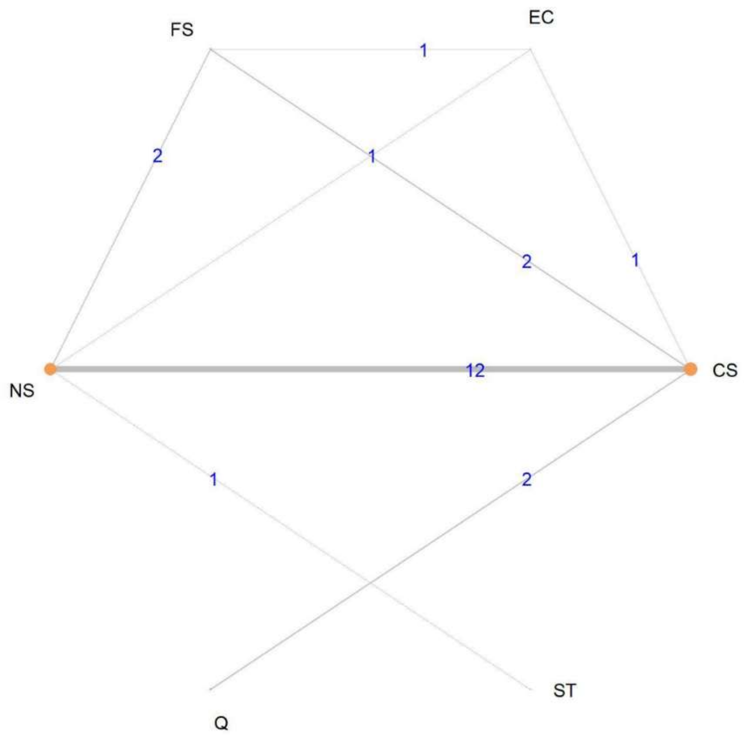

Figure S3.6. *Network plot and network characteristic table of full mouth plaque score (%)* NS, non-smoker; CS, cigarette smoker; Q, quitter; FS, former smoker; EC, electronic cigarette; ST, smokeless tobacco

7. Change in gingival crevicular fluid volume

Characteristic	*Value*
Number of Interventions	4
Number of Studies	6
Total Number of Patients in Network	364
Total Possible Pairwise Comparison	6
Total Number of Pairwise Comparison with Direct Data	4
Number of Two-Arm Studies	5
Number of Multi-Arms Studies	1

Exposure	Number of studies	Number of patients	Average outcome
**CS**	5	122	0.164
**EC**	1	36	0.99
**NS**	6	156	0.551
**ST**	1	50	0.002

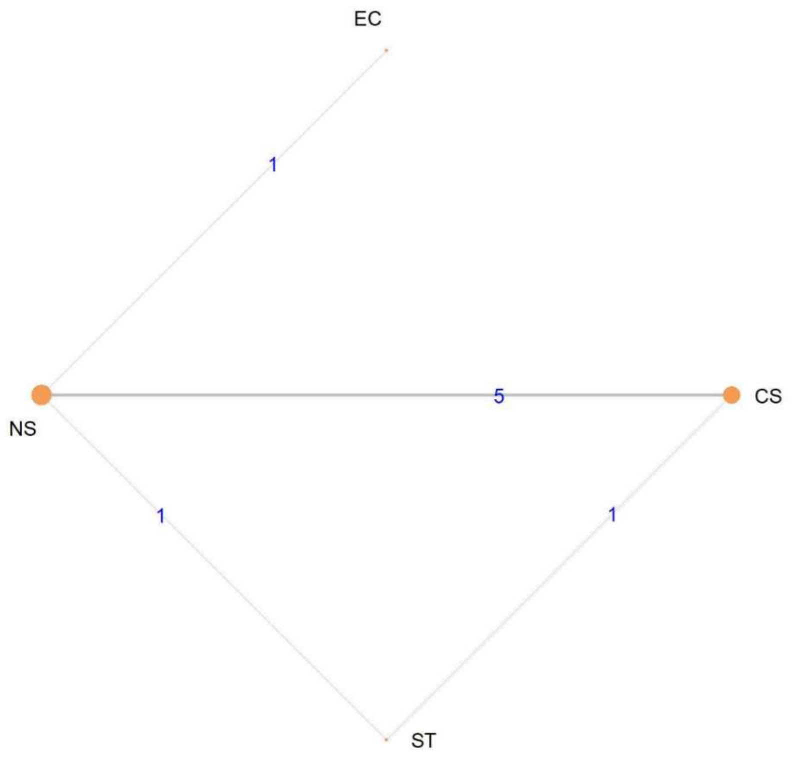

Figure S3.7. *Network plot and network characteristic table of gingival crevicular fluid volume;* NS, non-smoker; CS, cigarette smoker; EC, electronic cigarette; ST, smokeless tobacco
