# Supplementary material for: Impact of Traditional and Alternative Nicotine-Containing Product Use on Non-Surgical Periodontal Therapy: A Systematic Review and Network Meta-Analysis
Source: Dent J (Basel). 2026 Sep 22;14(10):616. doi: 10.3390/dj14100616 (PMC13703454; doi:10.3390/dj14100616)
Supplement: Supplementary file 1 [file dentistry-14-00616-s001.zip › Document S4 (Additional figures).pdf]

PPD

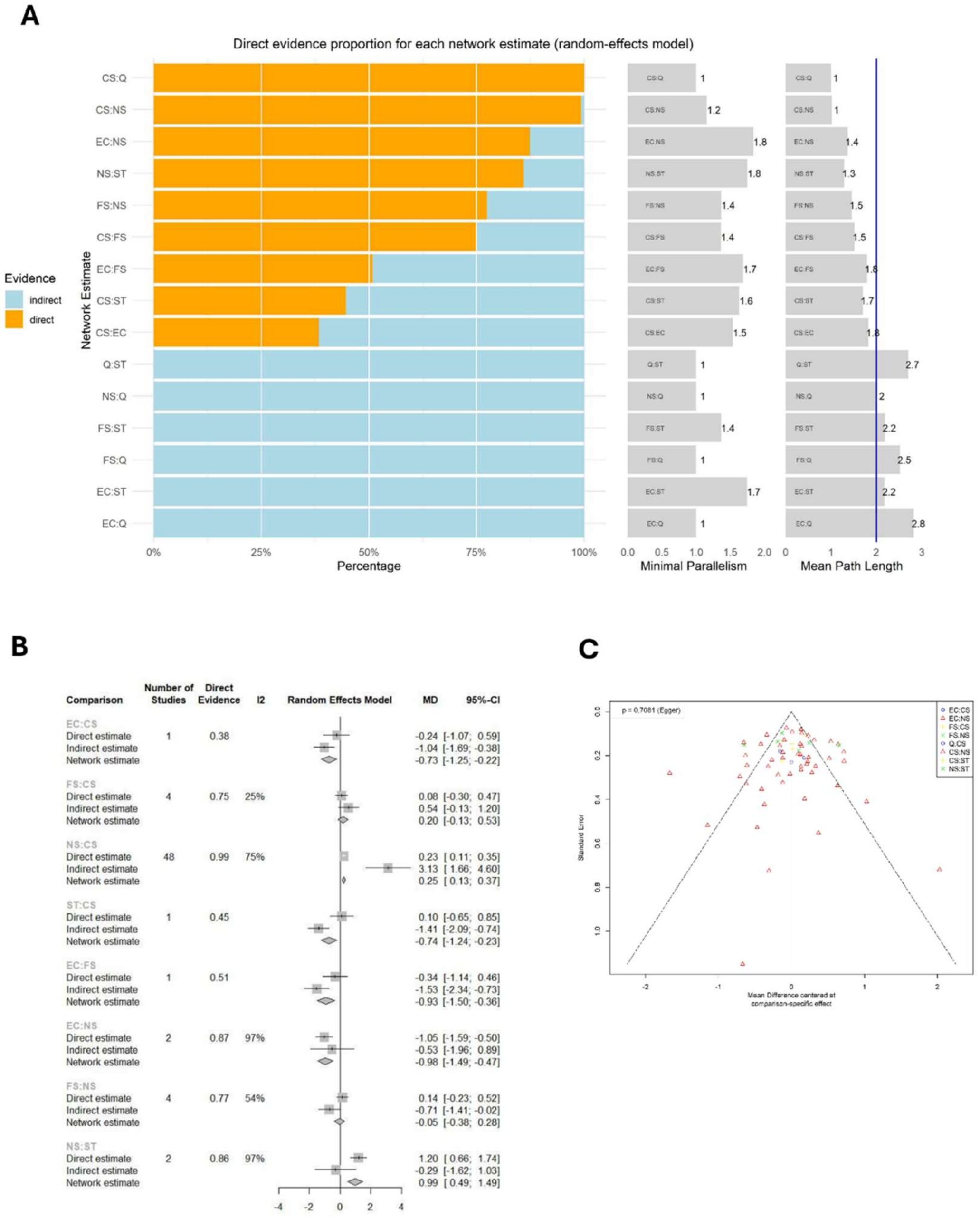

Figure S4.1. Network meta-analysis diagnostics and results for probing pocket depth (PPD); A, Evidence plot showing direct and indirect evidence proportion for each network estimate; B, Forest plot showing direct, indirect, and network estimates for each pairwise comparison to assess consistency-inconsistency; C, Funnel plot to assess small-study effects; NS, non-smoker; CS, cigarette smoker; Q, quitter; FS, former smoker; EC, electronic cigarette; ST, smokeless tobacco

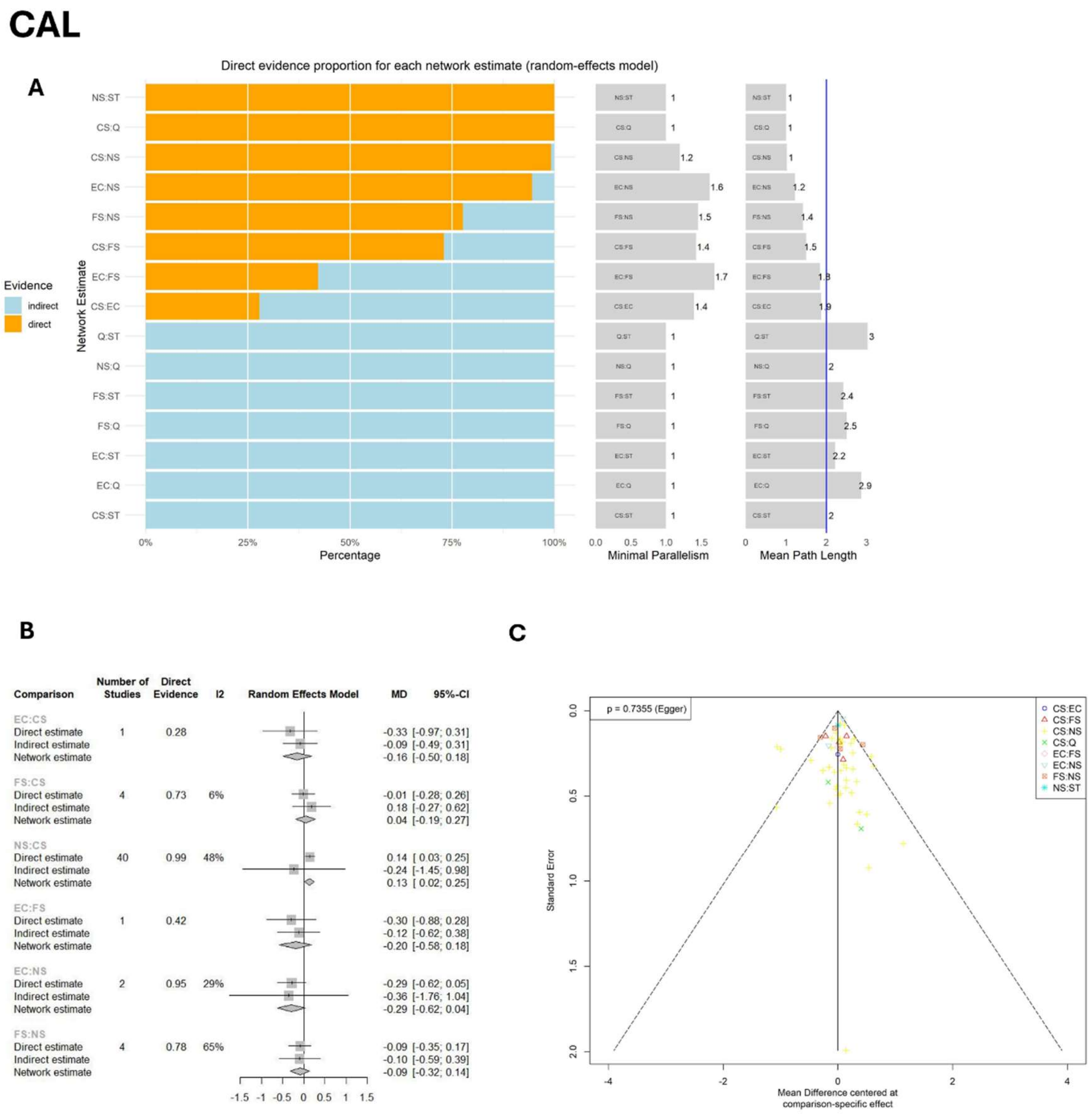

Figure S4.2. Network meta-analysis diagnostics and results for clinical attachment loss (CAL); A, Evidence plot showing direct and indirect evidence proportion for each network estimate; B, Forest plot showing direct, indirect, and network estimates for each pairwise comparison to assess consistency-inconsistency; C, Funnel plot to assess small-study effects; NS, non-smoker; CS, cigarette smoker; Q, quitter; FS, former smoker; EC, electronic cigarette; ST, smokeless tobacco

BOP

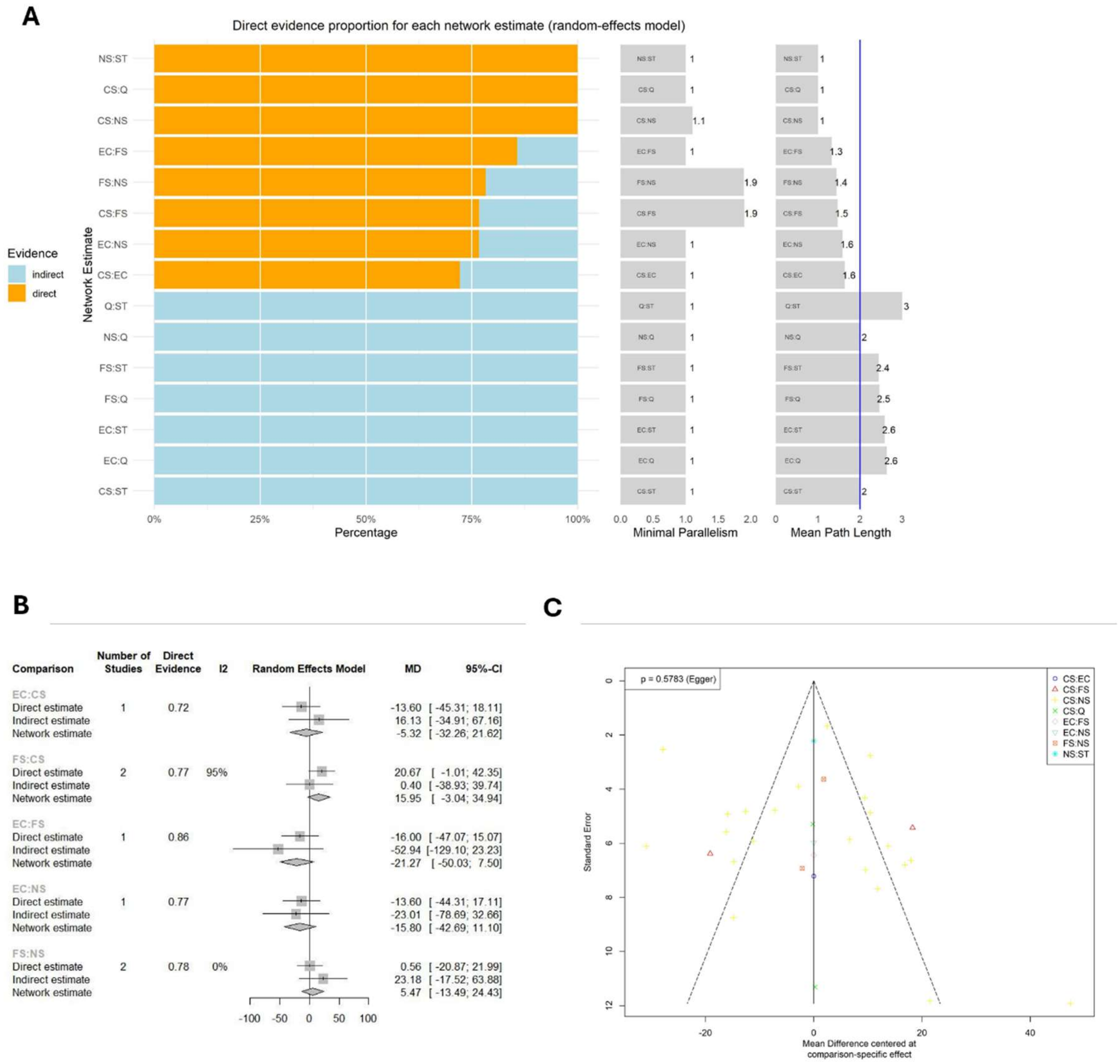

Figure S4.3. Network meta-analysis diagnostics and results for bleeding on probing (BOP); A, Evidence plot showing direct and indirect evidence proportion for each network estimate; B, Forest plot showing direct, indirect, and network estimates for each pairwise comparison to assess consistency-inconsistency; C, Funnel plot to assess small-study effects; NS, non-smoker; CS, cigarette smoker; Q, quitter; FS, former smoker; EC, electronic cigarette; ST, smokeless tobacco

Δ PI

A

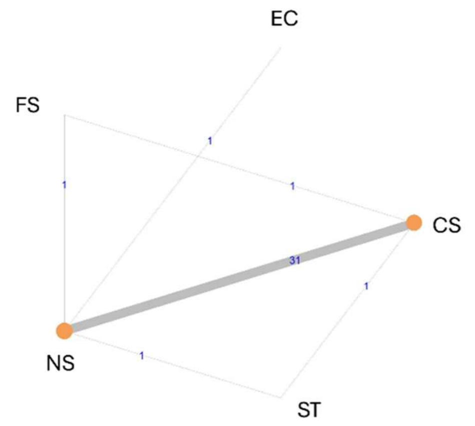

B

P-score (random)	
FS	0.8516
CS	0.7026
NS	0.4904
ST	0.4512
EC	0.0041

C

FS	0.15 [-0.42; 0.72]	0.28 [-0.32; 0.88]	.	.
0.18 [-0.32; 0.68]	CS	0.06 [-0.05; 0.17]	0.11 [-0.46; 0.68]	.
0.24 [-0.26; 0.74]	0.06 [-0.05; 0.17]	NS	0.13 [-0.44; 0.70]	0.92 [0.37; 1.47]
0.33 [-0.38; 1.04]	0.15 [-0.35; 0.65]	0.09 [-0.41; 0.59]	ST	.
1.16 [0.42; 1.90]	0.98 [0.42; 1.54]	0.92 [0.37; 1.47]	0.83 [0.08; 1.57]	EC

Figure S4.4. A, Network geometry of the eligible comparisons of change in plaque index (PI) in different nicotine-containing product user groups and non-smokers; B, Treatment ranking with P-scores under the random-effects model where higher values indicate larger outcome values, associated with favorable outcome between baseline and re-evaluation. C, League table shows the mean difference and 95% confidence interval for direct (upper right triangle) and network (lower left triangle) comparisons; NS, non-smokers; Q, quitters; FS, former smokers; CS, cigarette smokers; EC, electronic cigarette; ST, smokeless tobacco.

PI

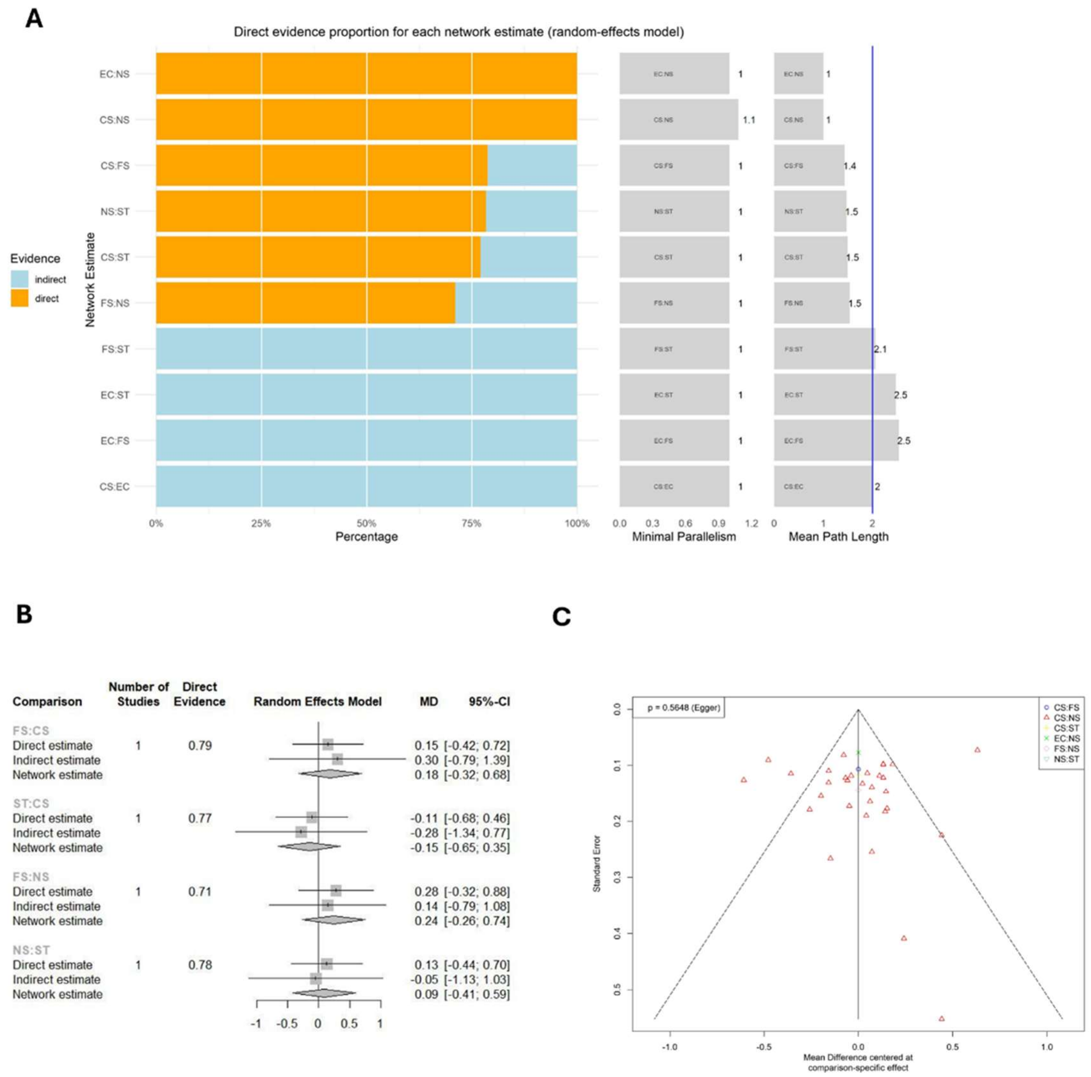

Figure S4.5. Network meta-analysis diagnostics and results for plaque index (PI); A, Evidence plot showing direct and indirect evidence proportion for each network estimate; B, Forest plot showing direct, indirect, and network estimates for each pairwise comparison to assess consistency-inconsistency; C, Funnel plot to assess small-study effects; NS, non-smoker; CS, cigarette smoker; FS, former smoker; EC, electronic cigarette; ST, smokeless tobacco

Δ GI

A

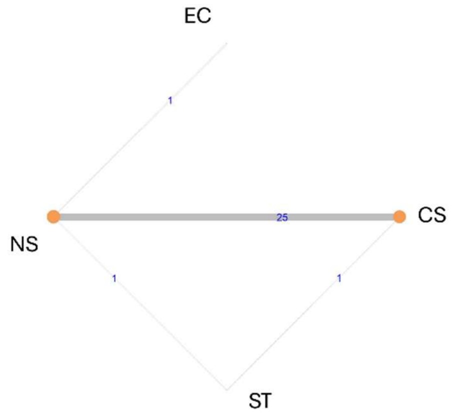

B

P-score (random)	
NS	0.9828
CS	0.5636
ST	0.4534
EC	0.0002

C

NS	0.30 [0.17; 0.42]	0.72 [0.14; 1.30]	1.67 [1.12; 2.22]
0.30 [0.17; 0.42]	CS	-0.15 [-0.73; 0.43]	.
0.43 [-0.09; 0.94]	0.13 [-0.38; 0.64]	ST	.
1.67 [1.12; 2.22]	1.37 [0.81; 1.93]	1.24 [0.49; 1.99]	EC

Figure S4.6. A, Network geometry of the eligible comparisons of change in gingival index (GI) in different nicotine-containing product user groups and non-smokers; B, Treatment ranking with P-scores under the random-effects model where higher values indicate larger outcome values, associated with favorable outcome between baseline and re-evaluation. C, League table shows the mean difference and 95% confidence interval for direct (upper right triangle) and network (lower left triangle) comparisons; NS, non-smokers; CS, cigarette smokers; EC, electronic cigarette; ST, smokeless tobacco.

GI

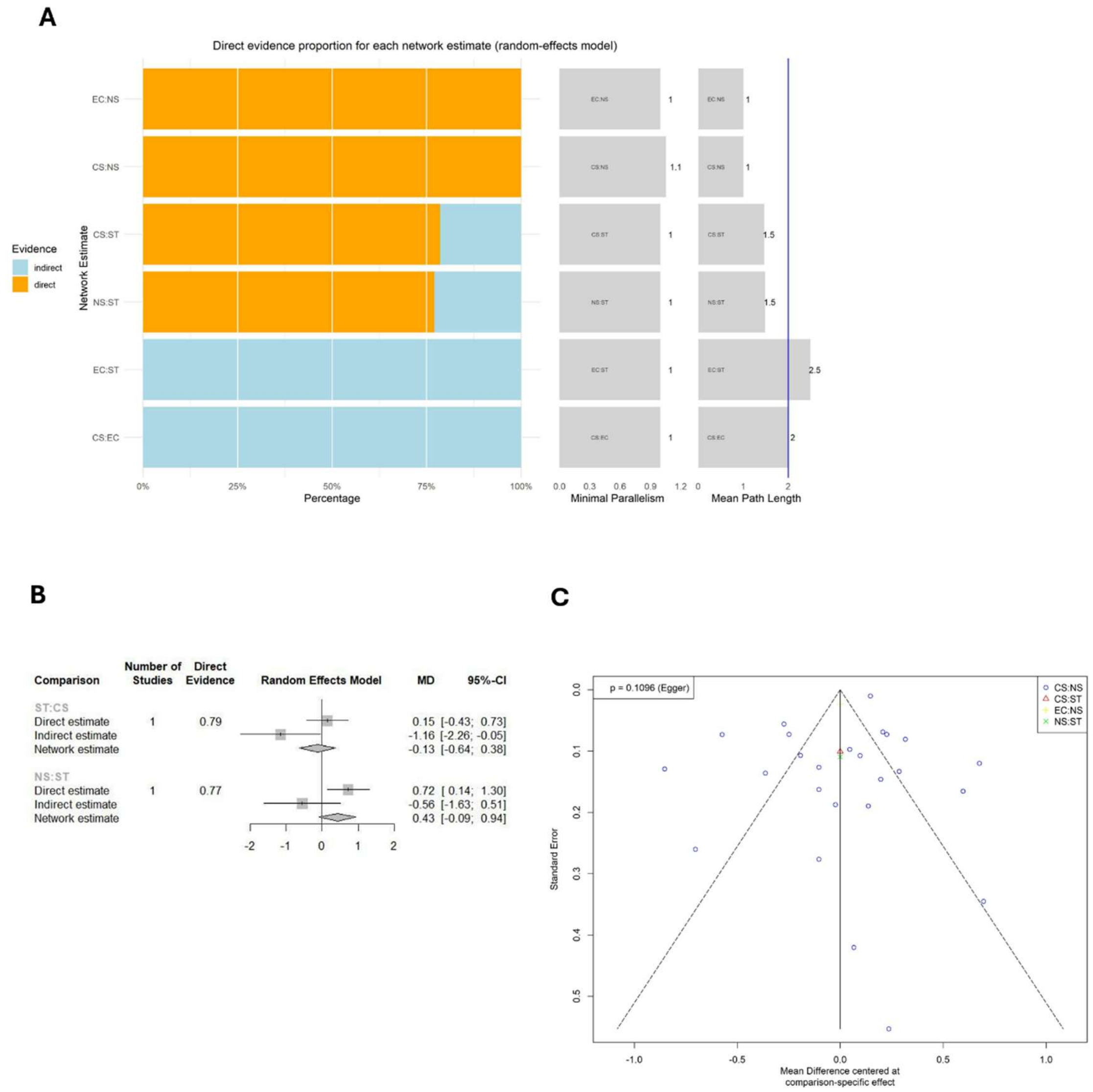

Figure S4.7. Network meta-analysis diagnostics and results for gingival index (GI); A, Evidence plot showing direct and indirect evidence proportion for each network estimate; B, Forest plot showing direct, indirect, and network estimates for each pairwise comparison to assess consistency-inconsistency; C, Funnel plot to assess small-study effects; NS, non-smoker; CS, cigarette smoker; EC, electronic cigarette; ST, smokeless tobacco

Δ FMPS (%)

A

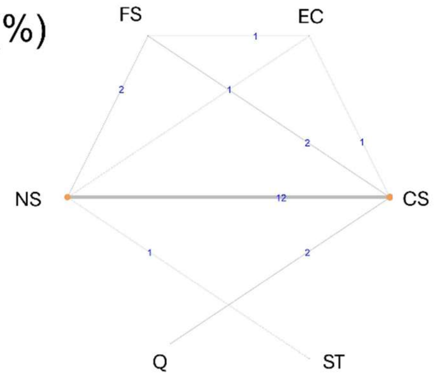

B

P-score (random)	
Q	0.7540
FS	0.6683
NS	0.6166
CS	0.4002
EC	0.3980
ST	0.1629

C

Q	.	.	8.14 [-10.57; 26.86]	.	.
3.51 [-19.40; 26.42]	FS	-0.19 [-14.70; 14.31]	6.82 [-8.38; 22.02]	3.90 [-17.78; 25.58]	.
5.24 [-14.49; 24.98]	1.73 [-11.35; 14.81]	NS	2.86 [-3.40; 9.13]	5.20 [-16.31; 26.71]	12.00 [-6.94; 30.94]
8.14 [-10.57; 26.86]	4.63 [-8.58; 17.85]	2.90 [-3.36; 9.16]	CS	3.50 [-19.59; 26.59]	.
9.68 [-17.34; 36.69]	6.17 [-14.16; 26.50]	4.44 [-14.91; 23.78]	1.54 [-17.94; 21.02]	EC	.
17.24 [-10.11; 44.59]	13.73 [-9.29; 36.75]	12.00 [-6.94; 30.94]	9.10 [-10.85; 29.05]	7.56 [-19.51; 34.64]	ST

Figure S4.8. A, Network geometry of the eligible comparisons of change in full mouth plaque score (FMPS) in different nicotine-containing product user groups and non-smokers; B, Treatment ranking with P-scores under the random-effects model where higher values indicate larger outcome values, associated with favorable outcome between baseline and re-evaluation. C, League table shows the mean difference and 95% confidence interval for direct (upper right triangle) and network (lower left triangle) comparisons; NS, non-smokers; Q, quitters; FS, former smokers; CS, cigarette smokers; EC, electronic cigarette; ST, smokeless tobacco.

FMPS (%)

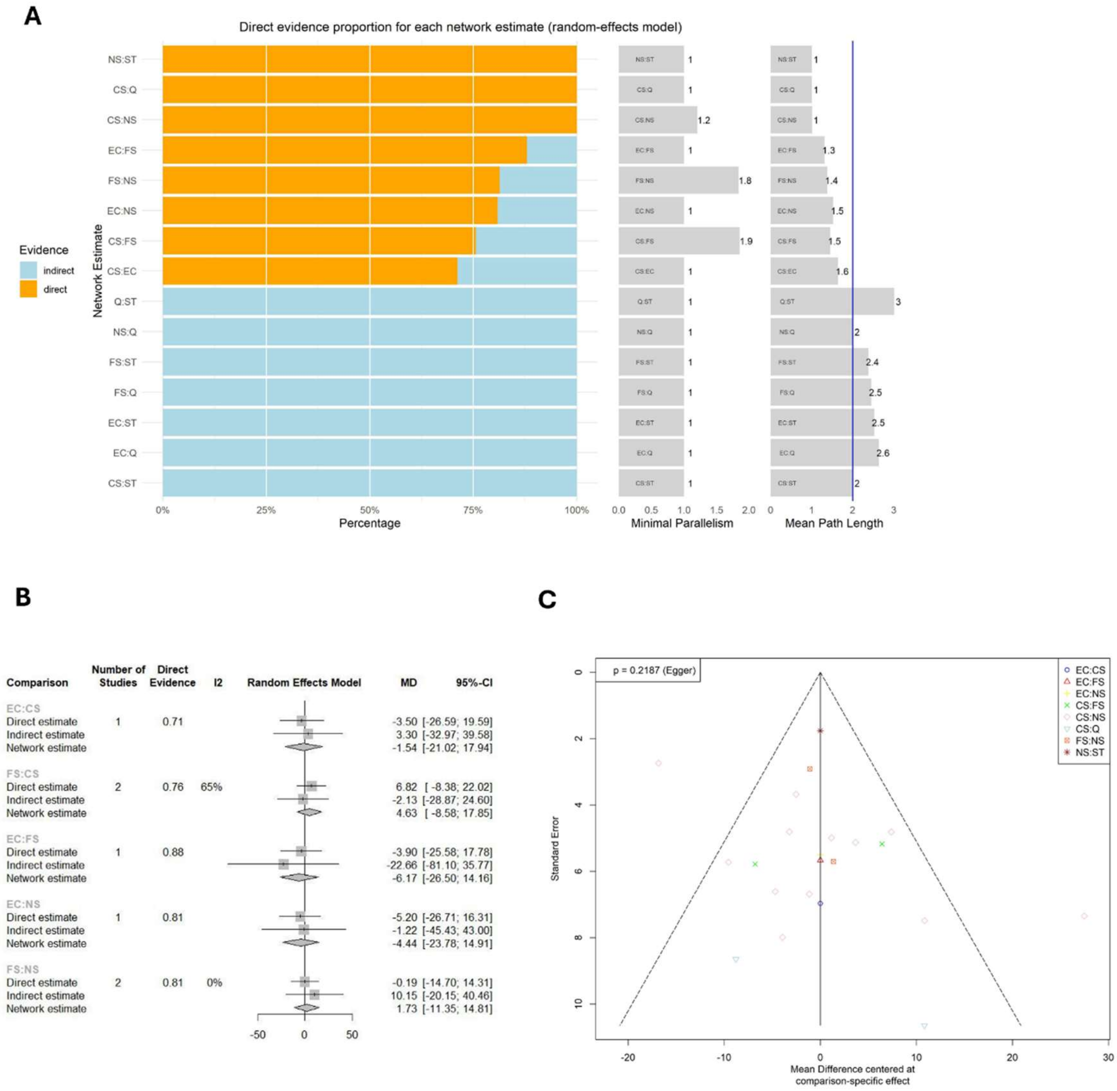

Figure S4.9. Network meta-analysis diagnostics and results for full mouth plaque score (FMPS); A, Evidence plot showing direct and indirect evidence proportion for each network estimate; B, Forest plot showing direct, indirect, and network estimates for each pairwise comparison to assess consistency-inconsistency; C, Funnel plot to assess small-study effects; NS, non-smoker; CS, cigarette smoker; Q, quitter; FS, former smoker; EC, electronic cigarette; ST, smokeless tobacco

Δ GCF volume

A

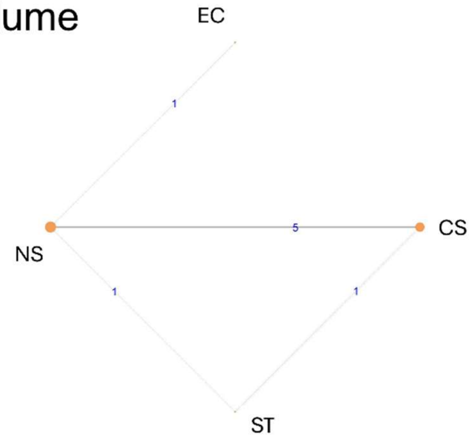

B

P-score (random)	
NS	0.9194
ST	0.6481
CS	0.4323
EC	0.0002

C

NS	0.02 [-0.15; 0.19]	0.11 [0.01; 0.21]	0.57 [0.32; 0.82]
0.06 [-0.10; 0.22]	ST	0.00 [-0.17; 0.17]	.
0.11 [0.01; 0.21]	0.05 [-0.11; 0.20]	CS	.
0.57 [0.32; 0.82]	0.51 [0.22; 0.80]	0.46 [0.20; 0.73]	EC

Figure S4.10. A, Network geometry of the eligible comparisons of change in gingival crevicular fluid volume (GCF) in different nicotine-containing product user groups and non-smokers; B, Treatment ranking with P-scores under the random-effects model where higher values indicate larger outcome values, associated with favorable outcome between baseline and re-evaluation. C, League table shows the mean difference and 95% confidence interval for direct (upper right triangle) and network (lower left triangle) comparisons; NS, non-smokers; CS, cigarette smokers; EC, electronic cigarette; ST, smokeless tobacco.

GCF

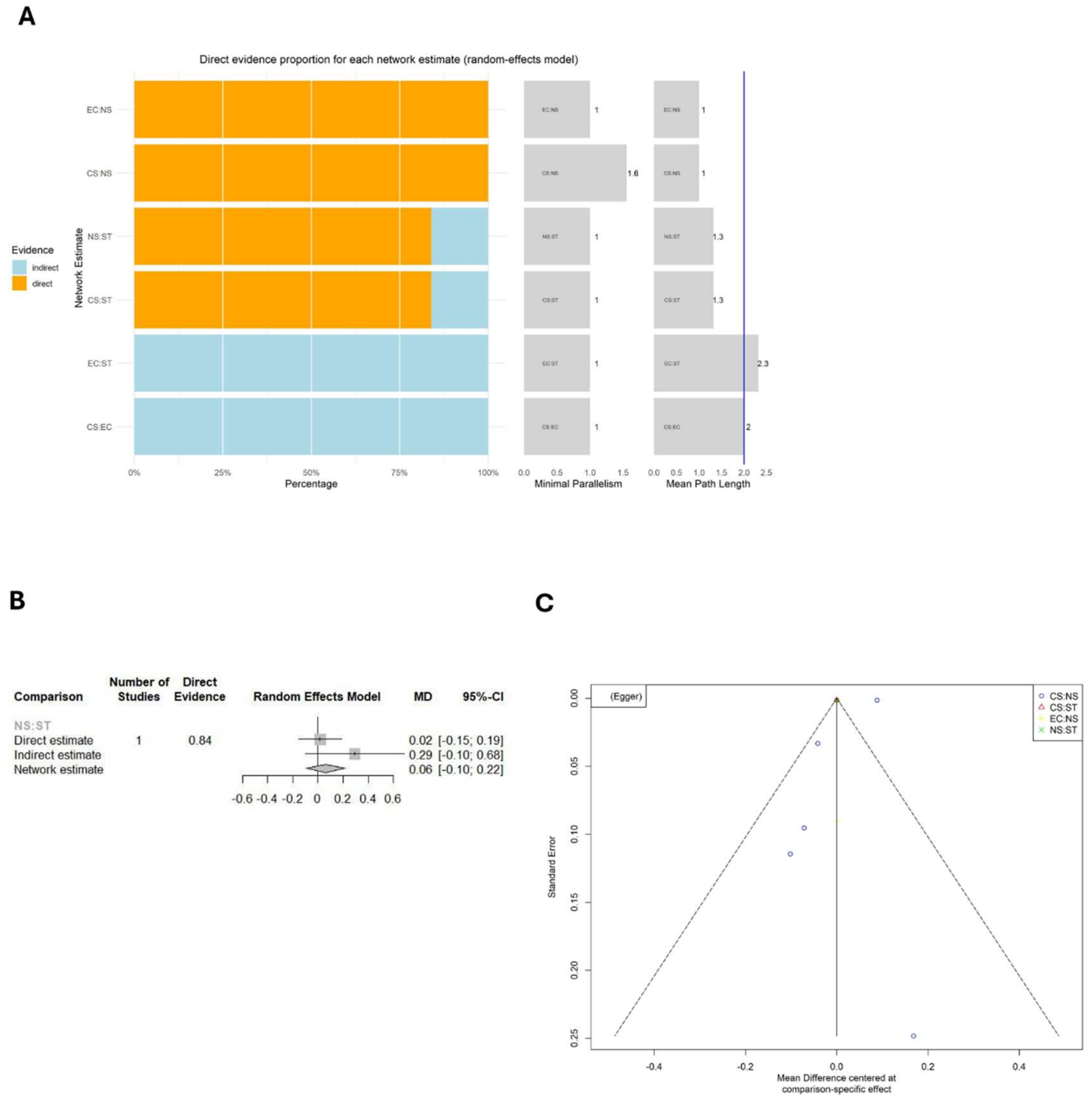

Figure S4.11. Network meta-analysis diagnostics and results for gingival crevicular fluid volume (GCF); A, Evidence plot showing direct and indirect evidence proportion for each network estimate; B, Forest plot showing direct, indirect, and network estimates for each pairwise comparison to assess consistency-inconsistency; C, Funnel plot to assess small-study effects; NS, non-smoker; CS, cigarette smoker; FS, former smoker; EC, electronic cigarette; ST, smokeless tobacco
