# Supplementary material for: Impact of Traditional and Alternative Nicotine-Containing Product Use on Non-Surgical Periodontal Therapy: A Systematic Review and Network Meta-Analysis
Source: Dent J (Basel). 2026 Sep 22;14(10):616. doi: 10.3390/dj14100616 (PMC13703454; doi:10.3390/dj14100616)
Supplement: Supplementary file 1 [file dentistry-14-00616-s001.zip › Document S5 (Subgroup).pdf]

Results of the subset analyses

A subset analysis based on the examined sites was performed. Subset 0 included studies, where full mouth indices were measured, while subset 1 included studies where only deep pockets were examined.

Author	Examined site	Subset	Outcome
**AlAhmari et al. (2019) (SRP alone)**	full mouth: at 6 sites per tooth on all maxillary and mandibular teeth	0	BOP PPD CAL
**Al-Hamoudi et al. (2020)**	full mouth: at 6 sites per tooth excluding third molars	0	PI GI PPD CAL
**Al-Kheraif et al. (2022) (RSD alone)**	split-mouth design, at 6 sites of the tooth on each tooth	0	BOP PPD CAL
**Al-Kheraif et al. (2022) (DS alone)**	split-mouth design, at 6 sites of the tooth on each tooth	0	BOP PPD CAL
**Apatzidou et al (2005)**	at 6 sites/tooth;	0	BOP PPD CAL
	1 site/quadrant with the deepest PPD, (not less than 5mm), from each patient for selected site clinical analysis.	1	PPD
**Ardais et al. (2014)**	full mouth: at 6 sites per tooth excluding third molars	0	BOP PPD CAL
**Aziz et al. (2015)**	full mouth: six sites around each tooth	0	PI GI PPD CAL
**Buduneli et al. (2009)**	sampling sites were PPD≥5 mm, CAL≥4 mm, and the presence of BOP in single-rooted teeth	1	PI GI PPD CAL
**Chandra et al. (2012) (placebo)**	interdental suprabony periodontal pockets in molars with PPD ≥5 mm	1	GI PI PPD CAL
**D'Aiuto et al. (2005)**	full mouth: at 6 sites per tooth	0	PPD
**Darby et al. (2005) (CP)**	In each patient, 4 sites with PPD≥5 mm were selected, where possible one in each quadrant, with no furcation involvement.	1	GI PI PPD
**Do et al. (2023)**	full mouth	0	PPD CAL
**Eltas et al. (2012) (placebo)**	test sites: PPD between 4 and 6 mm	1	GI PI PPD CAL
**Erdemir et al. (2004)**	full mouth	0	GI PI BOP
	sample sites (4 sites of each patient with PPD≥5 mm)	1	PPD CAL
**Feres et al. (2015)**	full mouth: at 6 sites per tooth excluding third molars	0	BOP PPD CAL
**Gaind et al. (2024)**	full mouth	0	PI GI
**Ghallab et al. (2010)**	full mouth: at 6 sites per tooth excluding third molars	0	PI GI
**Grossi et al. (1997)**	full mouth: at 6 sites per tooth excluding third molars	0	GI PI PPD CAL
	PPD≥5 mm	1	PPD CAL
**Guentsch et al. (2008)**	full mouth: at 6 sites per tooth	0	BOP PPD

**Guru et al. (2018)**	full mouth	0	PI<br>PPD<br>CAL
**Hadžić et al. (2023)**	full mouth: at 6 sites per tooth	0	BOP<br>PPD<br>CAL
**Hendek et al. (2019)**	full mouth: at 6 sites per tooth;	0	GI<br>PI<br>PPD<br>CAL
	sample teeth: single-rooted teeth with ≥4 and <7 mm PPD and ≥30% bone loss	1	GI<br>PI<br>PPD<br>CAL
**Ikhar et al. (2024)**	full mouth: at 6 sites per tooth	0	GI<br>PI<br>PPD<br>CAL
**Lütfioğlu et al. (2021)**	full mouth: at 6 sites per tooth, each highest value per tooth was taken for evaluations	0	GI<br>PI<br>BOP<br>PPD<br>CAL
**Maged et al. (2022)**	full mouth: at 6 sites per tooth	0	PI<br>GI<br>PPD<br>CAL
**Mantyla et al. (2006)**	7–10 sites/patient with PPD ≥4 mm	0	PPD<br>CAL
**Mashalkar et al. (2024)**	full mouth	0	PI<br>BOP<br>PPD<br>CAL
**Maske et al. (2023) (control)**	split mouth study, total 80 sites with PPD≥ 5 mm	1	PI<br>GI<br>PPD<br>CAL
**Meenawat et al. (2015)**	full mouth	0	PI<br>GI<br>PPD<br>CAL
**Mittal et al. (2018)**	full mouth	0	PI<br>GI<br>PPD
**Mouzakiti et al. (2012)**	full mouth	0	PPD<br>BOP
	sampling site	1	PPD<br>CAL
**Öngöz-Dede et al. (2023)**	full mouth: six sites around each tooth	0	PI<br>GI<br>BOP<br>PPD<br>CAL
**Özdemir et al. (2021) (control)**	full mouth: on six surfaces of each tooth except third molars	0	PI<br>GI<br>PPD<br>CAL
**Palmer et al. (1999) (SRP)**	sites with baseline probing depths equal to or greater than 5 mm	1	BOP<br>PPD
**Preber et al. (1986)**	full mouth: on four sites of all teeth	0	PI<br>BOP
	pathological pockets (≥4 mm)	1	PI<br>BOP
**Preber et al. (1985)**	full mouth	0	PI<br>PPD
**Preber et al. (1995)**	full mouth and	0	GI<br>PI<br>PPD
	1 site with a PPD of more than 5 mm	1	PPD
**Preshaw et al. (2005)**	full mouth	0	BOP<br>PPD
	diseased sites (>3 mm)	1	PPD<br>CAL

**Pucher et al. (1997)**	target sites<br>(PPD 5-8 mm, bleeding upon probing)	1	PI<br>GI<br>PPD<br>CAL
**Renvert et al. (1998)**	full mouth	0	BOP
	experimental sites	1	PI<br>BOP<br>PPD
**Rosa et al. (2011)**	full mouth	0	BOP<br>CAL<br>PPD
	sites presenting baseline PPD ≥4mm	1	PPD<br>CAL
**Salhi et al. (2020)**	full mouth: at 6 sites of each tooth	0	BOP<br>CAL<br>PPD
**Savran et al. (2024) (control)**	full mouth: at 6 sites of each tooth	0	PI<br>GI<br>BOP<br>PPD<br>CAL
**Shah et al. (2023)**	full mouth	0	BOP<br>CAL<br>PPD
**Sheokand et al. (2019) (placebo)**	15-15 sites with PPD ≥5 mm, CAL ≥3 mm and radiographic evidence of vertical osseous defects 3–6 mm	1	PI<br>GI<br>PPD<br>CAL
**Toker et al. (2012)**	GCF sample site	1	PI<br>GI<br>PPD<br>CAL
**Tomasi et al. (2004) (control)**	sites with PPD of ≥5 mm and BOP+, with an average of 22 sites/patient	1	PPD
**Türkoglu et al. (2016)**	full mouth (at six sites per tooth excluding third molars)	0	PI<br>BOP<br>PPD<br>CAL
**Vajawat et al. (2022) (control)**	split mouth design: sites randomly divided into test and control sites	0	PPD<br>CAL
**Varghese et al. (2020)**	full mouth	0	PI<br>GI<br>PPD<br>CAL
**Vijayakumar et al. (2020)**	full mouth: at six sites of all teeth	0	PI<br>GI<br>PPD<br>CAL
**Vohra et al. (2020) (SRP)**	full mouth: at six sites of all teeth	0	BOP<br>PPD<br>CAL
**Wan et al. (2009)**	full mouth (six sites of each tooth, excluding third molars)	0	BOP<br>PPD
	diseased sites (with initial PPD≥5 mm)	1	BOP<br>PPD
**Yilmaz et al. (2023)**	periodontitis sites	1	PPD<br>CAL
**Zuabi et al. (1999)**	full mouth (six sites of each tooth, excluding third molars)	0	PI<br>GI<br>PPD<br>CAL

Table S5.1. Studies included in the subset analysis; BOP, bleeding on probing, PI, plaque index, FMPS, full mouth plaque score; GI, gingival index, PPD, probing pocket depth; GCF, gingival crevicular fluid; SRP, scaling and root planning, RSD, root surface debridement; CP, chronic periodontitis

Δ PPD_0

A

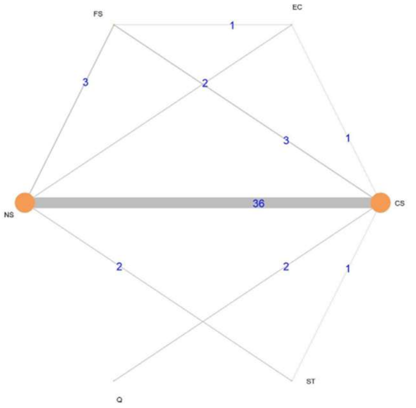

B

P-score (random)	
NS	0.8393
Q	0.7272
FS	0.7109
CS	0.5177
ST	0.1038
EC	0.1011

C

NS	.	-0.08 [-0.50; 0.35]	0.16 [0.02; 0.30]	1.20 [0.67; 1.73]	1.05 [0.51; 1.58]
0.04 [-0.56; 0.65]	Q	.	0.14 [-0.45; 0.74]	.	.
0.07 [-0.30; 0.44]	0.03 [-0.67; 0.73]	FS	0.08 [-0.36; 0.52]	.	0.34 [-0.44; 1.12]
0.19 [0.05; 0.32]	0.14 [-0.45; 0.74]	0.12 [-0.26; 0.49]	CS	-0.10 [-0.84; 0.64]	0.24 [-0.58; 1.06]
0.97 [0.48; 1.46]	0.93 [0.15; 1.70]	0.90 [0.29; 1.51]	0.78 [0.28; 1.28]	ST	.
0.98 [0.47; 1.48]	0.93 [0.15; 1.71]	0.91 [0.33; 1.48]	0.79 [0.28; 1.30]	0.01 [-0.69; 0.70]	EC

D

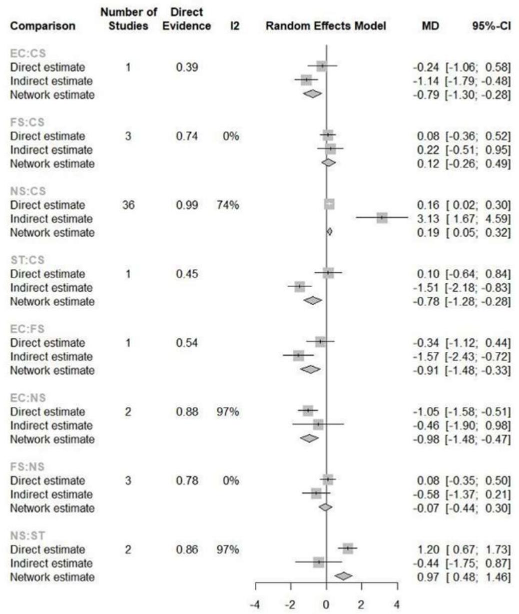

E

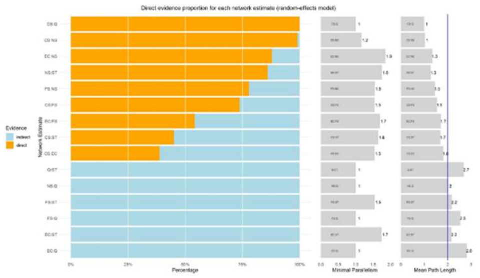

F

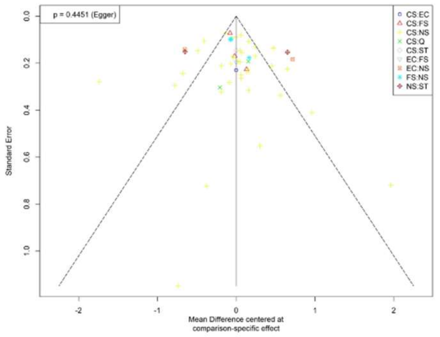

Figure S5.1. Network meta-analysis diagnostics and results for probing pocket depth (PPD) subset 0; A, Network geometry of the eligible comparisons in different nicotine-containing product user groups and non-smokers; B, Treatment ranking with P-scores under the random-effects model where higher values indicate larger outcome values, associated with favorable outcome between baseline and re-evaluation. C, League table shows the mean difference and 95% confidence interval for direct (upper right triangle) and network (lower left triangle) comparisons; D, Forest plot showing direct, indirect, and network estimates for each pairwise comparison to assess consistency-inconsistency; E, Evidence plot showing direct and indirect evidence proportion for each network estimate; F, Funnel plot to assess small-study effects; NS, non-smoker; CS, cigarette smoker; Q, quitter; FS, former smoker; EC, electronic cigarette; ST, smokeless tobacco

Δ PPD_1

A

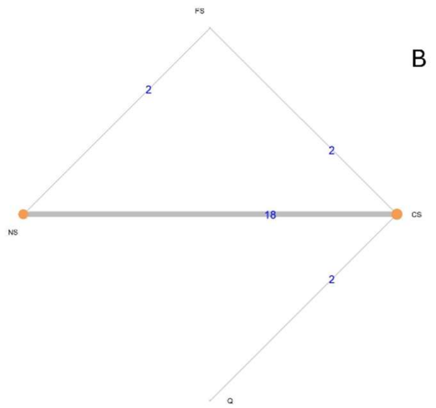

B

P-score (random)	
NS	0.7307
FS	0.6493
Q	0.5585
CS	0.0616

C

NS	-0.14 [-0.61; 0.33]	.	0.39 [0.20; 0.58]
0.04 [-0.38; 0.45]	FS	.	0.18 [-0.29; 0.65]
0.09 [-0.48; 0.66]	0.06 [-0.62; 0.73]	Q	0.30 [-0.24; 0.83]
0.39 [0.20; 0.58]	0.35 [-0.06; 0.77]	0.30 [-0.24; 0.83]	CS

D

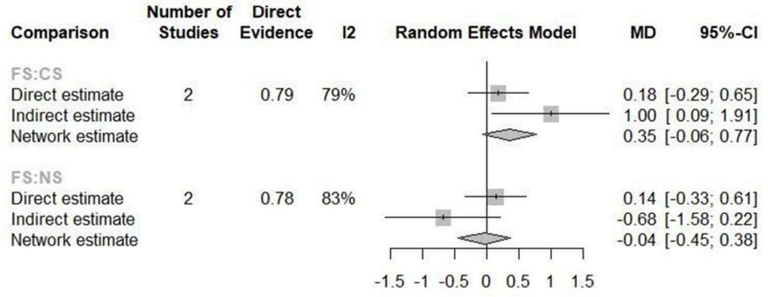

E

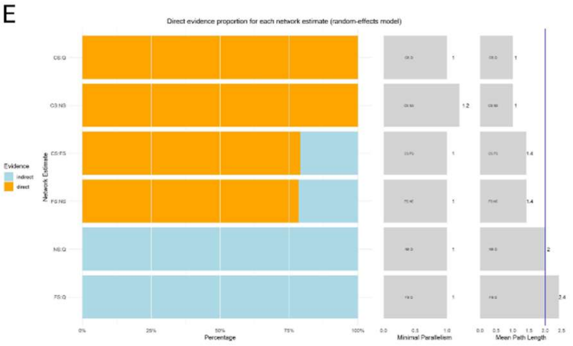

F

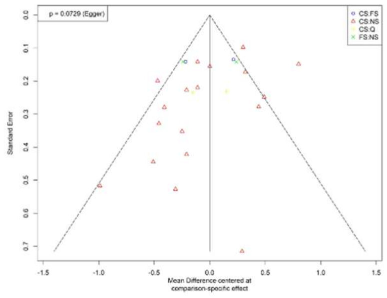

Figure S5.2. Network meta-analysis diagnostics and results for probing pocket depth (PPD) subset 1; A, Network geometry of the eligible comparisons in different nicotine-containing product user groups and non-smokers; B, Treatment ranking with P-scores under the random-effects model where higher values indicate larger outcome values, associated with favorable outcome between baseline and re-evaluation. C, League table shows the mean difference and 95% confidence interval for direct (upper right triangle) and network (lower left triangle) comparisons; D, Forest plot showing direct, indirect, and network estimates for each pairwise comparison to assess consistency-inconsistency; E, Evidence plot showing direct and indirect evidence proportion for each network estimate; F, Funnel plot to assess small-study effects; NS, non-smoker; CS, cigarette smoker; Q, quitter; FS, former smoker

Δ CAL_0

A

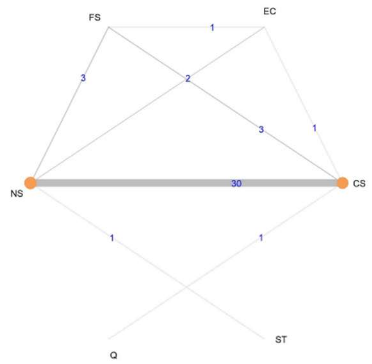

B

P-score (random)	
NS	0.7752
Q	0.7201
FS	0.5954
CS	0.4588
ST	0.2279
EC	0.2226

C

NS	-0.02 [-0.30; 0.25]	.	0.11 [-0.01; 0.23]	0.30 [-0.13; 0.73]	0.29 [-0.04; 0.61]
0.00 [-0.24; 0.25]	FS	.	0.03 [-0.27; 0.32]	.	0.30 [-0.28; 0.88]
0.03 [-0.73; 0.79]	0.02 [-0.77; 0.82]	Q	0.08 [-0.67; 0.83]	.	.
0.11 [-0.01; 0.23]	0.10 [-0.14; 0.35]	0.08 [-0.67; 0.83]	CS	.	0.33 [-0.31; 0.97]
0.30 [-0.13; 0.73]	0.30 [-0.20; 0.79]	0.27 [-0.60; 1.14]	0.19 [-0.25; 0.64]	ST	.
0.27 [-0.05; 0.59]	0.27 [-0.11; 0.65]	0.25 [-0.58; 1.07]	0.17 [-0.17; 0.50]	-0.03 [-0.56; 0.51]	EC

D

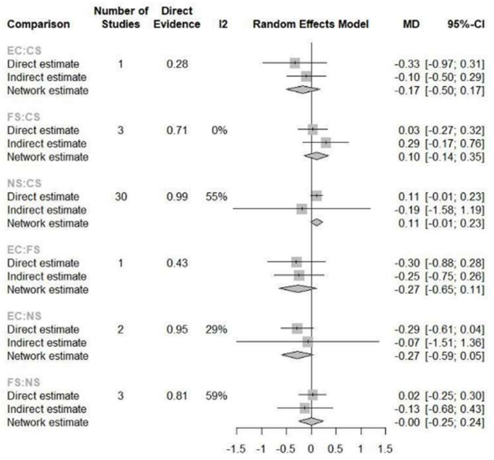

E

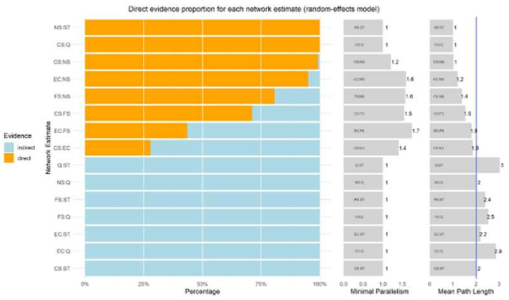

F

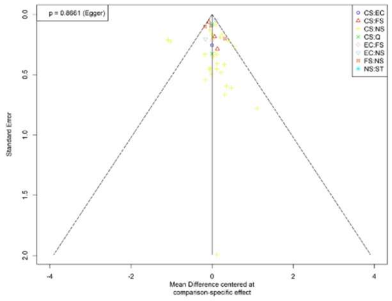

Figure S5.3. Network meta-analysis diagnostics and results for clinical attachment loss (CAL) subset 0; A, Network geometry of the eligible comparisons in different nicotine-containing product user groups and non-smokers; B, Treatment ranking with P-scores under the random-effects model where higher values indicate larger outcome values, associated with favorable outcome between baseline and re-evaluation. C, League table shows the mean difference and 95% confidence interval for direct (upper right triangle) and network (lower left triangle) comparisons; D, Forest plot showing direct, indirect, and network estimates for each pairwise comparison to assess consistency-inconsistency; E, Evidence plot showing direct and indirect evidence proportion for each network estimate; F, Funnel plot to assess small-study effects; NS, non-smoker; CS, cigarette smoker; Q, quitter; FS, former smoker; EC, electronic cigarette; ST, smokeless tobacco

Δ CAL_1

A

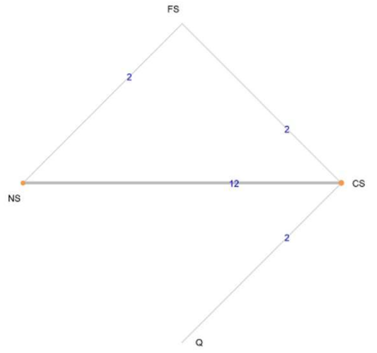

B

P-score (random)	
NS	0.7180
Q	0.6419
FS	0.3413
CS	0.2988

C

NS	.	0.27 [-0.16; 0.70]	0.15 [-0.07; 0.38]
-0.06 [-0.92; 0.80]	Q	.	0.21 [-0.62; 1.04]
0.16 [-0.22; 0.53]	0.22 [-0.69; 1.12]	FS	0.07 [-0.33; 0.47]
0.16 [-0.07; 0.38]	0.21 [-0.62; 1.04]	-0.00 [-0.37; 0.36]	CS

D

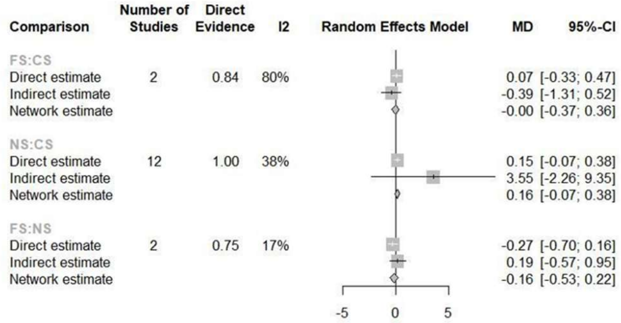

E

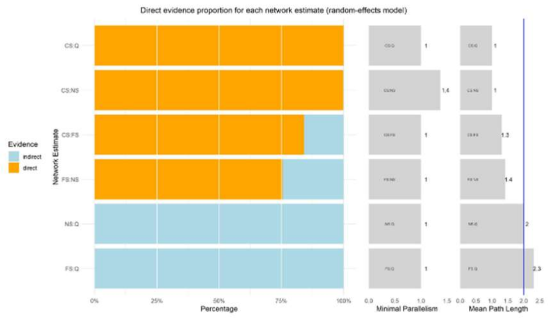

F

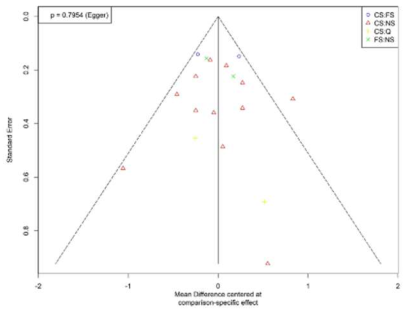

Figure S5.4. Network meta-analysis diagnostics and results for clinical attachment loss (CAL) subset 1; A, Network geometry of the eligible comparisons in different nicotine-containing product user groups and non-smokers; B, Treatment ranking with P-scores under the random-effects model where higher values indicate larger outcome values, associated with favorable outcome between baseline and re-evaluation. C, League table shows the mean difference and 95% confidence interval for direct (upper right triangle) and network (lower left triangle) comparisons; D, Forest plot showing direct, indirect, and network estimates for each pairwise comparison to assess consistency-inconsistency; E, Evidence plot showing direct and indirect evidence proportion for each network estimate; F, Funnel plot to assess small-study effects; NS, non-smoker; CS, cigarette smoker; Q, quitter; FS, former smoker

Δ BOP_0

A

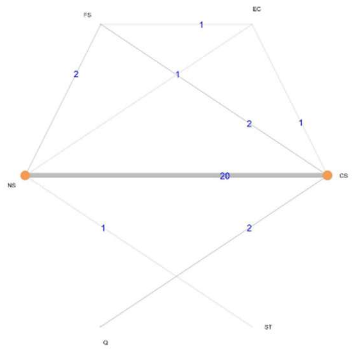

B

P-score (random)	
FS	0.8590
NS	0.7367
Q	0.5852
CS	0.3853
EC	0.3013
ST	0.1324

C

FS	0.56 [-21.26; 22.38]	.	20.69 [-1.38; 42.75]	16.00 [-15.60; 47.60]	.
6.25 [-13.07; 25.58]	NS	.	8.86 [1.92; 15.80]	13.60 [-17.65; 44.85]	25.40 [-3.90; 54.70]
9.81 [-20.65; 40.26]	3.56 [-20.97; 28.08]	Q	5.33 [-18.19; 28.85]	.	.
15.14 [-4.21; 34.49]	8.88 [1.94; 15.82]	5.33 [-18.19; 28.85]	CS	13.60 [-18.63; 45.83]	.
21.30 [-7.94; 50.55]	15.05 [-12.30; 42.40]	11.49 [-24.61; 47.60]	6.17 [-21.23; 33.56]	EC	.
31.65 [-3.44; 66.75]	25.40 [-3.90; 54.70]	21.84 [-16.36; 60.05]	16.52 [-13.59; 46.62]	10.35 [-29.73; 50.43]	ST

D

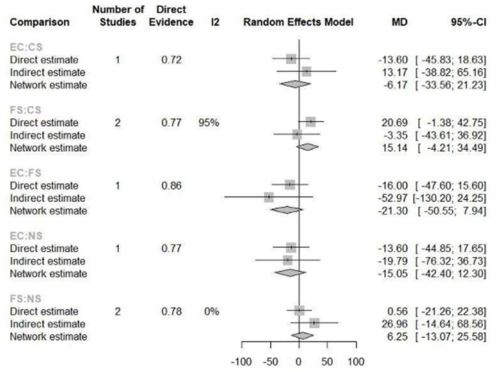

E

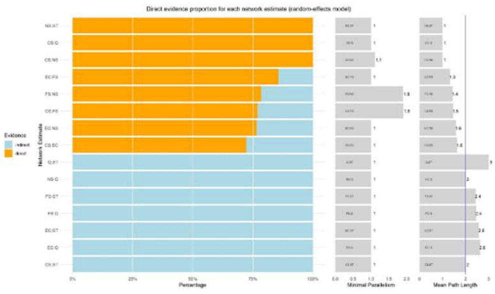

F

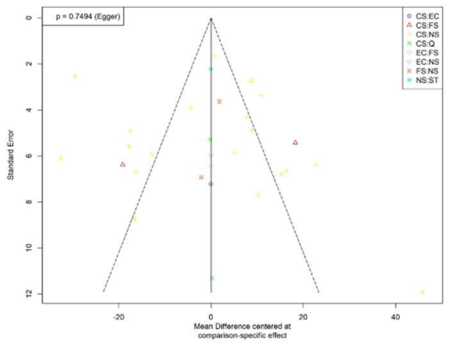

Figure S5.5. Network meta-analysis diagnostics and results for bleeding on probing (BOP) subset 0; A, Network geometry of the eligible comparisons in different nicotine-containing product user groups and non-smokers; B, Treatment ranking with P-scores under the random-effects model where higher values indicate larger outcome values, associated with favorable outcome between baseline and re-evaluation. C, League table shows the mean difference and 95% confidence interval for direct (upper right triangle) and network (lower left triangle) comparisons; D, Forest plot showing direct, indirect, and network estimates for each pairwise comparison to assess consistency-inconsistency; E, Evidence plot showing direct and indirect evidence proportion for each network estimate; F, Funnel plot to assess small-study effects; NS, non-smoker; CS, cigarette smoker; Q, quitter; FS, former smoker; EC, electronic cigarette; ST, smokeless tobacco

Δ BOP_1

A

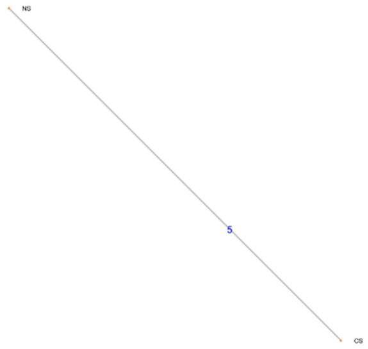

B

P-score (random)	
NS	0.5397
CS	0.4603

C

NS	0.47 [-8.85; 9.80]
0.47 [-8.85; 9.80]	CS

D

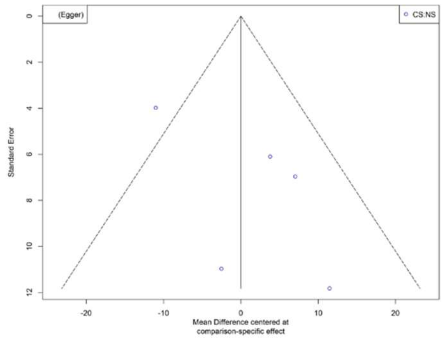

Figure S5.6. Network meta-analysis diagnostics and results for bleeding on probing (BOP) subset 1; A, Network geometry of the eligible comparisons in different nicotine-containing product user groups and non-smokers; B, Treatment ranking with P-scores under the random-effects model where higher values indicate larger outcome values, associated with favorable outcome between baseline and re-evaluation. C, League table shows the mean difference and 95% confidence interval for direct (upper right triangle) and network (lower left triangle) comparisons; D, Funnel plot to assess small-study effects; NS, non-smoker; CS, cigarette smoker

Δ PI_0

A

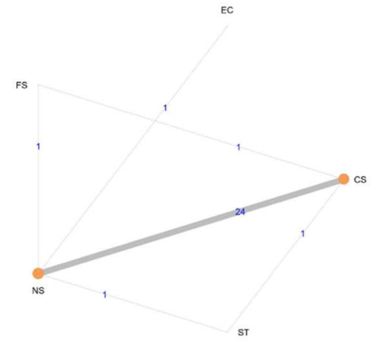

B

P-score (random)	
FS	0.8439
CS	0.6987
NS	0.4955
ST	0.4565
EC	0.0054

C

FS	0.15 [-0.44; 0.74]	0.28 [-0.34; 0.90]	.	.
0.18 [-0.34; 0.71]	CS	0.06 [-0.06; 0.19]	0.11 [-0.49; 0.71]	.
0.24 [-0.28; 0.77]	0.06 [-0.06; 0.19]	NS	0.13 [-0.46; 0.72]	0.92 [0.35; 1.49]
0.33 [-0.41; 1.07]	0.15 [-0.37; 0.68]	0.09 [-0.44; 0.62]	ST	.
1.16 [0.38; 1.94]	0.98 [0.39; 1.57]	0.92 [0.35; 1.49]	0.83 [0.05; 1.61]	EC

D

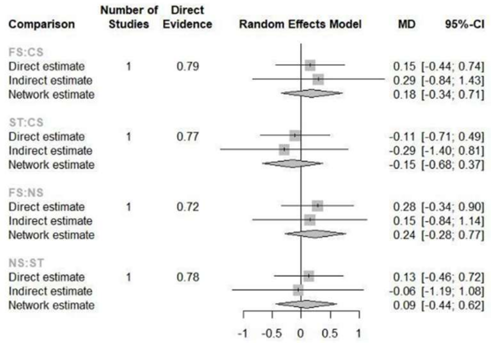

E

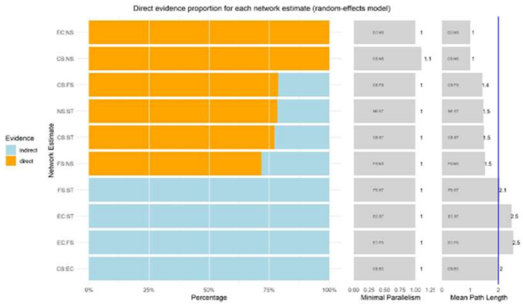

F

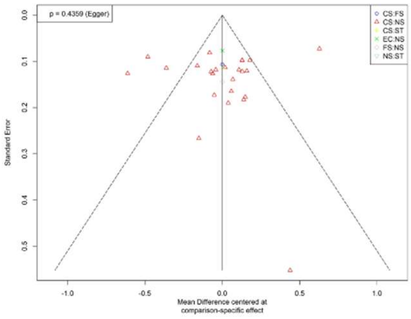

Figure S5.7. Network meta-analysis diagnostics and results for plaque index (PI) subset 0; A, Network geometry of the eligible comparisons in different nicotine-containing product user groups and non-smokers; B, Treatment ranking with P-scores under the random-effects model where higher values indicate larger outcome values, associated with favorable outcome between baseline and re-evaluation. C, League table shows the mean difference and 95% confidence interval for direct (upper right triangle) and network (lower left triangle) comparisons; D, Forest plot showing direct, indirect, and network estimates for each pairwise comparison to assess consistency-inconsistency; E, Evidence plot showing direct and indirect evidence proportion for each network estimate; F, Funnel plot to assess small-study effects; NS, non-smoker; CS, cigarette smoker; Q, quitter; FS, former smoker; EC, electronic cigarette

Δ GI_0

A

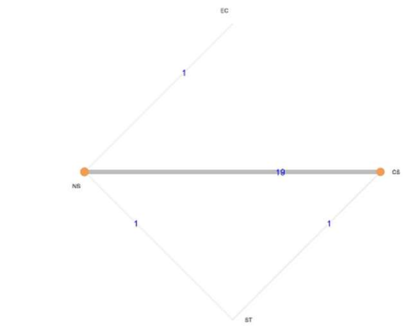

B

P-score (random)	
NS	0.9841
CS	0.5572
ST	0.4585
EC	0.0002

C

NS	0.32 [0.18; 0.47]	0.72 [0.13; 1.31]	1.67 [1.12; 2.22]
0.32 [0.18; 0.47]	CS	-0.15 [-0.73; 0.43]	.
0.44 [-0.08; 0.96]	0.12 [-0.40; 0.64]	ST	.
1.67 [1.12; 2.22]	1.35 [0.78; 1.92]	1.23 [0.47; 1.99]	EC

D

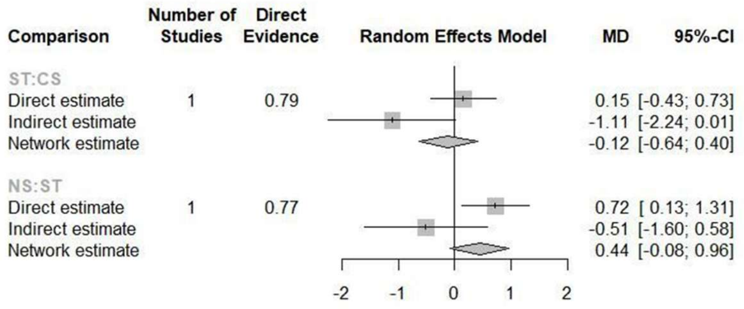

E

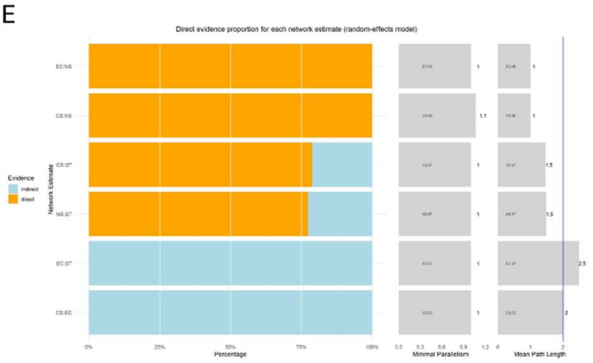

F

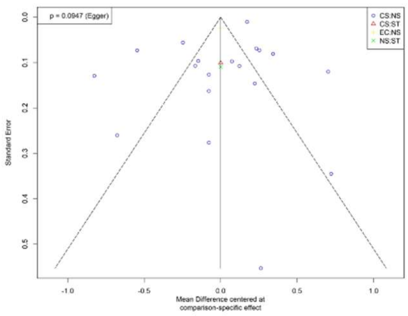

Figure S5.8. Network meta-analysis diagnostics and results for gingival index (GI) subset 0; A, Network geometry of the eligible comparisons in different nicotine-containing product user groups and non-smokers; B, Treatment ranking with P-scores under the random-effects model where higher values indicate larger outcome values, associated with favorable outcome between baseline and re-evaluation. C, League table shows the mean difference and 95% confidence interval for direct (upper right triangle) and network (lower left triangle) comparisons; D, Forest plot showing direct, indirect, and network estimates for each pairwise comparison to assess consistency-inconsistency; E, Evidence plot showing direct and indirect evidence proportion for each network estimate; F, Funnel plot to assess small-study effects; NS, non-smoker; CS, cigarette smoker; EC, electronic cigarette; ST, smokeless tobacco

Δ GI_1

A

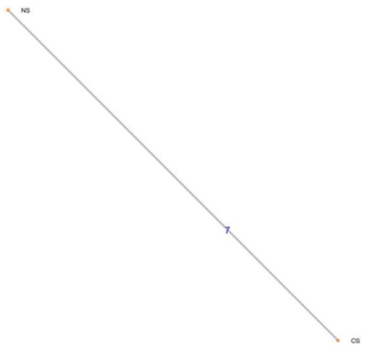

B

P-score (random)	
NS	0.9558
CS	0.0442

C

NS	0.25 [-0.04; 0.55]
0.25 [-0.04; 0.55]	CS

D

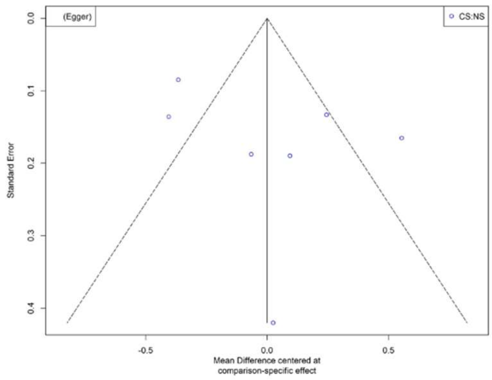

Figure S5.9. Network meta-analysis diagnostics and results for gingival index (GI) subset 1; A, Network geometry of the eligible comparisons in different nicotine-containing product user groups and non-smokers; B, Treatment ranking with P-scores under the random-effects model where higher values indicate larger outcome values, associated with favorable outcome between baseline and re-evaluation. C, League table shows the mean difference and 95% confidence interval for direct (upper right triangle) and network (lower left triangle) comparisons; D, Funnel plot to assess small-study effects; NS, non-smoker; CS, cigarette smoker
