# Supplementary material for: Impact of Traditional and Alternative Nicotine-Containing Product Use on Non-Surgical Periodontal Therapy: A Systematic Review and Network Meta-Analysis
Source: Dent J (Basel). 2026 Sep 22;14(10):616. doi: 10.3390/dj14100616 (PMC13703454; doi:10.3390/dj14100616)
Supplement: Supplementary file 1 [file dentistry-14-00616-s001.zip › Document S6 (Cinema).pdf]

1. Probing pocket depth

Comparison	No. of studies	Within study bias	Reporting bias	Indirectness	Imprecision	Heterogeneity	Incoherence	Confidence rating	Reason(s) for downgrading
**mixed evidence**									
CS vs EC	1	major concerns	low risk	no concerns	no concerns	no concerns	no concerns	**very low**	within study bias
CS vs FS	4	some concerns	low risk	no concerns	no concerns	no concerns	no concerns	**low**	within study bias
CS vs NS	48	some concerns	low risk	no concerns	no concerns	no concerns	no concerns	**moderate**	within study bias
CS vs Q	2	no concerns	low risk	no concerns	no concerns	no concerns	some concerns	**low**	incoherence
CS vs ST	1	some concerns	low risk	no concerns	no concerns	no concerns	no concerns	**low**	within study bias
EC vs FS	1	major concerns	low risk	no concerns	no concerns	no concerns	no concerns	**very low**	within study bias
EC vs NS	2	major concerns	low risk	no concerns	no concerns	no concerns	no concerns	**very low**	within study bias
FS vs NS	4	some concerns	low risk	no concerns	no concerns	no concerns	no concerns	**low**	within study bias
NS vs ST	2	some concerns	low risk	no concerns	no concerns	no concerns	no concerns	**low**	within study bias
**indirect evidence**									
EC vs Q	-	some concerns	low risk	some concerns	no concerns	no concerns	some concerns	**very low**	within study bias incoherence, indirectness
EC vs ST	-	some concerns	low risk	some concerns	no concerns	no concerns	some concerns	**very low**	within study bias incoherence, indirectness
FS vs Q	-	some concerns	low risk	some concerns	no concerns	no concerns	some concerns	**very low**	within study bias incoherence, indirectness
FS vs ST	-	some concerns	low risk	some concerns	no concerns	no concerns	some concerns	**very low**	within study bias incoherence, indirectness
NS vs Q	-	no concerns	low risk	some concerns	no concerns	no concerns	some concerns	**low**	incoherence, indirectness
Q vs ST	-	no concerns	low risk	some concerns	no concerns	no concerns	some concerns	**low**	incoherence, indirectness

Table S6.1: CINeMA for probing pocket depth CS, cigarette smokers; EC, electronic cigarette; FS, former smoker; Q, quitter; NS, non-smokers; ST, smokeless tobacco

2. Clinical attachment loss

Comparison	No. of studies	Within study bias	Reporting bias	Indirectness	Imprecision	Heterogeneity	Incoherence	Confidence rating	Reason(s) for downgrading
**mixed evidence**									
CS vs EC	1	major concerns	low risk	no concerns	no concerns	no concerns	no concerns	**very low**	within study bias
CS vs FS	4	some concerns	low risk	no concerns	no concerns	no concerns	no concerns	low	within study bias
CS vs NS	40	some concerns	low risk	no concerns	no concerns	no concerns	no concerns	**moderate**	within study bias
CS vs Q	2	no concerns	low risk	no concerns	no concerns	no concerns	no concerns	**moderate**	-
EC vs FS	1	major concerns	low risk	no concerns	no concerns	no concerns	no concerns	**very low**	within study bias
EC vs NS	2	major concerns	low risk	no concerns	no concerns	no concerns	no concerns	**very low**	within study bias
FS vs NS	4	some concerns	low risk	no concerns	no concerns	no concerns	no concerns	low	within study bias
NS vs ST	1	some concerns	low risk	no concerns	no concerns	no concerns	no concerns	low	within study bias
**indirect evidence**									
EC vs Q	-	some concerns	low risk	some concerns	no concerns	no concerns	no concerns	**very low**	within study bias, indirectness
EC vs ST	-	some concerns	low risk	some concerns	no concerns	no concerns	no concerns	**very low**	within study bias, indirectness
FS vs Q	-	some concerns	low risk	some concerns	no concerns	no concerns	no concerns	**very low**	within study bias, indirectness
FS vs ST	-	some concerns	low risk	some concerns	no concerns	no concerns	no concerns	**very low**	within study bias, indirectness
CS vs ST	-	some concerns	low risk	some concerns	no concerns	no concerns	no concerns	**very low**	within study bias, indirectness
NS vs Q	-	no concerns	low risk	some concerns	no concerns	no concerns	no concerns	low	indirectness
Q vs ST	-	no concerns	low risk	some concerns	no concerns	no concerns	no concerns	low	indirectness

Table S6.2: CINeMA for clinical attachment loss; CS, cigarette smokers; EC, electronic cigarette; FS, former smoker; Q, quitter; NS, non-smokers; ST, smokeless tobacco

3. Bleeding on probing

Comparison	No. of studies	Within study bias	Reporting bias	Indirectness	Imprecision	Heterogeneity	Incoherence	Confidence rating	Reason(s) for downgrading
**mixed evidence**									
CS vs EC	1	major concerns	low risk	no concerns	no concerns	no concerns	no concerns	**very low**	within study bias
CS vs FS	2	some concerns	low risk	no concerns	no concerns	no concerns	no concerns	low	within study bias
CS vs NS	22	some concerns	low risk	no concerns	no concerns	no concerns	some concerns	low	within study bias, incoherence
CS vs Q	2	no concerns	low risk	no concerns	no concerns	no concerns	some concerns	**moderate**	incoherence
EC vs FS	1	major concerns	low risk	no concerns	no concerns	some concerns	no concerns	**very low**	within study bias, heterogeneity
EC vs NS	1	major concerns	low risk	no concerns	no concerns	no concerns	no concerns	**very low**	within study bias
FS vs NS	2	some concerns	low risk	no concerns	no concerns	no concerns	no concerns	low	within study bias
NS vs ST	1	no concerns	low risk	no concerns	no concerns	no concerns	some concerns	low	incoherence
**indirect evidence**									
EC vs Q	-	some concerns	low risk	some concerns	no concerns	no concerns	some concerns	**very low**	within study bias, incoherence, indirectness
EC vs ST	-	some concerns	low risk	some concerns	no concerns	some concerns	some concerns	**very low**	within study bias, heterogeneity, incoherence, indirectness
FS vs Q	-	some concerns	low risk	some concerns	no concerns	some concerns	some concerns	**very low**	within study bias, heterogeneity, incoherence, indirectness
FS vs ST	-	some concerns	low risk	some concerns	no concerns	no concerns	some concerns	**very low**	within study bias, incoherence, indirectness
CS vs ST	-	no concerns	low risk	some concerns	no concerns	some concerns	some concerns	**very low**	heterogeneity, incoherence, indirectness
NS vs Q	-	no concerns	low risk	some concerns	no concerns	no concerns	some concerns	low	incoherence, indirectness
Q vs ST	-	no concerns	low risk	some concerns	no concerns	some concerns	some concerns	**very low**	heterogeneity, incoherence, indirectness

Table S6.3: CINeMA for bleeding on probing; CS, cigarette smokers; EC, electronic cigarette; FS, former smoker; Q, quitter; NS, non-smokers; ST, smokeless tobacco

4. Plaque index

Comparison	No. of studies	Within study bias	Reporting bias	Indirectness	Imprecision	Heterogeneity	Incoherence	Confidence rating	Reason(s) for downgrading
**mixed evidence**									
CS vs FS	1	some concerns	low risk	no concerns	no concerns	no concerns	no concerns	low	within study bias
CS vs NS	31	some concerns	low risk	no concerns	no concerns	no concerns	no concerns	**moderate**	within study bias
CS vs ST	1	some concerns	low risk	no concerns	no concerns	no concerns	no concerns	low	within study bias
EC vs NS	1	major concerns	low risk	no concerns	no concerns	no concerns	no concerns	**very low**	within study bias
FS vs NS	1	some concerns	low risk	no concerns	no concerns	no concerns	no concerns	low	within study bias
NS vs ST	1	some concerns	low risk	no concerns	no concerns	no concerns	no concerns	low	within study bias
**indirect evidence**									
CS vs EC	-	some concerns	low risk	some concerns	no concerns	no concerns	some concerns	**very low**	within study bias, incoherence, indirectness
EC vs ST	-	some concerns	low risk	some concerns	no concerns	no concerns	some concerns	**very low**	within study bias, incoherence, indirectness
EC vs FS	-	some concerns	low risk	some concerns	no concerns	no concerns	some concerns	**very low**	within study bias, incoherence, indirectness
FS vs ST	-	some concerns	low risk	some concerns	no concerns	no concerns	some concerns	**very low**	within study bias, incoherence, indirectness

Table S6.4: CINeMA for plaque index; CS, cigarette smokers; EC, electronic cigarette; FS, former smoker; NS, non-smokers; ST, smokeless tobacco

5. Gingival index

Comparison	No. of studies	Within study bias	Reporting bias	Indirectness	Imprecision	Heterogeneity	Incoherence	Confidence rating	Reason(s) for downgrading
**mixed evidence**									
CS vs NS	5	some concerns	low risk	no concerns	no concerns	no concerns	no concerns	low	within study bias
CS vs ST	1	some concerns	low risk	no concerns	no concerns	no concerns	no concerns	low	within study bias
EC vs NS	1	major concerns	low risk	no concerns	no concerns	some concerns	no concerns	**very low**	within study bias heterogeneity
NS vs ST	1	some concerns	low risk	no concerns	no concerns	no concerns	no concerns	low	within study bias
**indirect evidence**									
CS vs EC	-	some concerns	low risk	some concerns	no concerns	some concerns	no concerns	**very low**	within study bias heterogeneity, indirectness
EC vs ST	-	some concerns	low risk	some concerns	no concerns	some concerns	no concerns	**very low**	within study bias heterogeneity, indirectness

Table S6.5: CINeMA for gingival index; CS, cigarette smokers; EC, electronic cigarette; NS, non-smokers; ST, smokeless tobacco

6. Full mouth plaque score

Comparison	No. of studies	Within study bias	Reporting bias	Indirectness	Imprecision	Heterogeneity	Incoherence	Confidence rating	Reason(s) for downgrading
**mixed evidence**									
CS vs EC	1	major concerns	low risk	no concerns	no concerns	no concerns	no concerns	**very low**	within study bias
CS vs FS	2	some concerns	low risk	no concerns	no concerns	no concerns	no concerns	low	within study bias
CS vs NS	12	some concerns	low risk	no concerns	no concerns	no concerns	no concerns	low	within study bias
CS vs Q	2	no concerns	low risk	no concerns	no concerns	no concerns	no concerns	**moderate**	-
EC vs FS	1	major concerns	low risk	no concerns	no concerns	no concerns	no concerns	**very low**	within study bias
EC vs NS	1	major concerns	low risk	no concerns	no concerns	no concerns	no concerns	**very low**	within study bias
FS vs NS	2	some concerns	low risk	no concerns	no concerns	no concerns	no concerns	low	within study bias
NS vs ST	1	no concerns	low risk	no concerns	no concerns	no concerns	no concerns	**moderate**	-
**indirect evidence**									
EC vs Q	-	some concerns	low risk	some concerns	no concerns	no concerns	no concerns	**moderate**	within study bias, indirectness
EC vs ST	-	some concerns	low risk	some concerns	no concerns	no concerns	no concerns	**moderate**	within study bias, indirectness
FS vs Q	-	some concerns	low risk	some concerns	no concerns	no concerns	no concerns	**moderate**	within study bias, indirectness
FS vs ST	-	some concerns	low risk	some concerns	no concerns	no concerns	no concerns	**moderate**	within study bias, indirectness
CS vs ST	-	no concerns	low risk	some concerns	no concerns	no concerns	no concerns	low	indirectness
NS vs Q	-	no concerns	low risk	some concerns	no concerns	no concerns	no concerns	low	indirectness
Q vs ST	-	no concerns	low risk	some concerns	no concerns	no concerns	no concerns	low	indirectness

Table S6.6: CINeMA for full mouth plaque score; CS, cigarette smokers; EC, electronic cigarette; FS, former smoker; Q, quitter; NS, non-smokers; ST, smokeless tobacco

7. Gingival crevicular fluid volume

Comparison	No. of studies	Within study bias	Reporting bias	Indirectness	Imprecision	Heterogeneity	Incoherence	Confidence rating	Reason(s) for downgrading
**mixed evidence**									
CS vs NS	5	some concerns	low risk	no concerns	no concerns	no concerns	no concerns	low	within study bias
CS vs ST	1	some concerns	low risk	no concerns	no concerns	no concerns	no concerns	low	within study bias
EC vs NS	1	major concerns	low risk	no concerns	no concerns	some concerns	no concerns	very low	within study bias heterogeneity
NS vs ST	1	some concerns	low risk	no concerns	no concerns	no concerns	no concerns	low	within study bias
**indirect evidence**									
CS vs EC	-	some concerns	low risk	some concerns	no concerns	major concerns	no concerns	very low	within study bias heterogeneity, indirectness
EC vs ST	-	some concerns	low risk	some concerns	no concerns	major concerns	no concerns	very low	within study bias heterogeneity, indirectness

Table S6.7: CINeMA for gingival crevicular fluid volume CS, cigarette smokers; EC, electronic cigarette; NS, non-smokers; ST, smokeless tobacco
