# Supplementary material for: Impact of Traditional and Alternative Nicotine-Containing Product Use on Non-Surgical Periodontal Therapy: A Systematic Review and Network Meta-Analysis
Source: Dent J (Basel). 2026 Sep 22;14(10):616. doi: 10.3390/dj14100616 (PMC13703454; doi:10.3390/dj14100616)
Supplement: Supplementary file 1 [file dentistry-14-00616-s001.zip › Document S7 (High risk sensitivity analysis).pdf]

Results of the sensitivity analyses excluding high risk studies

Sensitivity analysis was conducted excluding high risk studies. All studies including electronic cigarette users were assessed as high risk of bias; therefore, this group was excluded from the sensitivity analysis.

Δ PPD

A

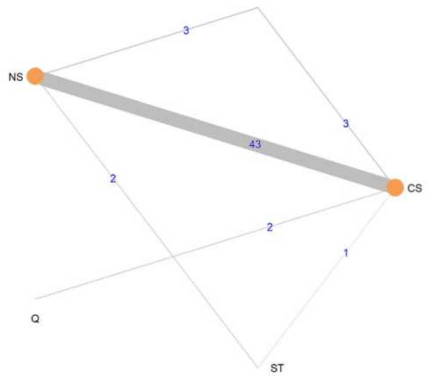

B

P-score (random)	
NS	0.7469
FS	0.7461
Q	0.6822
CS	0.3231
ST	0.0018

C

NS	-0.19 [-0.61; 0.24]	.	0.23 [0.11; 0.36]	1.20 [0.68; 1.72]
-0.02 [-0.39; 0.36]	FS	.	0.08 [-0.35; 0.51]	.
0.02 [-0.55; 0.58]	0.03 [-0.63; 0.70]	Q	0.23 [-0.32; 0.78]	.
0.24 [0.12; 0.37]	0.26 [-0.11; 0.64]	0.23 [-0.32; 0.78]	CS	-0.10 [-0.84; 0.64]
0.99 [0.50; 1.47]	1.00 [0.39; 1.61]	0.97 [0.23; 1.71]	0.74 [0.25; 1.23]	ST

D

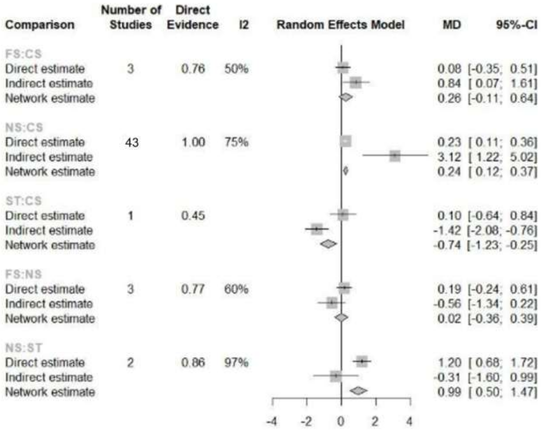

E

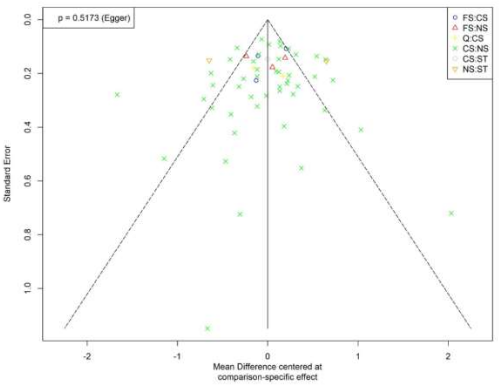

Figure S7.1. A, Network geometry of the eligible comparisons of change in probing pocket depth (PPD) in different nicotine-containing product user groups and non-smokers; B, Treatment ranking with P-scores under the random-effects model where higher values indicate larger outcome values, associated with favorable outcome between baseline and re-evaluation. C, League table shows the mean difference and 95% confidence interval for direct (upper right triangle) and network (lower left triangle) comparisons; D, Forest plot showing direct, indirect, and network estimates for each pairwise comparison to assess consistency-inconsistency; E, Funnel plot to assess small-study effects NS, non-smokers; Q, quitters; FS, former smokers; CS, cigarette smokers; ST, smokeless tobacco.

Δ CAL

A

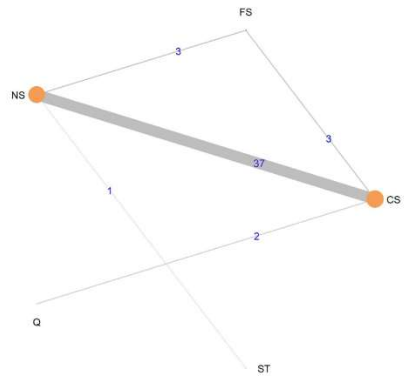

B

P-score (random)	
NS	0.7801
Q	0.5814
FS	0.5398
CS	0.3788
ST	0.2199

C

NS	.	0.06 [-0.28; 0.40]	0.14 [0.02; 0.26]	0.30 [-0.18; 0.78]
0.03 [-0.76; 0.83]	Q	.	0.10 [-0.68; 0.89]	.
0.08 [-0.21; 0.38]	0.05 [-0.79; 0.88]	FS	-0.00 [-0.34; 0.33]	.
0.14 [0.02; 0.26]	0.10 [-0.68; 0.89]	0.05 [-0.24; 0.35]	CS	.
0.30 [-0.18; 0.78]	0.27 [-0.66; 1.19]	0.22 [-0.35; 0.78]	0.16 [-0.33; 0.66]	ST

D

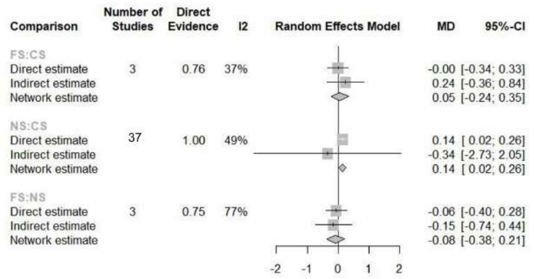

E

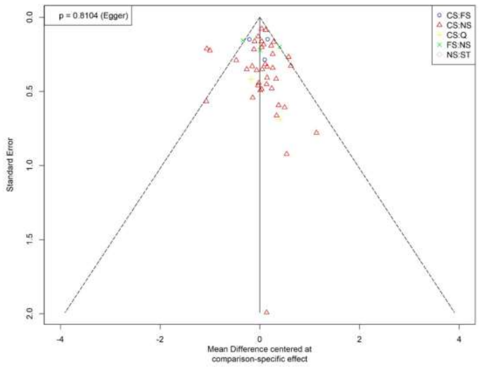

Figure S7.2. A, Network geometry of the eligible comparisons of change in clinical attachment loss (CAL) in different nicotine-containing product user groups and non-smokers; B, Treatment ranking with P-scores under the random-effects model where higher values indicate larger outcome values, associated with favorable outcome between baseline and re-evaluation. C, League table shows the mean difference and 95% confidence interval for direct (upper right triangle) and network (lower left triangle) comparisons; D, Forest plot showing direct, indirect, and network estimates for each pairwise comparison to assess consistency-inconsistency; E, Funnel plot to assess small-study effects NS, non-smokers; Q, quitters; FS, former smokers; CS, cigarette smokers; ST, smokeless tobacco.

Δ BOP

A

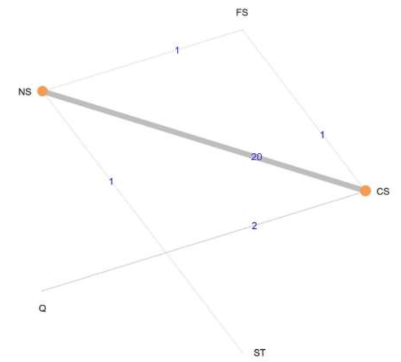

B

P-score (random)	
FS	0.9075
NS	0.6974
Q	0.4969
CS	0.2987
ST	0.0995

C

FS	-1.56 [-33.44; 30.32]	.	39.81 [8.36; 71.26]	.
14.08 [-13.69; 41.84]	NS	.	10.96 [4.12; 17.79]	25.40 [-3.78; 54.58]
19.70 [-16.62; 56.02]	5.63 [-18.79; 30.05]	Q	5.33 [-18.12; 28.77]	.
25.03 [-2.71; 52.77]	10.96 [4.12; 17.79]	5.33 [-18.12; 28.77]	CS	.
39.48 [-0.80; 79.75]	25.40 [-3.78; 54.58]	19.77 [-18.27; 57.82]	14.44 [-15.52; 44.41]	ST

D

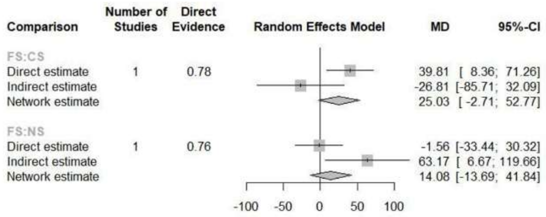

E

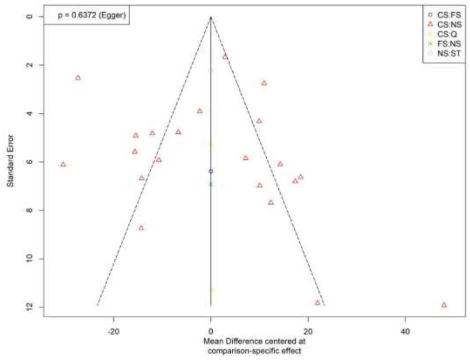

Figure S7.3. A, Network geometry of the eligible comparisons of change in bleeding on probing (BOP) in different nicotine-containing product user groups and non-smokers; B, Treatment ranking with P-scores under the random-effects model where higher values indicate larger outcome values, associated with favorable outcome between baseline and re-evaluation. C, League table shows the mean difference and 95% confidence interval for direct (upper right triangle) and network (lower left triangle) comparisons; D, Forest plot showing direct, indirect, and network estimates for each pairwise comparison to assess consistency-inconsistency; E, Funnel plot to assess small-study effects NS, non-smokers; Q, quitters; FS, former smokers; CS, cigarette smokers; ST, smokeless tobacco.

Δ PI

A

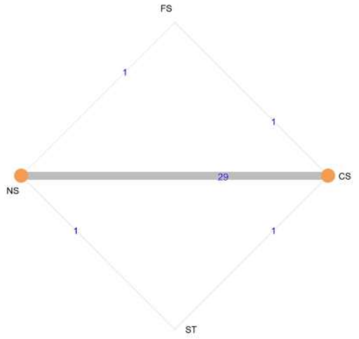

B

P-score (random)	
FS	0.8025
CS	0.6036
NS	0.3208
ST	0.2731

C

FS	0.15 [-0.42; 0.72]	0.28 [-0.32; 0.88]	.
0.18 [-0.32; 0.68]	CS	0.06 [-0.05; 0.17]	0.11 [-0.46; 0.68]
0.24 [-0.26; 0.74]	0.06 [-0.05; 0.17]	NS	0.13 [-0.44; 0.70]
0.33 [-0.38; 1.04]	0.15 [-0.35; 0.65]	0.09 [-0.41; 0.59]	ST

D

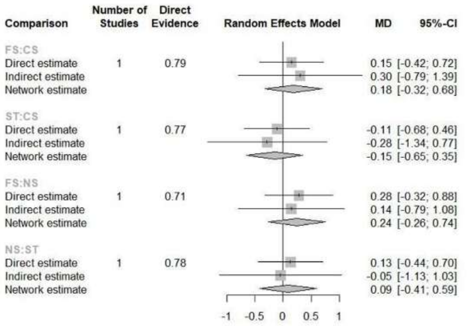

E

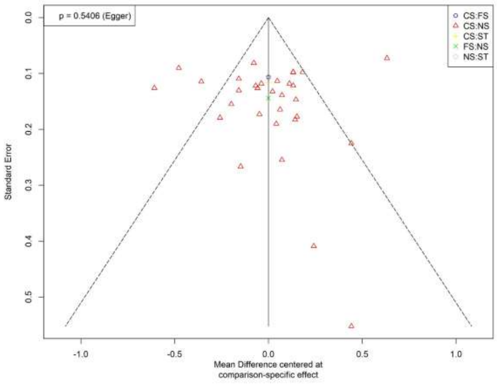

Figure S7.4. A, Network geometry of the eligible comparisons of change in plaque index (PI) in different nicotine-containing product user groups and non-smokers; B, Treatment ranking with P-scores under the random-effects model where higher values indicate larger outcome values, associated with favorable outcome between baseline and re-evaluation. C, League table shows the mean difference and 95% confidence interval for direct (upper right triangle) and network (lower left triangle) comparisons; D, Forest plot showing direct, indirect, and network estimates for each pairwise comparison to assess consistency-inconsistency; E, Funnel plot to assess small-study effects NS, non-smokers; FS, former smokers; CS, cigarette smokers; ST, smokeless tobacco.

Δ GI

A

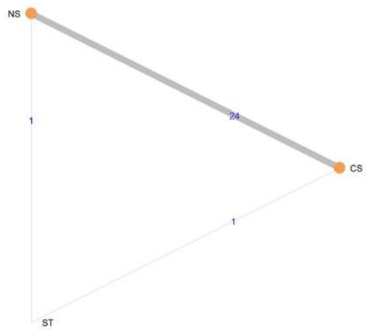

P-score (random)	
NS	0.9742
CS	0.3454
ST	0.1803

C

NS	0.30 [0.17; 0.42]	0.72 [0.14; 1.30]
0.30 [0.17; 0.42]	CS	-0.15 [-0.73; 0.43]
0.43 [-0.09; 0.94]	0.13 [-0.38; 0.64]	ST

D

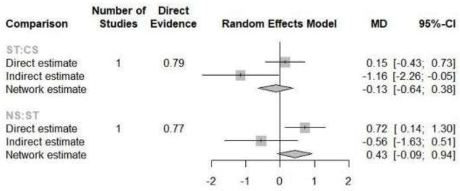

E

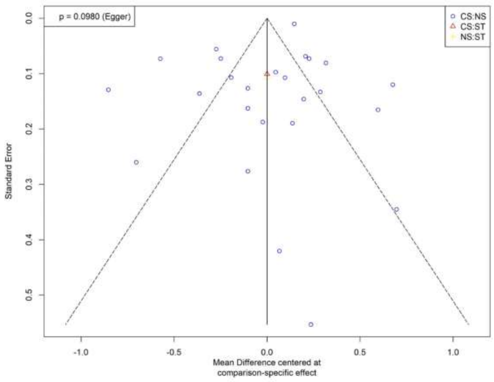

Figure S7.5. A, Network geometry of the eligible comparisons of change in gingival index (GI) in different nicotine-containing product user groups and non-smokers; B, Treatment ranking with P-scores under the random-effects model where higher values indicate larger outcome values, associated with favorable outcome between baseline and re-evaluation. C, League table shows the mean difference and 95% confidence interval for direct (upper right triangle) and network (lower left triangle) comparisons; D, Forest plot showing direct, indirect, and network estimates for each pairwise comparison to assess consistency-inconsistency; E, Funnel plot to assess small-study effects NS, non-smokers; CS, cigarette smokers; ST, smokeless tobacco.

Δ FMPS

A

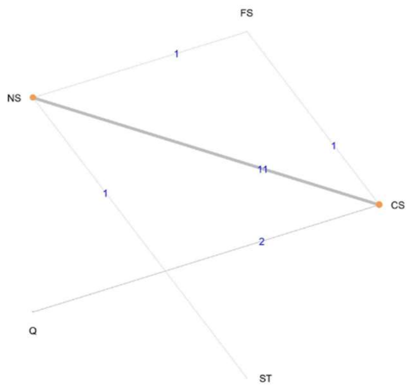

P-score (random)	
FS	0.7290
Q	0.7063
NS	0.5640
CS	0.3551
ST	0.1456

C

FS	.	1.18 [-22.27; 24.63]	13.60 [-9.92; 37.12]	.
0.75 [-27.82; 29.32]	Q	.	8.04 [-11.70; 27.78]	.
5.93 [-14.72; 26.58]	5.18 [-15.80; 26.15]	NS	2.86 [-4.23; 9.95]	12.00 [-8.90; 32.90]
8.79 [-11.86; 29.45]	8.04 [-11.70; 27.78]	2.86 [-4.23; 9.95]	CS	.
17.93 [-11.45; 47.31]	17.18 [-12.43; 46.79]	12.00 [-8.90; 32.90]	9.14 [-12.93; 31.21]	ST

D

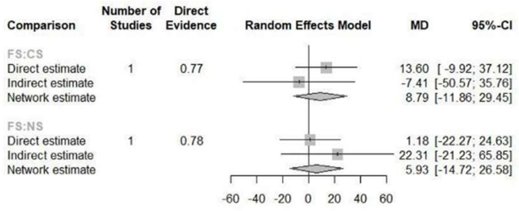

E

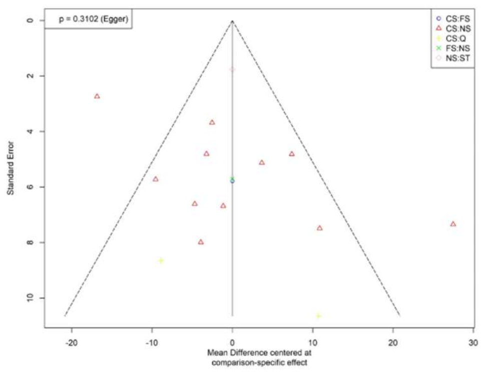

Figure S7.6. A, Network geometry of the eligible comparisons of change in full mouth plaque score (FMPS) in different nicotine-containing product user groups and non-smokers; B, Treatment ranking with P-scores under the random-effects model where higher values indicate larger outcome values, associated with favorable outcome between baseline and re-evaluation. C, League table shows the mean difference and 95% confidence interval for direct (upper right triangle) and network (lower left triangle) comparisons; D, Forest plot showing direct, indirect, and network estimates for each pairwise comparison to assess consistency-inconsistency; E, Funnel plot to assess small-study effects NS, non-smokers; Q, quitters; FS, former smokers; CS, cigarette smokers; ST, smokeless tobacco.

Δ GCF

A

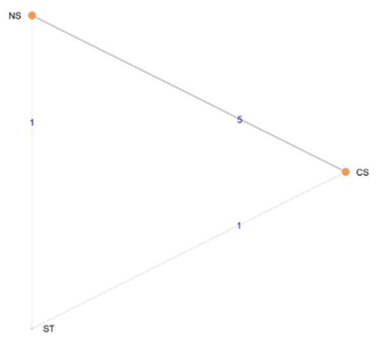

P-score (random)	
NS	0.8790
ST	0.4723
CS	0.1487

C

NS	0.02 [-0.15; 0.19]	0.11 [0.01; 0.21]
0.06 [-0.10; 0.22]	ST	0.00 [-0.17; 0.17]
0.11 [0.01; 0.21]	0.05 [-0.11; 0.20]	CS

D

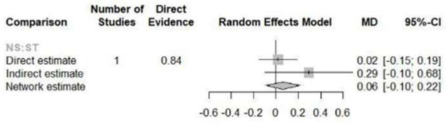

E

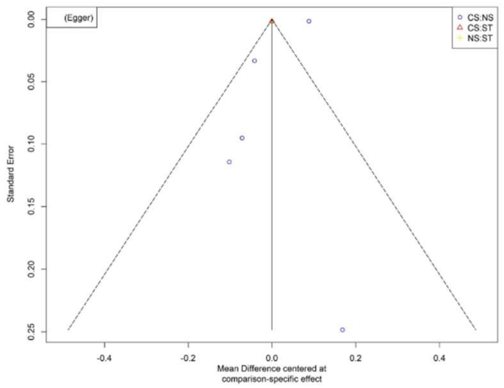

Figure S7.7. A, Network geometry of the eligible comparisons of change in gingival crevicular fluid (GCF) volume in different nicotine-containing product user groups and non-smokers; B, Treatment ranking with P-scores under the random-effects model where higher values indicate larger outcome values, associated with favorable outcome between baseline and re-evaluation. C, League table shows the mean difference and 95% confidence interval for direct (upper right triangle) and network (lower left triangle) comparisons; D, Forest plot showing direct, indirect, and network estimates for each pairwise comparison to assess consistency-inconsistency; E, Funnel plot to assess small-study effects NS, non-smokers; CS, cigarette smokers; ST, smokeless tobacco.
