# Supplementary material for: Impact of Traditional and Alternative Nicotine-Containing Product Use on Non-Surgical Periodontal Therapy: A Systematic Review and Network Meta-Analysis
Source: Dent J (Basel). 2026 Sep 22;14(10):616. doi: 10.3390/dj14100616 (PMC13703454; doi:10.3390/dj14100616)
Supplement: Supplementary file 1 [file dentistry-14-00616-s001.zip › Document S8 (RCT sensitivity analysis).pdf]

Results of the sensitivity analyses including only randomised clinical trials

A sensitivity analysis was conducted including only randomized clinical trials (RCTs). The number of studies contributing to each network is summarized in Supplementary Table 8.1. As no RCTs included quitters or electronic cigarette users, these exposure groups could not be included in the sensitivity analysis. Gingival crevicular fluid (GCF) volume was assessed in only one study; therefore, a network meta-analysis could not be performed for this outcome. Furthermore, only one RCT included former smokers (FS) and only one included smokeless tobacco (ST) users, resulting in weak or poorly connected networks for these exposure categories. The Plaque index (PI) and gingival index (GI) analyses included only non-smokers (NS) and cigarette smokers (CS), as no RCTs were available for the other exposure groups.

**Outcome**	**Number of studies in each exposure group**			
	**NS**	**CS**	**FS**	**ST**
**PPD**	12	12	1	1
**CAL**	11	11	1	1
**BOP**	6	6	0	1
**PI**	5	5	0	0
**GI**	5	5	0	0
**FMPS**	5	5	0	1
**GCF**	1	1	0	0

Table S8.1. Number of studies included in the sensitivity analysis restricted to randomized clinical trials, by outcome and exposure group. NS, non-smokers; CS, cigarette smokers; FS, former smokers; ST, smokeless tobacco users; PPD, probing pocket depth; CAL, clinical attachment level; BOP, bleeding on probing; PI, plaque index; GI, gingival index; FMPS, full-mouth plaque score; GCF, gingival crevicular fluid volume.

The results of each network analysis are presented in the following figures for each clinical outcome. The figures summarize the evidence networks, treatment rankings, pairwise comparisons, and assessments of consistency and small-study effects, where applicable.

Δ PPD

A

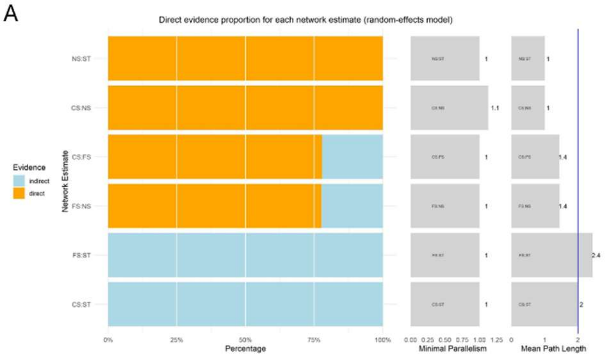

B

P-score (random)	
NS	0.8509
FS	0.7241
CS	0.4010
ST	0.0240

C

NS	-0.38 [-1.74; 0.98]	0.55 [0.09; 1.01]	1.85 [0.48; 3.22]
0.10 [-1.10; 1.30]	FS	-0.03 [-1.39; 1.33]	.
0.55 [0.09; 1.01]	0.45 [-0.75; 1.65]	CS	.
1.85 [0.48; 3.22]	1.75 [-0.07; 3.56]	1.30 [-0.14; 2.74]	ST

D

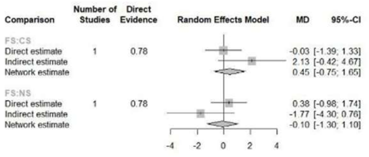

E

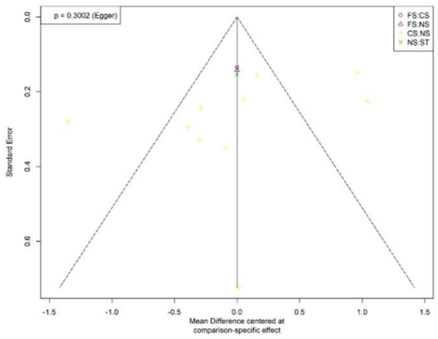

Figure S8.1. A, Evidence plot showing direct and indirect comparisons of change in probing pocket depth (PPD) in different nicotine-containing product user groups and non-smokers; B, Treatment ranking with P-scores under the random-effects model where higher values indicate larger outcome values, associated with favorable outcome between baseline and re-evaluation. C, League table shows the mean difference and 95% confidence interval for direct (upper right triangle) and network (lower left triangle) comparisons; D, Forest plot showing direct, indirect, and network estimates for each pairwise comparison to assess consistency-inconsistency; E, Funnel plot to assess small-study effects NS, non-smokers; FS, former smokers; CS, cigarette smokers; ST, smokeless tobacco.

Δ CAL

A

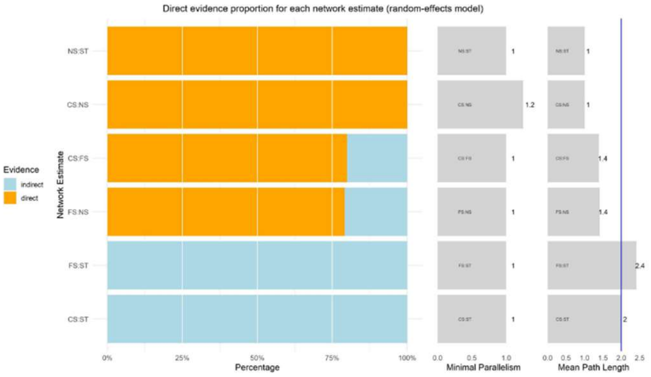

B

P-score (random)	
NS	0.8983
ST	0.4920
CS	0.3230
FS	0.2867

C

NS	0.30 [-0.44; 1.04]	0.42 [0.07; 0.77]	0.40 [-0.38; 1.18]
0.30 [-0.44; 1.04]	ST	.	.
0.42 [0.07; 0.77]	0.12 [-0.69; 0.94]	CS	0.16 [-0.62; 0.94]
0.49 [-0.20; 1.19]	0.19 [-0.82; 1.21]	0.07 [-0.63; 0.76]	FS

D

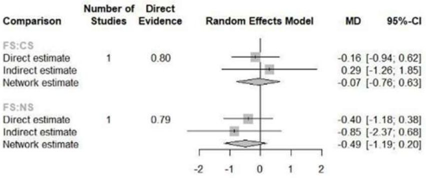

E

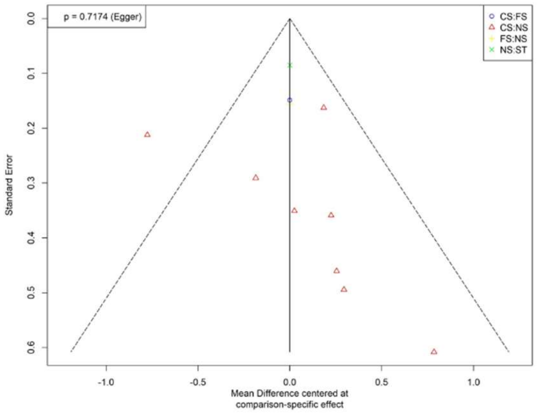

Figure S8.2. A, Evidence plot showing direct and indirect comparisons of change in clinical attachment loss (CAL) in different nicotine-containing product user groups and non-smokers; B, Treatment ranking with P-scores under the random-effects model where higher values indicate larger outcome values, associated with favorable outcome between baseline and re-evaluation. C, League table shows the mean difference and 95% confidence interval for direct (upper right triangle) and network (lower left triangle) comparisons; D, Forest plot showing direct, indirect, and network estimates for each pairwise comparison to assess consistency-inconsistency; E, Funnel plot to assess small-study effects NS, non-smokers; FS, former smokers; CS, cigarette smokers; ST, smokeless tobacco.

Δ BOP

A

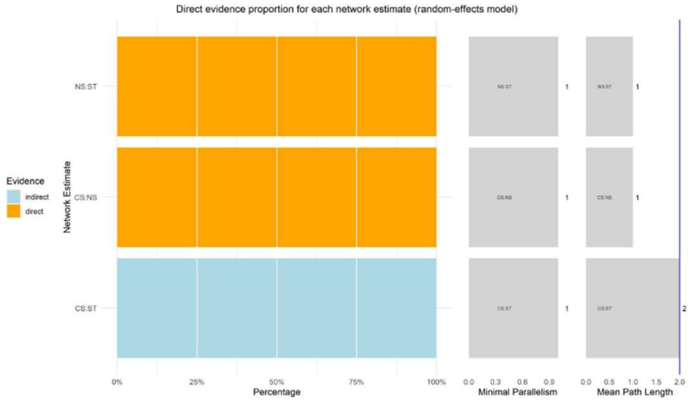

B

P-score (random)	
NS	0.9999
ST	0.3812
CS	0.1189

C

NS	25.40 [11.62; 39.18]	31.39 [22.39; 40.39]
25.40 [11.62; 39.18]	ST	.
31.39 [22.39; 40.39]	5.99 [-10.47; 22.45]	CS

D

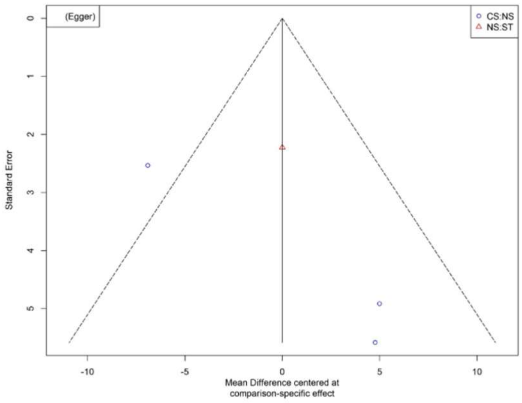

Figure S8.3. A, Evidence plot showing direct and indirect comparisons of change in bleeding on probing (BOP) in different nicotine-containing product user groups and non-smokers; B, Treatment ranking with P-scores under the random-effects model where higher values indicate larger outcome values, associated with favorable outcome between baseline and re-evaluation. C, League table shows the mean difference and 95% confidence interval for direct (upper right triangle) and network (lower left triangle) comparisons; D, Funnel plot to assess small-study effects NS, non-smokers; CS, cigarette smokers; ST, smokeless tobacco.

Δ PI

A

P-score (random)	
NS	0.5501
CS	0.4499

B

NS	0.01 [-0.16; 0.18]
0.01 [-0.16; 0.18]	CS

C

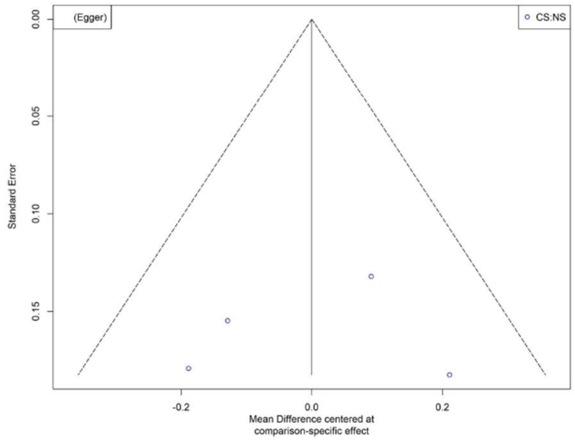

Figure S8.4. A, Treatment ranking with P-scores under the random-effects model where higher values indicate larger outcome values, associated with favorable outcome between baseline and re-evaluation. B, League table shows the mean difference and 95% confidence interval for direct (upper right triangle) and network (lower left triangle) comparisons; C, Funnel plot to assess small-study effects NS, non-smokers; CS, cigarette smokers;

Δ GI

A

P-score (random)	
NS	0.9576
CS	0.0424

B

NS	0.27 [-0.04; 0.58]
0.27 [-0.04; 0.58]	CS

C

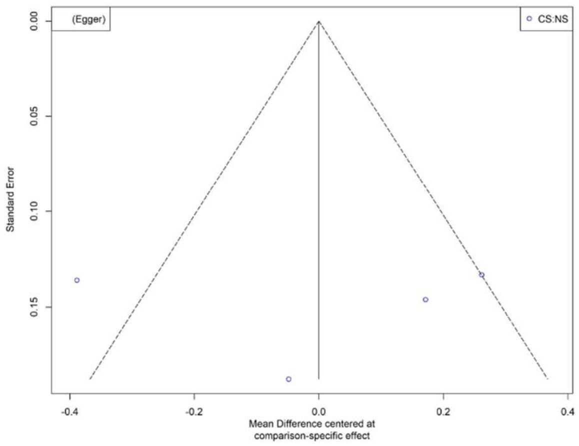

Figure S8.5. A, Treatment ranking with P-scores under the random-effects model where higher values indicate larger outcome values, associated with favorable outcome between baseline and re-evaluation. B, League table shows the mean difference and 95% confidence interval for direct (upper right triangle) and network (lower left triangle) comparisons; C, Funnel plot to assess small-study effects NS, non-smokers; CS, cigarette smokers;

Δ FMPS

A

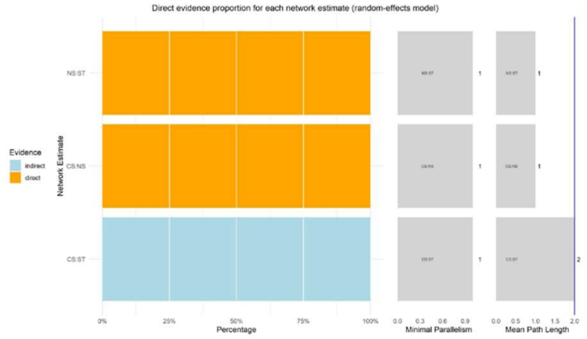

B

P-score (random)	
NS	0.9508
CS	0.2841
ST	0.2651

C

NS	10.79 [0.49; 21.10]	12.00 [-4.61; 28.61]
10.79 [0.49; 21.10]	CS	.
12.00 [-4.61; 28.61]	1.21 [-18.34; 20.75]	ST

D

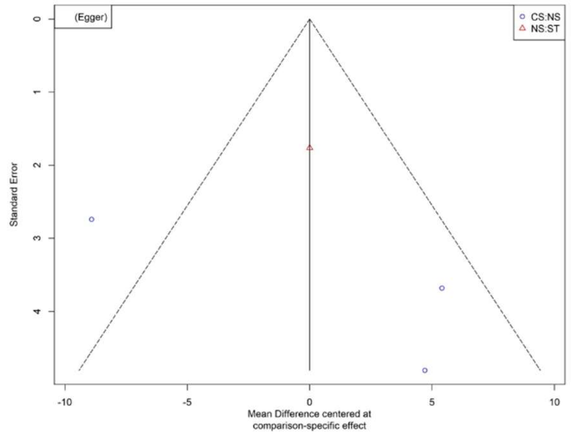

Figure S8.6. A, Evidence plot showing direct and indirect comparisons of change in full mouth plaque score (FMPS) in different nicotine-containing product user groups and non-smokers; B, Treatment ranking with P-scores under the random-effects model where higher values indicate larger outcome values, associated with favorable outcome between baseline and re-evaluation. C, League table shows the mean difference and 95% confidence interval for direct (upper right triangle) and network (lower left triangle) comparisons; D, Funnel plot to assess small-study effects NS, non-smokers; CS, cigarette smokers; ST, smokeless tobacco.
