# Supplementary material for: Impact of Traditional and Alternative Nicotine-Containing Product Use on Non-Surgical Periodontal Therapy: A Systematic Review and Network Meta-Analysis
Source: Dent J (Basel). 2026 Sep 22;14(10):616. doi: 10.3390/dj14100616 (PMC13703454; doi:10.3390/dj14100616)
Supplement: Supplementary file 1 [file dentistry-14-00616-s001.zip › Table S1 (Basic characteristics).pdf]

Table S1. General and periodontal treatment related characteristics of included studies; CS, cigarette smokers; EC, e-cigarette users; NS, non-smokers; FS, former smokers; Q, quitters; ST, smokeless tobacco users; BOP, bleeding on probing, PI, plaque index, FMPS, full mouth plaque score; GI, gingival index, PPD, probing pocket depth; GCF, gingival crevicular fluid; MBL, marginal bone loss; SRP, scaling and root planning, RSD, root surface debridement; NSPT, nonsurgical periodontal therapy; CP, chronic periodontitis; RCT, randomized controlled trial; OHI, oral hygiene instructions

*Not included in the quantitative synthesis, as mean ± SD or mean ± SE was not available in the article.

Data are shown as mean ± SD if available

Study (Year)	Study type	Country	Study participants (n) (M/F)	Mean Age ± SD (year)	Smoking status (mean duration ± SD (year) / mean daily frequency ± SD)	Periodontal status	Non-surgical treatment	Examined site	Follow-up time	Outcomes
***Abreu et al. (2019) [30]**	prospective cohort	Brazil	CS: 15 (9/6)<br>NS: 16 (6/10)	CS: NA<br>NS: NA	CS: smoked ten or more cigarettes a day for at least 2 years. (NA/NA)<br>NS: non-smokers, former smokers excluded<br>confirmation of smoking status: verbally at each visit	severe generalized CP in at least two teeth in different arches, including bleeding when probing these sites, PPD ≥5 mm, CAL ≥5 mm and radiographic bone loss involving >30% of site	Quadrant SRP were performed weekly at baseline on each participant under local anesthesia, using periodontal curettes and ultrasonic scalers + OHI, maintenance therapy included professional plaque control and SRP in recurrent periodontal pockets monthly	full mouth: six sites *per* tooth on all the teeth, excluding third molars	3 months	PPD*<br>CAL*<br>BOP*<br>FMPS*
***Akpinar et al. (2013) [31]**	prospective cohort	Turkey	CS: 14 (9/5)<br>NS: 15 (7/8)	CS: 38.4±5.5<br>NS: 37.7±5.9	CS: regularly smoked ≥20 cigarettes/day (NA/NA)<br>NS: not having smoked cigarettes in their lifetime<br>confirmation of smoking status: self-reporting	generalized CP characterized by at least 30% teeth with pockets >5 mm	SRP procedure was performed quadrant per quadrant under local anesthesia in four visits using specific curettes + OHI	at least one moderate (4–6 mm) and at least one deep (>6 mm) pockets of teeth per subject	6 weeks	PPD*<br>CAL*<br>BOP*<br>PI*<br>GI*
**AlAhmari et al. (2019) (SRP alone) [35]**	RCT	Saudi Arabia	CS: 22 (22/0)<br>NS: 21 (21/0)	CS: 45.2 ± 3.6<br>NS: 44.2 ± 2.4	CS: Self-reported habitual cigarette-smokers (11.7±0.3/NA)<br>NS: self-reported never-smokers<br>confirmation of smoking status: self-reporting	patients with CP (presence of at least 30% sites with ≥ 3mm of CAL and PPD ≥3mm	SRP was performed in one session under local anesthesia using handheld Gracey curettes No. 5/6	full mouth: at 6 sites per tooth on all maxillary and mandibular teeth	12 weeks	PPD<br>CAL<br>BOP<br>FMPS

**Al-Hamoudi et al. (2020) [32]**	prospective cohort	Saudi Arabia	EC:36 (32/4)<br><br>NS:35 (30/5)	EC: 47.7±5.8<br><br>NS: 46.5±3.4	EC: vaped at least once daily for the past 12 months and did not use any other tobacco products (3.3±0.5/ 17.6±3.1)<br><br>NS: never having used any form of tobacco product<br><br>confirmation of smoking status: self-reporting	CP diagnosis was based on the following criteria: GI >1, CAL ≥3 mm, PD ≥4 mm, and radiographic evidence of MBL ≥3 mm below the cementoenamel junction	SRP	full mouth: at 6 sites per tooth excluding third molars	3 months	PPD<br>CAL<br>PI<br>GI<br>GCF
**Al-Kheraif et al. (2022) (RSD alone) [33]**	RCT	Saudi Arabia	CS:15 (13/2)<br><br>NS:14 (10/4)	CS: 42.2 ± 3.8<br><br>NS: 40.5 ± 2.6	CS: ≥10 cigarettes/day for five years (8.7± 3.1/ NA)<br><br>NS: never-smokers<br><br>confirmation of smoking status: salivary cotinine evaluation	stage-III generalized CP with PPD ≥6 mm, CAL ≥5 mm, and MBL in contralateral teeth, tooth loss ≤4 teeth	RSD where the plaque (supra and subgingival) was thoroughly removed under local anesthesia via ultrasonic scaling and Gracey curettes in two visits + OHI	split-mouth design, at 6 sites of the tooth on each tooth	6 months	PPD<br>CAL<br>BOP<br>FMPS
**Al-Kheraif et al. (2022) (DS alone) [34]**	RCT	Saudi Arabia	CS:13 (11/2)<br><br>NS:13 (12/1)	CS: 45.2 ± 4.8<br><br>NS: 43.5 ± 3.6	CS: smoking ≥10 cigarettes per day for at least 5 years (8.7±3.1/NA)<br><br>NS: never-smokers<br><br>confirmation of smoking status: salivary cotinine evaluation	stage-II generalized CP with PPD ≤ 5 mm, CAL of 3–4 mm and horizontal alveolar bone loss in contralateral teeth	Dental scaling was performed under local anesthesia with the help of specialized Gracey curette and ultrasonic scalers + OHI	split-mouth design, at 6 sites of the tooth on each tooth	6 months	PPD<br>CAL<br>BOP<br>FMPS
**Apatzidou et al (2005) [36]**	prospective cohort	Greece	CS:15 (9/6)<br><br>NS:25 (14/11)	CS: NA<br><br>NS: NA	CS: smoking ≥5 cigarettes per day (NA/NA)<br><br>NS: non-smokers<br><br>confirmation of smoking status: cotinine enzyme inhibition assay	CP with at least two non-adjacent sites per quadrant with a PPD of 5 mm or over and radiographic evidence of bone loss	full-mouth SRP or quadrant SRP at two-weekly intervals were performed on each patient under local anaesthesia using an assortment of periodontal curettes and ultrasonic scalers, pockets were irrigated with saline + OHI	at 6 sites/tooth; 1 site/quadrant with the deepest PPD, (not less than 5mm), from each patient for selected site clinical analysis.	6 months	PPD<br>CAL<br>BOP
**Ardais et al. (2014) [37]**	quasi-experimental study	Brazil	CS: 11 (4/7)<br><br>NS: 14 (5/9)	CS: 47.00±7.81<br><br>NS: 47.64±10.81	CS: ≥10 cigarettes/day for ≥6 months (25.68±9.52/ 19.27±3.28)<br><br>NS: never-smokers<br><br>confirmation of smoking status: self-reporting	four teeth with CAL ≥ 5 mm, PPD ≥ 5 mm, and BOP at one or more sites.	NSPT: removal of all biofilm-retentive factors, oral hygiene instruction, and scaling and root planing under local anesthesia.	full mouth: at 6 sites per tooth excluding third molars	3 months	PPD<br>CAL<br>BOP<br>FMPS

***Arikan et al. (2023) [38]**	prospective cohort	Turkey	CS: 20 (13/7)<br>NS: 20 (10/10)	CS: NA<br>NS: NA	CS: ≥10 cigarettes/day for ≥5 years (NA/NA)<br>NS: never smoked<br>confirmation of smoking status: self-reporting	stage III grade C periodontitis: at least 5 non-adjacent interdental sites with interdental PPD≥ 6mm, CAL≥5mm (due to periodontal causes), and BOP≥30%, had not lost more than four teeth due to periodontitis.	SRP was performed by using ultrasonic instruments and Gracey curettes under local anesthesia twice a week in 4 sessions +OHI	full mouth: at 6 sites per tooth excluding third molars	3 months	PPD*<br>CAL*<br>PI*<br>BOP*<br>GI*
**Aziz et al. (2015) [39]**	longitudinal study	India	CS: 80 (NA/NA)<br>NS: 51 (NA/NA)	CS: 44.1±5.81<br>NS: 40.9±4.6	CS: smoking habit for over 3 years and frequency of smoking ≥ 5 cigarettes/day (NA/NA)<br>NS: never smoker<br>confirmation of smoking status: self-reporting	severe CP (CAL ≥ 5 mm)	SRP performed by qualified periodontologists using an ultrasonic instrument and manual Gracey curettes + OHI	full mouth: six sites around each tooth	3 months	PPD<br>CAL<br>PI<br>GI
**Buduneli et al. (2009) [40]**	prospective cohort	Turkey	CS: 10 (6/4)<br>NS:10 (4/6)	CS: 48±4.4<br>NS: 44.54±5.53	CS: ≥10 cigarettes/day for ≥5 years (NA/NA)<br>NS: have never smoked<br>confirmation of smoking status: self-reporting	moderate CP, with at least two interproximal sites with CAL≥4 mm and PPD≥5 mm not on the same tooth, two of which were in the anterior region	SRP under local anesthesia for 9 to 12 minutes per tooth	sampling sites were PPD≥5 mm, CAL≥4 mm, and the presence of BOP in single-rooted teeth	4 weeks	PPD<br>CAL<br>PI<br>GI<br>GCF
***Camargo et al. (2016) [41]**	prospective cohort	Brazil	CS: 24 (12/12)<br>NS: 24 (11/13)	CS: 44.66±10.22<br>NS: 53.08±10.26	CS: ≥10 cigarettes/day for ≥2 years (24 ±11.73/19.83±10.1)<br>NS: non-smokers<br>confirmation of smoking status: verbally confirmed at each visit	periodontal disease in at least two teeth in different arches, bleeding on probing on these sites where PPD ≥ 5 mm; and radiographic evidence of bone loss ranging from 30 to 50%.	quadrant SRP was performed weekly under local anesthesia using periodontal curettes and ultrasonic scalers, maintenance therapy included professional plaque control and monthly quadrant SRP in recurrent periodontal pockets throughout the study period	full mouth: at 6 sites per tooth excluding third molars	6 months	PPD*<br>CAL*<br>FMPS*<br>BOP*
***Chandra et al. (2011) (control)** [42]	prospective cohort	India	CS:3 (NA/NA)<br>NS: 3 (NA/NA)	CS: NA<br>NS: NA	CS: NA (NA/NA)<br>NS: NA<br>confirmation of smoking status: self-reporting	chronic generalized periodontitis, periodontal pocket measuring 5-7 mm in depth in different quadrants of the mouth and signs of bone loss on clinical and radiographic examination	SRP	141 sites from six patients (67 sites from three non-smoker patients and 74 sites from three smoker patients) with PPD 5-7	90 days	PI*<br>GI*

**Chandra et al. (2012) (placebo) [43]**	RCT	India	CS: 48 (48/0)<br><br>NS: 48 (48/0)	CS:29.8±5.63<br><br>NS: 33.90±7.66	CS: ≥10 cigarettes a day for a minimum of 5 years (NA/18.10±4.33)<br><br>NS: non-smokers<br><br>confirmation of smoking status: self-reporting	CP presenting with a minimum of four periodontal pockets with PPD ≥5 mm	SRP was performed by using a piezoelectric ultrasonic scaling unit under water spray at a medium power setting and 4R/4L Columbia University curette	interdental suprabony periodontal pockets in molars with PPD ≥5 mm	6 months	PPD<br>CAL<br>GI<br>PI
**D'Aiuto et al. (2005) [44]**	prospective cohort	United Kingdom	CS:40 (NA/NA)<br><br>NS:57 (NA/NA)	CS: NA<br><br>NS: NA	CS: NA (NA/NA)<br><br>NS: NA<br><br>confirmation of smoking status: self-reporting	severe generalized periodontitis, PPD ≥5 mm with BOP and MBL >30% in at least 50% of the dentition	OHI and subgingival SRP under local anesthesia were performed using a piezoelectric instrument equipped with appropriate subgingival tips	full mouth: at 6 sites per tooth	6 months	PPD
**Darby et al. (2005) (CP) [45]**	prospective cohort	United Kingdom	CS:10 (NA/NA)<br><br>NS:18 (NA/NA)	CS: (NA/NA)<br><br>NS: (NA/NA)	CS: smoked between five and 30 cigarettes a day. (NA/NA)<br><br>NS: NA<br><br>confirmation of smoking status: self-reporting	CP, at least two non-adjacent sites per quadrant with pocket depth of at least 5 mm	quadrant SRP under local anaesthesia over a 4-week period.	In each patient, 4 sites with PPD≥5 mm were selected, where possible one in each quadrant, with no furcation involvement.	8 weeks	PPD<br>GI<br>PI<br>BOP*
**Do et al. (2023) [46]**	quasi-experimental study	Viet Nam	CS: 20 (20/0)<br><br>NS: 20 (20/0)	CS: 47.65 ± 8.38<br><br>NS: 44.65 ± 8.31	CS: smoked more than 10 cigarettes per day for at least the past 10 years (NA/NA)<br><br>NS: never smoked<br><br>confirmation of smoking status: self-reporting	moderate to severe periodontitis: gingival bleeding on probing at examination, ≥5 mm PPD, bone resorption on panoramic dental X-ray films ≥16% or >3 mm root length; having at least two sites at anterior teeth with PPD from 5 to 7 mm.	OHI+ SRP by ultrasonic scalers and Gracey curettes under local anesthesia performed by a periodontist, number of SRP visits was determined by patient disease conditions	full mouth	3 months	PPD<br>CAL<br>GI*<br>PI*<br>BOP*
**Eltas et al. (2012) (placebo) [47]**	RCT	Turkey	CS:26 (13/13)<br><br>NS: 26 (13/13)	CS: 42.7±5.1<br><br>NS: 44.1±4.9	CS: >10 cigarettes a day for > 5 years (NA/17 ± 6)<br><br>NS: never having smoked<br><br>confirmation of smoking status: self-reporting	generalized moderate CP with the presence of at least 2 teeth with PPD between 4 and 6 mm and radiographic signs of bone loss per quadrants	OHI and full mouth supragingival and subgingival SRP with the combined use of hand and ultrasonic instruments under local anesthesia in the same session	test sites: PPD between 4 and 6 mm	6 months	PPD<br>CAL<br>GI<br>PI<br>GCF

**Erdemir et al. (2004) [49]**	prospective cohort	Turkey	CS:22 (NA/NA)<br>NS:19 (NA/NA)	CS: 44.41±7.88<br>NS:46.94±6.07	CS: daily smoked 20 cigarettes (NA/NA)<br>NS: never smoked tobacco<br>confirmation of smoking status: self-reporting	moderate-to-severe periodontal disease as evidenced by multiple sites with a PPD of 5 mm or more and bone loss by radiographs	OHI + SRP	GI, PI, BOP: full mouth<br>PPD, CAL: sample sites (4 sites of each patient with PPD≥5 mm)	6 months	PPD<br>CAL<br>GI<br>PI<br>BOP
***Erdemir et al. (2007) [48]**	prospective cohort	Turkey	CS:45 (NA/NA)<br>NS:43 (NA/NA)	CS: 45.5±8.5<br>NS: 45.9±8.0	CS: daily smoked 20 cigarettes (17.5±1.7 /NA)<br>NS: never smoked tobacco<br>confirmation of smoking status: self-reporting	chronic periodontal disease as evidenced from a probing depth of 6mm or more at 80% of the proximal sites and radiographic bone loss of more than 50%	Full mouth supragingival professional tooth cleaning (scaling and polishing) was performed in a single session for 60 min. and OHI were given at this time. One week later, the subsequent non-surgical treatment consisted of sub-gingival debridement using Gracey curettes under local anaesthesia in one session for 60 min.	full mouth: at six sites per tooth of all teeth	6 months	PPD*<br>CAL*<br>GI*<br>PI*
**Feres et al. (2015) [50]**	prospective cohort	Brazil	CS: 15 (8/7)<br>NS: 15 (8/7)	CS: 40.5±8.2<br>NS: 42.1±6.5	CS: smoked at least 10 cigarettes per day within the past five years (26.6±7.3/17.1±7.3)<br>NS: never smoked before<br>confirmation of smoking status: self-reporting	minimum of six teeth with at least one site each with a PPD between 5–7 mm and CAL between 5–10 mm.	SRP was performed using manual instruments in six visits lasting one hour each, over a period of 21 days.	full mouth: at 6 sites per tooth excluding third molars	6 months	PPD<br>CAL<br>FMPS<br>BOP
**Gaind et al. (2024) [51]**	prospective cohort	India	CS:20 (NA/NA)<br>NS:20 (NA/NA)	CS: 40 ± 2<br>NS: 37 ± 2	CS: NA (NA/NA)<br>NS: NA<br>confirmation of smoking status: self-reporting	CP	SRP: ultrasonic scaler was used for supra- and subgingival scaling, and Gracey curettes were used for root planning + OHI	full mouth	3 months	PI<br>GI
**Ghallab et al. [52] (2010)**	prospective cohort	Egypt	CS:11(7/4)<br>NS:11 (3/8)	CS: 39.2±4.9<br>NS: 42±8	CS: smoked ≥20 cigarettes of the same brand per day for >5 years (NA/NA)<br>NS: who had never smoked.<br>confirmation of smoking status: self-reporting	moderate CP, having PPD ≤ 6 mm and CAL ≤ 4 mm	Full-mouth SRP performed by the same operator, use of ultrasonic devices and periodontal Gracey curets under local anesthesia + OHI	full mouth: at 6 sites per tooth excluding third molars	1 month	PI<br>GI

**Grossi et al. (1997) [53]**	prospective cohort	USA	CS:60 (NA/NA)<br>FS: 55 (NA/NA)<br>NS:28 (NA/NA)	CS:43.1±6.4<br>FS:49.4±8.2<br>NS:46.5±6.5	CS: currently smoking regardless of the number of cigarettes smoked and frequency. (19.9±13.0/NA)<br>FS: smoked in the past but had quit 1 year ago or longer (21.9±21.6/NA) (quit years:10.8±8.4)<br>NS: had never smoked<br>confirmation of smoking status: self-reporting	established periodontitis: two interproximal sites with attachment loss ⩾6 mm; one additional interproximal site with pocket depth ⩾5 mm.	one session of supragingival scaling and OHI, then four to six sessions of subgingival SRP administered by quadrant with the patients under local anesthesia. Subgingival scaling included use of ultrasonic devices and hand instrumentation.	full mouth: at 6 sites per tooth excluding third molars	3 months	PPD<br>CAL<br>GI<br>PI
**Guentsch et al. (2008) [54]**	prospective cohort	Germany	CS:15 (8/7)<br>NS:15 (6/9)	CS: 41.0±8.8<br>NS: 46.3±13.1	CS: current smoking of at least ten cigarettes per day and a minimum of four packs per year (NA/19.0±4.7)<br>NS: never-smokers<br>confirmation of smoking status: self-reporting	generalised CP characterised by at least 30% teeth with pockets >5 mm	full-mouth SRP (performed in all four quadrants under local anaesthesia at one appointment) and then a period of periodontal maintenance (4, 12 and 24 weeks after initial treatment) and monitoring of oral hygiene	full mouth: at 6 sites per tooth	6 months	PPD<br>BOP
**Guru et al. (2018) [55]**	prospective cohort	India	CS: 25 (25/0)<br>NS: 25 (19/6)	CS:36.4±8.2<br>NS:36.3±8.4	CS: smoking ≥20 cigarettes/day for more than 2 years (NA/NA)<br>NS: non-smokers<br>confirmation of smoking status: self-reporting	generalized moderate-to-severe CP, with clinical attachment loss ≥3 mm	full-mouth SRP by means of ultrasonic instruments followed by root planing using Gracey curettes	full mouth	1 months	PPD<br>CAL<br>PI
**Hadžić et al. (2023) [56]**	prospective cohort	Croatia	CS: 7 (5/2)<br>NS: 7 (3/4)	CS: 43.28±NA<br>NS: 40.14±NA	CS: smokers (10.17±3.84/NA)<br>NS: non-smokers<br>confirmation of smoking status: self-reporting	stage III or IV periodontitis with at least 20 teeth	Supragingival and subgingival periodontal therapy were performed as full-mouth therapy with machine-driven and hand instruments under local anesthesia.	full mouth: at 6 sites per tooth	3 months	PPD<br>CAL<br>FMPS<br>BOP
***Hendek et al. (2015) [57]**	case-control study with intervention arm	Turkey	CS:24 (15/9)<br>NS:23 (12/11)	CS:NA<br>NS:NA	CS: current smoking ≥10 years and ≥10 cigarettes per day (NA/NA)<br>NS: never-smokers<br>confirmation of smoking status: self-reporting	patients with CP had teeth with 30% periodontal bone loss, presence of at least two non-adjacent sites per quadrant with probing pocket depths ≥5 mm and bleeding on probing	initial periodontal therapy including SRP and polishing within 14 days under local anesthesia with specific curettes and OHI	full mouth: at 6 sites per tooth; 8 GCF sample teeth/patient with PPD 4-7 mm and ≥30% bone loss	3 months	GI*<br>PI*<br>PPD*<br>CAL*<br>GCF*

**Hendek et al. (2020) [59]**	prospective cohort	Turkey	CS:20 (11/9)<br><br>NS:20 (10/10)	CS: 44.60 ± 5.93<br><br>NS: 44.10 ± 7.54	CS: smoking ≥10 years and ≥10 cigarettes per day (NA/NA)<br><br>NS: never-smokers<br><br>confirmation of smoking status: self-reporting	CP, teeth with 30% periodontal bone loss, presence of at least two nonadjacent sites per quadrant with probing pocket depths of ≥ 5 mm, and bleeding on probing	SRP under local anesthesia using manual and ultrasonic devices in a single appointment + OHI	full mouth: at 6 sites per tooth; sample teeth: single-rooted teeth with ≥4 and <7 mm PPD and ≥30% bone loss	6–8 weeks	PPD<br>CAL<br>GI<br>PI<br>GCF
**Ikhar et al. (2024) [58]**	prospective cohort	India	CS:20 (20/0)<br><br>NS:20 (12/8)	CS: 49.06 ± 7.33<br><br>NS: 43.97 ± 5.68	CS: history of smoking at least 10 cigarettes per day for the past 3 years. (NA/NA)<br><br>NS: no history of smoking<br><br>confirmation of smoking status: self-reporting	Stage III periodontitis as assessed by clinical findings of PPD ≥5 mm and CAL ≥ 4 mm (≥ 30% of teeth affected) and with radiographic evidence of bone loss	NSPT, including SRP performed by a single operator along with OHI, on recall visits every month up to 3 months, supragingival plaque was removed and OHI were reinforced	full mouth: at 6 sites per tooth	3 months	PPD<br>CAL<br>GI<br>PI
**Lütfioğlu et al. (2021) [60]**	prospective cohort	Turkey	CS: 15 (8/7)<br><br>NS:15 (9/6)	CS: 42.6 ± 6.51<br><br>NS: 50.66 ± 6.91	CS: ≥10 cigarettes/day for >5 years (NA/NA)<br><br>NS: never smoked in the lifetime<br><br>confirmation of smoking status: self-reporting	Generalized periodontitis stage III, grade A and C (existence of minimum 30% teeth in a total teeth number (that should not be under 16 teeth) per mouth with ≥5 mm PPD, ≥6 mm CAL, and evident alveolar bone loss on radiograph	NSPT included detailed OHI and reinforcement in conjunction with SRP (initially done as full-mouth instrumentation and then repeated at required sites during consecutive visits with 1-week intervals, completed within 2-3 visits) Severe cases received deeper SRP under local anesthesia.	full mouth: at 6 sites per tooth, each highest value per tooth was taken for evaluations	4 weeks	PPD<br>CAL<br>GI<br>PI<br>BOP
**Maged et al. (2022) [29]**	non-randomized clinical trial	Egypt	CS:20 (18/2)<br><br>NS:20 (14/6)	CS: 33.6±7.6<br><br>NS: 34.4±7.4	CS: smoker (NA/NA)<br><br>NS: non-smoker<br><br>confirmation of smoking status: self-reporting	Stage II-III Grade B periodontitis who had minimally two non-adjacent teeth with sites measuring 3 to 4 mm or ≥ 5 mm interdental CAL and 15% to 33% or radiographic bone loss that extends to middle third of root and beyond	full mouth supra-gingival scaling performed using ultrasonic device with supragingival inserts for gross scaling followed by subgingival debridement using Gracey's curettes in a single visit.	full mouth: at 6 sites per tooth	3 months	PPD<br>CAL<br>PI<br>GI
**Mantyla et al. (2006) [61]**	longitudinal study	Finland	CS:11 (NA/NA)<br><br>NS:5 (NA/NA)	CS: NA<br><br>NS: NA	CS: ≥20 cigarettes per day and ≥10 years of smoking (NA/NA)<br><br>NS: nonsmokers<br><br>confirmation of smoking status: self-reporting	CP, at least five sites exhibiting ≥4 mm PPD and radiographic bone loss	bimonthly SRP and oral hygiene instructions	7–10 sites/patient with PPD ≥4 mm	12 months	PPD<br>CAL<br>BOP*
**Mashalkar et al. (2024) [62]**	prospective cohort	India	CS: 50 (30/20)<br><br>NS: 50 (30/20)	CS: 45.2 ± 6.5<br><br>NS: 43.8 ± 7.2	CS: smokers (NA/NA)<br><br>NS: non-smokers<br><br>confirmation of smoking status: self-reporting	moderate to severe CP, minimum of 20 teeth, PPD ≥ 5 mm, CAL ≥ 3 mm, and radiographic evidence of bone loss	NSPT, consisting of thorough SRP performed under local anesthesia using ultrasonic scalers and hand instruments + OHI, recall every three months	full mouth	6 months	PPD<br>CAL<br>PI<br>BOP

**Maske et al. (2023) (control) [63]**	RCT	India	CS:20 (NA/NA)<br>NS:20 (NA/NA)	CS: NA<br>NS: NA	CS: smoking 10 cigarettes/day from last 3 years (NA/NA)<br>NS: not having smoked cigarettes in their lifetime<br>confirmation of smoking status: self-reporting	CP: diseased site had probing pocket depth ≥ 5 mm and clinical attachment level ≥5mm	Scaling and root planing was performed by a single operator and oral hygiene instructions were given to the patients	split mouth study, total 80 sites with PPD≥ 5 mm	6 months	PI<br>GI<br>PPD<br>CAL
**Meenawat et al. (2015) [64]**	prospective cohort	India	CS: 12 (12/0)<br>NS: 12 (12/0)	CS: NA<br>NS:NA	CS: smoking >10 cigarettes/day for >2 years (14.58 ± 4.19 /14.92 ± 4.36)<br>NS: non-smokers<br>confirmation of smoking status: cotinine levels were evaluated in saliva using Accutest® NicAlert™ test strip	CP: having minimum of 20 remaining teeth,with periodontal disease as evidenced by at least 4 tooth sites with PPD >4 mm, CAL >2mm; and radiographic evidence of bone loss >2 mm from the cementoenamel junction	Scaling and root planing	full mouth	1 months	PPD<br>CAL<br>PI<br>GI
**Mittal et al. (2018) [65]**	prospective cohort	India	CS:50 (50/0)<br>ST:50 (50/0)<br>NS:50 (24/26)	CS: 35.32±NA<br>ST: 31.44±NA<br>NS: 37.40±NA	CS: history of tobacco consumption for a minimum duration of 2 years (NA/NA)<br>ST: history of tobacco consumption for a minimum duration of 2 years (NA/NA)<br>NS: non-smokers<br>confirmation of smoking status: self-reporting	CP: at least four teeth with pocket depth > 3 - 5 mm and/or attachment loss of 1 - 3 mm	Scaling and root planing was performed at the initial visit as a part of initial therapy. Routine oral hygiene instructions were given and were reinforced at every visit	full mouth	3 months	PPD<br>PI<br>GI<br>GCF
**Mouzakiti et al. (2012) [66]**	prospective cohort	Greece	CS:15 (6/9)<br>NS:15 (5/10)	CS: 49.3 ± 8.35<br>NS: 51.7 ± 6.15	CS: daily cigarette consumption ≥20 cigarettes for at least 10 years (NA/NA)<br>NS: non-smokers<br>confirmation of smoking status: self-reporting	CP: had at least 20 teeth and at least seven teeth with probing depths ≥5 mm	nonsurgical treatment following the protocol of full-mouth disinfection without using antibacterial products	full mouth	6 weeks	PPD<br>CAL<br>FMPS<br>BOP
**Öngöz-Dede et al. (2023) [67]**	prospective cohort	Turkey	CS:15 (8/7)<br>NS:15 (8/7)	CS:42.87±10.04<br>NS:45.60±6.87	CS: smoked ≥10 cigarettes/day for ≥5 years<br>NS: no previous history of smoking<br>confirmation of smoking status: self-reporting	generalized stage 3 grade B periodontitis: interdental clinical CAL≥ 5 mm, PPD≥6 mm on at least two non-adjacent teeth, and radiographic bone loss extending to the middle or apical third of the root, attachment and bone loss at ≥30% of the teeth and loss of ≤4 teeth due to periodontitis	Subgingival instrumentation (debridement, SRP) using manual scalers, curettes, and ultrasonic instruments was performed on all 4 quadrants of patients with periodontitis and maintenance and a repeat of oral hygiene.	full mouth: six sites around each tooth	6 weeks	PPD<br>CAL<br>PI<br>GI<br>BOP

**Özdemir et al. (2021) (control) [68]**	RCT	Turkey	CS:15 (8/7)<br>NS:15 (7/8)	CS:37.7±2.2<br>NS: 38.7±2.2	CS: smoking at least 20 cigarettes a day.(NA/NA)<br>NS: nonsmokers<br>confirmation of smoking status: self-reporting	periodontitis: at least two bilateral single-rooted teeth with ≥5mm PPD	SRP was performed with local anesthesia in one day within two appointments. Nonsurgical periodontal treatment was performed using ultrasonic devices and periodontal curettes	full mouth: on six surfaces of each tooth except third molars	6 weeks	PPD<br>CAL<br>PI<br>GI<br>BOP*
***Padhye et al. (2019) [69]**	longitudinal study	India	CS:15 (NA/NA)<br>FS:15 (NA/NA)<br>NS:15 (NA/NA)	CS: NA<br>FS: NA<br>NS: NA	CS: smoking ≤10 cigarettes/day for a minimum of 2 years<br>FS: who had quit smoking for ≥6 months<br>NS: non-smokers<br>confirmation of smoking status: self-reporting	CP with PPD ≥5 mm and CAL ≥4 mm	full mouth SRP in one sitting using ultrasonic scaler and/or Gracey curettes + OHI	full mouth	12 weeks	GI*<br>BOP*<br>PPD*<br>CAL*
**Palmer et al. (1999) (SRP) [70]**	prospective cohort	United Kingdom	CS:9 (NA/NA)<br>NS:18 (NA/NA)	CS: 50±NA<br>NS: 50.7±NA	CS: NA (NA/NA)<br>NS: NA<br>confirmation of smoking status: self-reporting	moderate to advanced periodontitis	OHI + subgingival scaling by ultrasonic instrumentation under local anaesthetic.in 2 appointments of 90 min each, 1 week apart,	sites with baseline probing depths equal to or greater than 5 mm	6 months	PPD<br>FMPS<br>BOP
***Pamuk et al. (2017) (SRP+Sham) [71]**	split-mouth RCT	Turkey	CS:15 (NA/NA)<br>NS:15 (NA/NA)	CS: NA<br>NS:NA	CS: smoked ≥15 cigarettes per day for ≥5 years (NA/NA)<br>NS: no previous history of smoking<br>confirmation of smoking status: self-reporting	generalized chronic periodontitis: ≥30% of sites with both probing depth ≥5 mm and clinical attachment levels ≥5 mm as well as ≥30% radiographically observable alveolar bone loss.	Non-surgical periodontal treatment consisted of full-mouth SRP performed in a single appointment under local anesthesia using scalers, curettes and ultrasonic devices +OHI	For each subject, five teeth with the highest probing depth and clinical attachment level values	30 days	PI*<br>GI*<br>BOP*<br>PPD*<br>CAL*
**Preber et al. (1986) [72]**	prospective cohort	Sweden	CS:10 (NA/NA)<br>NS:10 (NA/NA)	CS: NA<br>NS: NA	CS: regular smokers for at least 15 years, had a present consumption of 20 cigarettes/day or more (NA/NA)<br>NS: NA<br>confirmation of smoking status: self-reporting	moderate to severe periodontitis	non-surgical treatment program including instruction and motivation for an efficient oral hygiene and a thorough debridement of supra- and subgingival plaque and calculus; six or seven 1-h appointments completed within *4-6* months.	full mouth: on four sites of all teeth and pathological pockets (≥4 mm)	1 month	PI<br>BOP

**Preber et al. (1985) [73]**	prospective cohort	Sweden	CS: 40 (17/23)<br><br>NS: 35 (13/22)	CS: 39.5±NA<br><br>NS: 41.8±NA	CS: regular smokers for at least 5 years, their present consumption being 20 cigarettes or more per day. (NA/NA)<br><br>NS: non-smokers<br><br>confirmation of smoking status: self-reporting	moderate to severe periodontitis	OHI and debridement of supra and subgingival plaque and calculus with hand instrumentation through scaling, root planing and polishing	full mouth	1 month	PPD<br>PI
**Preber et al. (1995) [74]**	prospective cohort	Sweden	CS: 17 (4/13)<br><br>NS: 15 (7/8)	CS: 48.5±NA<br><br>NS: 49.1±NA	CS: smokers (23.6±NA /21.8±NA)<br><br>NS: who had never smoked<br><br>confirmation of smoking status: self-reporting	moderate to severe periodontitis: at least 4 sites with PPD of 6 mm or more and radiographic evidence of alveolar bone loss	nonsurgical intervention program including subgingival debridement and OHI. The program was completed within 6 months, including 6-8 treatment sessions	full mouth and 1 site with a PPD of more than 5 mm	2 months	GI<br>PI<br>PPD
***Preshaw et al. (1999) [76]**	prospective cohort	United Kingdom	CS: 15 (NA/NA)<br><br>FS: 14 (NA/NA)<br><br>NS: 12 (NA/NA)	CS: NA<br><br>FS: NA<br><br>NS: NA	CS: current smokers (NA/16±NA)<br><br>FS: stopped smoking at least 2 years prior to study commencement<br><br>NS: never smoked	untreated, moderately advanced chronic adult periodontal disease	intensive course of SRP: combination of hand and ultrasonic instruments for approximately 6 minutes per tooth, completed over a maximum of 4 visits within this period, each appointment lasting approx. 60 minutes + OHI	Eight periodontally involved test sites per subject (Interproximal sites in the posterior dentition, not including pockets around third molars)	7 months	PI*<br>PPD*<br>BOP*
**Preshaw et al. (2005) [75]**	prospective cohort	United Kingdom	CS:10 (5/5)<br><br>Q:10 (4/6)	CS: 41.4±4.9<br><br>Q: 45.3±9.9	CS: smokers/non-quitters (22.7±5.3/NA)<br><br>Q: smokers who had expressed an interest in quitting the habit. (25.0±18.0/NA)<br><br>confirmation of smoking status: carbon monoxide (CO) reading using a CO meter and salivary cotinine levels were measured using a chair-side colorimetric assay test kit	moderate-to-severe chronic periodontitis and at least six posterior teeth, each with at least one inter-proximal site with PPD ≥5mm and alveolar bone destruction at least one third of the root length.	OHI + non-surgical management using local anaesthetic, root surface instrumentation using manual and ultrasonic instruments, four and six treatment visits, each lasting 45–60 min	full mouth and diseased sites (>3 mm)	12 months	PPD<br>CAL<br>BOP<br>FMPS
**Pucher et al. (1997) [77]**	prospective cohort	USA	CS: 33 (13/20)<br><br>NS: 54 (28/26)	CS: 46.5+9.7<br><br>NS: 53.7±12.3	CS: smoker (NA/NA)<br><br>NS: non-smoker<br><br>confirmation of smoking status: self-reporting	moderate to advanced periodontitis characterized by the presence of at least four teeth with PPD of 5 to 8 mm.	SRP was performed under local anesthetic,with the goal of removing as much calculus from the root surface and attaining smooth root surfaces within one hour	target sites (PPD 5-8 mm, bleeding upon probing)	9 months	PPD<br>CAL<br>PI<br>GI<br>BOP

**Renvert et al. (1998) [78]**	prospective cohort	Sweden	CS:13 (NA/NA)<br>NS:15 (NA/NA)	CS:43±NA<br>NS:45±NA	CS: smoking ≥15 cigarettes per day (NA/NA)<br>NS: non-smoker<br>confirmation of smoking status: self-reporting	untreated advanced periodontal disease: at least 3 sites with BOP and a PPD of 6 mm	OHI+ SRP under local anaesthesia was carried out until the operator felt that the root surfaces were adequately debrided. Three months after completion of the initial treatment the patients were recalled for reinforcement of the OHI and supragingival rubber cup polishing of the teeth	full mouth and experimental sites	6 months	PPD<br>PI<br>FMPS<br>BOP
**Rosa et al. (2011) [79]**	prospective cohort	Brazil	CS:35 (13/22)<br>Q:17 (7/10)	CS: 49.5±7.0<br>Q: 48.9±6.1	CS: non-quitters (NA/NA)<br>Q: smokers willing to quit (NA/NA)<br>confirmation of smoking status: carbon monoxide (CO) concentration measured with a CO monitor	periodontal disease, defined as 30% or more of their teeth with proximal CAL ≥5mm	OHI +supra and subgingival scaling of all teeth using manual and ultrasonic instruments and removal of all intra-oral biofilm retentive factors, 4-6 sessions with a 7-day interval, maintenance program of 3-month intervals.	full mouth and sites presenting baseline PPD ≥4mm	12 months	PPD<br>CAL<br>FMPS<br>BOP
**Ryder et al. (1999) (SRP) [80]**	RCT	USA	CS:61<br>FS:72<br>NS:48	CS:NA<br>FS:NA<br>NS:NA	CS: smoking >10 cigarettes/day (NA/NA)<br>FS: former smokers<br>NS: non-smokers<br>confirmation of smoking status: self-reporting	moderate or severe periodontitis	complete supra and subgingival debridement using ultrasonic instrumentation, and thorough root planing with hand instruments, at the baseline visit and at 4 months.	PPD ≥5mm	9 months	CAL<br>PPD
**Salhi et al. (2020) [81]**	prospective cohort	Belgium	CS: 34 (14/20)<br>FS: 25 (9/16)<br>NS: 32 (14/18)	CS: 46.5±11.5<br>FS: 51.2±11.6<br>NS: 45.0±12.9	CS: current smoker (NA/NA)<br>FS: former smokers<br>NS: non-smokers<br>confirmation of smoking status: self-reporting	Stage III and IV periodontitis	professional mechanical plaque removal, involving a supragingival debridement 2 weeks after the first visit and a full mouth subgingival debridement 2 weeks later using ultrasonic piezoelectric device with specific micro-inserts	full mouth: at 6 sites of each tooth	3 months	PPD<br>CAL<br>FMPS<br>BOP
**Savran et al. (2024) (control) [82]**	retrospective	Turkey	CS: 20 (11/9)<br>NS: 20 (10/10)	CS: 44.7 ± 9.24<br>NS: 44.9 ± 8.09	CS: smoking ⩾10 cigarettes a day (16.32±7.52/19.75±4.43)<br>NS: never-smokers<br>confirmation of smoking status: self-reporting	Stage III Grade B and C periodontitis	full-mouth SI under local anesthetic one week following the supragingival scaling, utilizing Gracey curettes and ultrasonic scalers + subgingival saline irrigation; follow-up appointments one week, one month, and three months later	full mouth: at 6 sites of each tooth	3 months	PPD<br>CAL<br>PI<br>GI<br>BOP

**Shah et al. (2023) [17]**	retrospective	United Kingdom	CS: 20 (11/9)<br><br>FS:60 (22/38)<br><br>EC:20 (9/11)<br><br>NS:120(48/112)	CS: 44.1±10.2<br><br>FS: 49.0±9.8<br><br>EC: 47.8±6.9<br><br>NS: 48.0±12.2	CS: smoking ≥1 cigarette(s) a day, of ≥100 cigarettes in total (20.0±9.9/10.2±6.4)<br><br>FS: who had not smoked for > 1 year up to a maximum of 10 years and previously smoked ≥100 cigarettes (years since quitting smoking: 4.9±3.7)<br><br>EC: former smokers that had quit smoking and using only e-cigarettes of any form (years since quitting smoking: 3.4±2.6)<br><br>NS: who had never used any form of cigarettes/e-cigarettes<br><br>confirmation of smoking status: self-reporting	periodontitis diagnosis (localized/ generalized, all stages and grades)	supra- and subgingival mechanical plaque and calculus removal with ultrasonic scalers and hand curettes (under local anesthesia in PD > 4 mm); time between each session ranged from 1 to 4 weeks.	full mouth	28.7±14.7 weeks	PPD<br>CAL<br>FMPS<br>BOP
**Sheokand et al. (2019) (placebo) [83]**	RCT	India	CS:NA (NA/NA)<br><br>NS:NA (NA/NA)	CS: NA<br><br>NS: NA	CS: smoker (NA/NA)<br><br>NS: non-smoker<br><br>confirmation of smoking status: self-reporting	CP	full mouth scaling and root planing with placebo gel	15-15 sites with PPD ≥5 mm, CAL ≥3 mm and radiographic evidence of vertical osseous defects 3–6 mm	6 months	PI<br>GI<br>PPD<br>CAL
**Toker et al. (2012) [84]**	prospective cohort	Turkey	CS: 15 (9/6)<br><br>NS: 15 (7/8)	CS: 38.4 ± 5.5<br><br>NS: 38.7 ± 5.9	CS: smoking ≥20 cigarettes a day (NA/NA)<br><br>NS: not having smoked cigarettes in their lifetime.<br><br>confirmation of smoking status: self-reporting	generalized CP (> 30% of sites with a clinical attachment level and probing depth of ≥5 mm)	OHI and SRP was performed, quadrant by quadrant, under local anesthesia in four visits using specific curettes	GCF sample site	6 weeks	PPD<br>CAL<br>PI<br>GI<br>BOP*
**Tomasi et al. (2004) (control) [85]**	RCT	Sweden	CS: 20 (6/14)<br><br>NS: 32 (19/13)	CS: 46.7±NA<br><br>NS: 47.6±NA	CS: regular smokers (80% of the subjects were smoking more than 10 cig/day for an average of 25.9 years)<br><br>NS: non-smokers<br><br>confirmation of smoking status: self-reporting	having at least eight periodontal sites with PPD ≥5 mm	single episode of full-mouth supra- and subgingival scaling/root planing using an ultrasonic scaler and curettes + OHI	sites with PPD of ≥5 mm and BOP+, with an average of 22 sites/patient	3 months	PPD<br>FMPS<br>BOP*

**Türkoglu et al. (2016) [86]**	prospective cohort	Turkey	CS: 16 (9/7)<br>NS: 15 (8/7)	CS: 43.5±7.8<br>NS: 46.4±7.2	CS: who had ≥10 cigarettes per day for ≥10 years (19.01±12.2/NA)<br>NS: have never smoked<br>confirmation of smoking status: self-reporting	moderate/severe CP: at least four non-adjacent teeth with sites with CAL ≥4 mm and PPD ≥5 mm.	OHI + SRP using an ultrasonic device and hand instruments completed in 14 days, (number of the visits was determined based on patient's requisites) Root surfaces were instrumented under local anesthesia until the smoothness of the roots was obtained as determined by tactile examination	full mouth (at six sites per tooth excluding third molars) and GCF sampling sites (CAL ≥6 mm and PPD ≥5 mm)	3 months	PPD<br>CAL<br>PI*<br>BOP<br>GCF
**Vajawat et al. (2022) (control) [87]**	split mouth RCT	India	CS: 12 (NA/NA)<br>NS: 12 (NA/NA)	CS: NA<br>NS: NA	CS: average of 15 cigarettes/day for a minimum of past 2 years (NA/NA)<br>NS: never smoked a cigarette in his life<br>confirmation of smoking status: self-reporting	CP patients with ≥20 remaining teeth and contralateral pockets measuring≥5 mm with evident CAL and bone loss shown in radiograph	Full mouth root planing was done using area-specific Gracey curettes under local anesthesia followed by application of placebo gel	split mouth design: sites randomly divided into test and control sites	3 months	PPD<br>CAL
**Varghese et al. (2020) [88]**	case-control	India	CS: 10 (10/0)<br>NS: 10 (10/0)	CS: NA<br>NS: NA	CS: smoking ≥ 10 cigarettes/day for more than 5 years. (NA/NA)<br>NS: not having smoked cigarettes in their lifetime<br>confirmation of smoking status: self-reporting	CP patients by at least 30% teeth with pockets > 5 mm	OHI+ SRP, which were completed in at least 2 appointments.	full mouth	3 months	PPD<br>CAL<br>PI<br>GI
**Vijayakumar et al. (2020) [89]**	comparative interventional	India	CS: 26 (26/0)<br>NS: 26 (26/0)	CS: NA<br>NS: NA	CS: NA (NA/NA)<br>NS: NA<br>confirmation of smoking status: self-reporting	CP: attachment loss of ≥5 mm at more than 30% of the sites, bleeding on probing, and patients with ≥20 functional teeth	SRP	full mouth: at six sites of all teeth	4 weeks	PPD<br>CAL<br>PI<br>GI
**Vohra et al. (2020) (SRP) [90]**	RCT	Saudi Arabia	ST: 32 (32/0)<br>NS: 33 (33/0)	ST: 50.7±1.8<br>NS: 52.8±1.6	ST: have been consuming *shamma* at least once daily since at least 12 months (17.5±2.6/5.2±0.3)<br>NS: self-reported controls<br>confirmation of smoking status: self-reporting	CP according to the new classification of periodontitis	full-mouth SRP using sterile Gracey curettes and an ultrasonic scaler + OHI	full mouth: at six sites of all teeth	6 months	PPD<br>CAL<br>FMPS<br>BOP

**Wan et al. (2009) [91]**	prospective cohort	China	CS: 20 (20/0)<br><br>NS: 20 (20/0)	CS: 46.2±6.8<br><br>NS: 45.0±5.9	CS: smoking habit of ≥10 cigarettes per day for at least 10 years and expressing no interest in quitting smoking in the coming 12 months (20.8±8.7/ NA)<br><br>NS: never having smoked<br><br>confirmation of smoking status: self-reporting	CP: at least 16 standing teeth, with at least one tooth having PPD ≥5 mm in each quadrant, excluding the third molars.	OHI + NSPT, quadrant-wise debridement under local anaesthesia within an 8-week period	full mouth (six sites of each tooth, excluding third molars) and diseased sites (with initial PPD≥5 mm)	12 months	PPD<br>FMPS<br>BOP
***Yadav et al. (2024) [92]**	prospective case-control	India	CS: 27 (20/7)<br><br>NS: 25 (15/10)	CS: 44.37±3.02<br><br>NS: 42.52±5.47	CS: smoking for > 5 years (15.70±3.02/20.08±3.33)<br><br>NS: who had never smoked<br><br>confirmation of smoking status: self-reporting	generalized, stage II-III, Grade B or C periodontitis.	arch-wise SRP was performed at two appointments under local anaesthesia using ultrasonic and hand instruments over two consecutive days.	full mouth (six sites of each tooth, excluding third molars)	3 months	PPD*<br>CAL*<br>PI*<br>GI*<br>GCF*
**Yilmaz et al. (2023) [93]**	prospective cohort	Turkey	CS: 18 (11/7)<br><br>NS: 26 (17/9)	CS: 42.4±NA<br><br>NS: 42.9±NA	CS: smoking ⩾5 cigarettes per day for at least 1 year (16.46±8.68/ 14.66±6.75)<br><br>NS: had never smoked<br><br>confirmation of smoking status: self-reporting	generalized stage III or IV, Grade C periodontitis with at least two bleeding sites with PPD 6-10 mm	SRP conducted with a combination of a piezoelectric ultrasonic device in periodontology mode and gentle use of hand instruments under local anesthesia, which was completed in two consecutive sessions during the same day.	periodontitis sites	12 weeks	PPD<br>CAL<br>FMPS*<br>BOP*
**Zuabi et al. (1999) [94]**	prospective cohort	Israel	CS: 12 (NA/NA)<br><br>NS: 14 (NA/NA)	CS: 41.9±2.4<br><br>NS: 46.1±1.1	CS: smoker (22.8±13.0/19.6±11.1)<br><br>NS: non-smoker<br><br>confirmation of smoking status: self-reporting	established periodontitis (CAL ≥6 mm in two or more sites and with PPD ≥5 mm in another one or more sites)	SRP	full mouth (six sites of each tooth, excluding third molars)	NA	PPD<br>CAL<br>PI<br>GI
