# Supplementary material for: Impact of Traditional and Alternative Nicotine-Containing Product Use on Non-Surgical Periodontal Therapy: A Systematic Review and Network Meta-Analysis
Source: Dent J (Basel). 2026 Sep 22;14(10):616. doi: 10.3390/dj14100616 (PMC13703454; doi:10.3390/dj14100616)
Supplement: Supplementary file 1 [file dentistry-14-00616-s001.zip › Table S2 (List of exluded articles at full-text selection).pdf]

Author	Title	Reason for exclusion
**Abreu et al. (2019) [30]**	*Frequency of Porphyromonas gingivalis fimA in smokers and nonsmokers after periodontal therapy.*	included
**Agarwal et al. (2012)**	*Efficacy of local drug delivery of 0.5% clarithromycin gel as an adjunct to non-surgical periodontal therapy in the treatment of current smokers with chronic periodontitis: a randomized controlled clinical trial*	no non-smokers
**Ah et al. (1994)**	*The effect of smoking on the response to periodontal therapy*	surgical treatment
**Akpinar et al. (2013) [31]**	*The effects of non-surgical periodontal therapy on oxidant and anti-oxidant status in smokers with chronic periodontitis*	included
**AlAhmari et al. (2019) [35]**	*Effectiveness of scaling and root planing with and without adjunct antimicrobial photodynamic therapy in the treatment of chronic periodontitis among cigarette-smokers and never-smokers: A randomized controlled clinical trial*	included
**Al-Hamoudi et al. (2020) [32]**	*Effect of scaling and root planing on the expression of anti-inflammatory cytokines (IL-4, IL-9, IL-10, and IL-13) in the gingival crevicular fluid of electronic cigarette users and non-smokers with moderate chronic periodontitis*	included
**Alharthi et al. (2019)**	*Impact of cigarette smoking and vaping on the outcome of full-mouth ultrasonic scaling among patients with gingival inflammation: a prospective study*	no periodontitis

**Al Hulami et al. (2011)**	*The effect of locally delivered doxycycline as an adjunctive therapy to scaling and root planing in smokers*	no non-smokers
**Alhumaidan et al. (2022)**	*Comparison of Whole Salivary Cortisol and Interleukin 1-Beta Levels in Light Cigarette-Smokers and Users of Electronic Nicotine Delivery Systems before and after Non-Surgical Periodontal Therapy*	no periodontitis
**Alkhalifah et al. (2024)**	*Effect of scaling on levels of interleukin 1-beta and clinical periodontal parameters among e-cigarette users and non-smokers: A prospective study*	no periodontitis
**Al-Kheraif et al. (2022) [33]**	*Antimicrobial photodynamic therapy using chloro-aluminum phthalocyanine for treating advanced stage-III periodontitis in smoking patients*	included
**Al-Kheraif et al. (2022) [34]**	*Chloro-aluminum phthalocyanine-mediated photodynamic therapy in the treatment of stage-II chronic periodontitis among smokers*	included
**Alqahtani et al. (2022)**	*Comparative assessment of periodontal treatment needs among the electronic cigarette users and traditional smokers*	no periodontal therapy
**Al-Rabiah et al. (2020)**	*Efficacy of Scaling and Root Planing with Photobiomodulation for Treating Periodontitis in Gutka Chewers: A Randomized Controlled Trial*	no non-smokers
**Al-Shammari et al. (2001)**	*Effect of non-surgical periodontal therapy on C-telopeptide pyridinoline cross-links (ICTP) and interleukin-1 levels*	no separate smoking groups
**Angst et al. (2019)**	*Supportive periodontal therapy in moderate-to-severe periodontitis patients: A two-year randomized clinical trial*	no separate smoking groups

**Angst et al. (2013)**	*Response of molars and non-molars to a strict supragingival control in periodontal patients*	results for molars and non molars
**Angst et al. (2013)**	*Response of molars and non-molars to a strict supragingival control in periodontal patients*	wrong outcome measurement
**Apatzidou et al. (2005) [36]**	*Impact of smoking on the clinical, microbiological and immunological parameters of adult patients with periodontitis*	included
**Arias-Bujanda et al. (2020)**	*Diagnostic accuracy of IL1β in saliva: The development of predictive models for estimating the probability of the occurrence of periodontitis in non-smokers and smokers*	no clinical parameters
**Ardais et al. (2014) [37]**	*The effect of smoking on bleeding on probing after nonsurgical periodontal therapy: a quasi-experimental study*	included
**Arikan et al. (2023) [38]**	*Clinical and Biochemical Effects of Smoking on Non-Surgical Periodontal Treatment in Grade III Stage C Periodontitis Patients*	included
**Aziz et al. (2015) [39]**	*Effectiveness of scaling and root planning (SRP) on periodontal parameters and systemic inflammatory markers in smokers with chronic periodontitis*	included
**Bergamaschi et al. (2016)**	*Full mouth periodontal debridement with or without adjunctive metronidazole gel in smoking patients with chronic periodontitis: A pilot study*	no non-smokers
**Boulaamaim et al. (2020)**	*Selective Influence of Smoking on Periodontal Treatment Outcomes after 3 Years of Follow-up*	wrong population

**Buduneli et al. (2009) [40]**	*Interleukin-17, RANKL, and osteoprotegerin levels in gingival crevicular fluid from smoking and non-smoking patients with chronic periodontitis during initial periodontal treatment*	included
**Bunaes et al. (2015)**	*Site-specific treatment outcome in smokers following non-surgical and surgical periodontal therapy*	wrong population
**Camargo et al. (2016) [41]**	*Prevalence of periodontopathogens and Candida spp. in smokers after nonsurgical periodontal therapy - a pilot study*	included
**Camargo et al. (2016)**	*Prevalence of periodontopathogens and Candida spp. in smokers after nonsurgical periodontal therapy - a pilot study*	duplicate
**Chandra et al. (2011) [42]**	*The comparative evaluation of xanthan gel with chlorhexidine (Chlosite) in smokers and non-smokers: A clinical and microbiological assessment*	included
**Chandra et al. (2012) [43]**	*Efficacy of lycopene as a locally delivered gel in the treatment of chronic periodontitis: smokers vs nonsmokers*	included
**Citterio et al. (2024)**	*Pocket closure in stage III–IV grade C periodontitis: A multilevel analysis following non-surgical periodontal therapy*	smoking OR
**Ciurescu et al. (2019)**	*Adjunctive use of InGaAsP and Er,Cr:YSGG lasers in nonsurgical periodontal therapy: a randomized controlled clinical study*	no separate smoking groups
**Cmok Kucic et al. (2022)**	*Minimally invasive non-surgical therapy (MINST) in patients with advanced periodontitis: a 6-month results of a split-mouth, randomized controlled clinical trial. Part 1: clinical outcomes*	smoking OR

**Ctri (2018)**	*The effect of teeth cleaning and indocyanine green dye and laser therapy on levels of fluid inside the gums in subjects with gum disease who are smokers versus nonsmokers – a comparative study*	clinical trial
**Ctri (2019)**	*To measure bone marker levels among smokers with chronic periodontitis before and after periodontal treatment*	clinical trial
**Ctri (2016)**	*A clinical trial to evaluate and compare the level of nitric oxide in serum and saliva of smokers and non smokers with periodontal disease before and after oral hygiene measures (professional teeth cleaning)*	clinical trial
**Ctri (2017)**	*Evaluation and comparison of the efficacy of locally delivered ocimum sanctum gel and 8-Hydroxydeoxyguanosine levels in gingival crevicular fluid in subjects with chronic periodontitis before and after scaling and root planning therapy*	clinical trial
**Ctri et al. (2019)**	*To compare a substance in gum fluids of smokers and non-smokers with gum disease before and after treatment with and without red light therapy*	clinical trial
**Ctri et al. (2020)**	*The advantage of lycopene capsule over cleaning and polishing in smokers having gum problems*	clinical trial
**Ctri et al. (2019)**	*To measure bone marker levels among smokers with chronic periodontitis before and after periodontal treatment*	clinical trial
**Ctri et al. (2019)**	*To compare a substance in gum fluids of smokers and non-smokers with gum disease before and after treatment with and without red light therapy*	clinical trial

**Ctri et al. (2016)**	*A clinical trial to evaluate and compare the level of nitric oxide in serum and saliva of smokers and non smokers with periodontal disease before and after oral hygiene measures (professional teeth cleaning)*	clinical trial
**Ctri et al. (2018)**	*The effect of teeth cleaning and indocyanine green dye and laser therapy on levels of fluid inside the gums in subjects with gum disease who are smokers versus nonsmokers – a comparative study*	clinical trial
**Ctri et al. (2017)**	*Evaluation and comparison of the efficacy of locally delivered ocimum sanctum gel and 8-Hydroxydeoxyguanosine levels in gingival crevicular fluid in subjects with chronic periodontitis before and after scaling and root planning therapy*	clinical trial
**Ctri et al. (2020)**	*The advantage of lycopene capsule over cleaning and polishing in smokers having gum problems*	clinical trial
**D'Aiuto et al. (2006)**	*Periodontal infections cause changes in traditional and novel cardiovascular risk factors: results from a randomized controlled clinical trial*	smoking OR
**D'Aiuto et al. (2005) [44]**	*Relative contribution of patient-, tooth-, and site-associated variability on the clinical outcomes of subgingival debridement. I. Probing depths*	included
**Darby et al (2023)**	*The effects of smoking on clinical and microbiological parameters following scaling and root planing in chronic (CP) and generalised aggressive periodontitis (GAgP) patients*	no article found
**Darby et al. (2005) [45]**	*Clinical and microbiological effect of scaling and root planing in smoker and non-smoker chronic and aggressive periodontitis patients*	included

**Dede et al. (2023)**	*Six miRNA expressions in the saliva of smokers and non-smokers with periodontal disease*	duplicate
**de Melo Soares et al. (2019)**	*Antimicrobial photodynamic therapy as adjunct to non-surgical periodontal treatment in smokers: a randomized clinical trial*	no non-smokers
**Dodington et al. (2015)**	*Higher Intakes of Fruits and Vegetables, β-Carotene, Vitamin C, α-Tocopherol, EPA, and DHA Are Positively Associated with Periodontal Healing after Nonsurgical Periodontal Therapy in Nonsmokers but Not in Smokers*	wrong population, diabetes
**Dodington et al. (2021)**	*Improved Healing after Non-Surgical Periodontal Therapy Is Associated with Higher Protein Intake in Patients Who Are Non-Smokers*	wrong population, comorbidities
**Do et al. (2023) [46]**	*The influence of smoking on oral neutrophils and matrix metalloproteinase-8 in periodontitis patients before and after nonsurgical treatment*	included
**Dosunmu et al. (2015)**	*Smokers and Non Smokers: A Comparison of Oral Health Practices and Effect of Non Surgical Periodontal Therapy on their Periodontium*	no periodontitis
**Eggert et al. (2001)**	*Effects of smoking and treatment status on periodontal bacteria: evidence that smoking influences control of periodontal bacteria at the mucosal surface of the gingival crevice*	no periodontal therapy
**Eltas et al. (2012) [47]**	*Clinical effects of Nd:YAG laser applications during nonsurgical periodontal treatment in smoking and nonsmoking patients with chronic periodontitis*	included
**Erdemir et al. (2007) [48]**	*Effect of smoking on folic acid and vitamin B12 after nonsurgical periodontal intervention*	included

**Erdemir et al. (2004) [49]**	*Effects of smoking on clinical parameters and the gingival crevicular fluid levels of IL-6 and TNF-alpha in patients with chronic periodontitis*	included
**Euctr (2015)**	*Comparison of the efficacy of treatment of chronic periodontitis with scaling and root-planning alone or in combination with azithromycin – a prospective, double blind, randomised clinical trial*	clinical trial
**Ezber et al. (2023)**	*Different application procedures of Nd:YAG laser as an adjunct to scaling and root planning in smokers with stage III grade C periodontitis: a single-blind, randomized controlled trial*	no non-smokers
**Farina et al. (2010)**	*Effect of smoking status on pocket probing depth and bleeding on probing following non-surgical periodontal therapy*	no full text
**Faveri et al. (2014)**	*Clinical and microbiologic effects of adjunctive metronidazole plus amoxicillin in the treatment of generalized chronic periodontitis: smokers versus non-smokers*	adjunct therapy
**Feres et al. (2015) [50]**	*Subgingival bacterial recolonization after scaling and root planing in smokers with chronic periodontitis*	included
**Fiorini et al. (2015)**	*Smoking Cessation May Promote Additional Benefits to Non-Surgical Periodontal Therapy in Adults With Chronic Periodontitis*	wrong publication type
**Fullmer et al. (2009)**	*Smoking cessation alters subgingival microbial recolonization*	wrong population

**Hughes et al. (2006)**	*Prognostic factors in the treatment of generalized aggressive periodontitis: II. Effects of smoking on initial outcome.*	wrong population: generalised aggressive periodontitis
**Gaind et al. (2024) [51]**	*Evaluation of Glycosylated Hemoglobin Levels and Effect of Tobacco Smoking in Periodontally Diseased Non-Diabetic Patients*	included
**Gandhi et al. (2024)**	*Comparative Evaluation of Ketoprofen Gel Alone and in Combination with Doxycycline Gel as Local Drug Delivery in the Treatment of Chronic Periodontitis among Smokers and Non-Smokers: A Randomized Clinical Trial*	adjunct therapy
**Ghallab et al (2010) [52]**	*Salivary-soluble CD44 levels in smokers and non-smokers with chronic periodontitis: a pilot study*	included
**Ghazal et al. (2023)**	*A placebo-controlled randomized clinical trial of antibiotics versus probiotics as an adjuvant to nonsurgical periodontal treatment among smokers with Stage III, Grade C generalized periodontitis*	adjunct therapy
**Gomes et al. (2008)**	*The effect of a supragingival plaque-control regimen on the subgingival microbiota in smokers and never-smokers: evaluation by real-time polymerase chain reaction*	wrong outcome measurement
**Gomes et al. (2009)**	*The effect of smoking on gingival crevicular fluid volume during the treatment of gingivitis*	no periodontitis
**Gomes et al. (2007)**	*Effect of supragingival plaque control in smokers and never-smokers: 6-month evaluation of patients with periodontitis*	wrong outcome measurement

**Goodson et al. (2012)**	*Control of periodontal infections: a randomized controlled trial I. The primary outcome attachment gain and pocket depth reduction at treated sites*	no separate smoking groups
**Grossi et al. (2007)**	*Mechanical therapy with adjunctive minocycline microspheres reduces red-complex bacteria in smokers*	no clinical parameters
**Grossi et al. (2006)**	*Subset analysis by smoking status with Arestin® in periodontitis therapy*	conference abstract
**Grossi et al. (1997) [53]**	*Effects of smoking and smoking cessation on healing after mechanical periodontal therapy*	included
**Guentsch et al. (2008) [54]**	*Lipid peroxidation and antioxidant activity in saliva of periodontitis patients: effect of smoking and periodontal treatment*	included
**Gugnani et al (2023)**	*Does smoking exposure alter the effect of non-surgical periodontal therapy?*	wrong publication type: comment
**Guru et al. (2018)**	*Comparative evaluation of salivary hepatocyte growth factor in smokers and non-smokers with chronic periodontitis*	included
**Hadžić et al. (2023) [56]**	*Oxidative stress and C-reactive protein as salivary biomarkers in smokers with periodontitis stage III and IV after non-surgical periodontal therapy (a pilot study)*	included
**Haffajee et al. (1997)**	*The effect of SRP on the clinical and microbiological parameters of periodontal diseases*	no separate smoking groups
**Hendek et al. (2015) [57]**	*Effect of initial periodontal therapy on oxidative stress markers in gingival crevicular fluid, saliva, and serum in smokers and non-smokers with chronic periodontitis*	included
**Hendek et al. (2020) [59]**	*The effect of smoking on gingival crevicular fluid peptidoglycan recognition protein-1 level following initial periodontal therapy in chronic periodontitis*	included
**Hussein et al. (2021)**	*Salivary Levels of Interleukin-1beta, Tumour Necrosis Factor-α, and C-Reactive Proteins in Smokers Patients with Severe Chronic Periodontitis*	no periodontal therapy

**Ikezawa-Suzuki et al. (2008)**	*Effects of treatment on soluble tumour necrosis factor receptor type 1 and 2 in chronic periodontitis*	wrong population
**Ikhar et al. (2024) [58]**	*Comparative evaluation of semaphorin-4D, peptidylarginine deiminase-2, and matrix metalloproteinase-8 levels of gingival crevicular fluid in periodontally healthy and Stage III periodontitis smoker and non-smoker patients before and after non-surgical periodontal therapy*	included
**Jeffcoat et al. (2000)**	*Use of a biodegradable chlorhexidine chip in the treatment of adult periodontitis: clinical and radiographic findings*	no separate smoking groups
**Jin et al (2000)**	*Comparison of treatment response patterns following scaling and root planing in smokers and non-smokers with untreated adult periodontitis*	*full text requested*
**Johnson et al. (2004)**	*Cigarette smoking and the periodontal patient*	wrong publication type: review
**Kakade et al. (2022)**	*Effect of non-surgical periodontal therapy (NSPT) on salivary glutathione reductase (GR) in smokers and periodontitis subjects*	wrong population: former smokers in nonsmoker group
**Kaldahl et al. (1996)**	*Levels of cigarette consumption and response to periodontal therapy*	adjunct therapy
**Kamma et al. (2007)**	*Adjunctive diode laser treatment on clinical parameters of chronic periodontitis*	no article found
**Kanmaz et al. (2019)**	*Microbiological and biochemical findings in relation to clinical periodontal status in active smokers, non-smokers and passive smokers*	no periodontal therapy
**Kanmaz et al. (2020)**	*Effects of smoking on non-surgical periodontal therapy in patients with periodontitis Stage III or IV, and Grade C*	wrong population
**Karsiyaka Hendek et al. (2020) [59]**	*The effect of smoking on gingival crevicular fluid peptidoglycan recognition protein-1 level following initial periodontal therapy in chronic periodontitis*	included
**Kerdvongbundit et al. (2003)**	*Effect of triclosan on healing following non-surgical periodontal therapy in smokers*	no non-smokers
**Keshavarzi et al. (2013)**	*Comparing the level of salivary soluble CD44 in smokers and non-smokers with chronic moderate periodontitis before and after treatment*	no full text
**Khairnar et al. (2013)**	*Evaluation of Calendula officinalis as an anti-plaque and anti-gingivitis agent*	no periodontitis

**Kilinc et al. (2018)**	*Effect of diode laser decontamination as an adjunct to nonsurgical periodontal therapy on the clinical and biochemical parameters in smokers and non-smokers with chronic periodontitis*	conference abstract
**Kinane et al. (1997)**	*The effect of smoking on mechanical and antimicrobial periodontal therapy*	adjunct therapy
**Klok et al. (2019)**	*Salivary IL-1ß but not dietary flavonoids are associated with healing after periodontal therapy to prevent tooth loss*	conference abstract
**Knie et al. (2022)**	*Serum biomarker profiling in smokers and non-smokers following periodontal therapy*	wrong population
**Krohn-Dale et al. (2012)**	*Er:YAG laser in the treatment of periodontal sites with recurring chronic inflammation: a 12-month randomized, controlled clinical trial*	no non-smokers
**Kučič et al. (2023)**	*Minimally invasive non-surgical therapy (MINST) in stage III periodontitis patients: 6-month results of a split-mouth, randomised controlled clinical trial*	smoking OR
**Kumari et al. (2017)**	*Subgingivally delivered 1.2% atorvastatin in the treatment of chronic periodontitis among smokers: a randomized, controlled clinical trial*	no non-smokers
**Kumar et al. (2024)**	*Evaluating the Effect of Low-Level Laser Therapy as an Adjunct to Non-Surgical Periodontal Therapy on Salivary Interleukin-1ß Levels in Chronic Periodontitis*	wrong population
**Kumar et al. (2023)**	*Does e-cigarette use affect response to non-surgical periodontal therapy?*	wrong publication type: comment
**Kurtiş et al. (2007)**	*Gingival crevicular fluid prostaglandin E(2) and thiobarbituric acid reactive substance levels in smokers and non-smokers with chronic periodontitis following phase I periodontal therapy and adjunctive use of flurbiprofen*	wrong study duration
**Kurtis et al. (2007)**	*GCF MMP-8 levels in smokers and non-smokers with chronic periodontitis following scaling and root planing accompanied by systemic use of flurbiprofen*	wrong study duration
**Kwan Yat et al. (2016)**	*Adjunctive use of modified Yunu-Jian in the non-surgical treatment of male smokers with chronic periodontitis: a randomized double-blind, placebo-controlled clinical trial*	no non-smokers
**Lee et al. (2021)**	*Salivary Pro-Inflammatory Markers and Smoking Status Influences the Treatment Effectiveness of Periodontal Disease Patients with Hypertension*	wrong population
**Leite et al. (2023)**	*Salivary cytokine expression after non-surgical periodontal therapy in smokers: 12-month follow-up*	no non-smokers
**Leppilahti et al. (2014)**	*Gingival crevicular fluid matrix metalloproteinase-8 levels predict treatment outcome among smokers with chronic periodontitis*	background; wrong outcome measurement
**Leppilahti et al. (2015)**	*The utility of gingival crevicular fluid matrix metalloproteinase-8 response patterns in prediction of site-level clinical treatment outcome*	wrong population

**Levine et al. (2017)**	*Low Biofilm Lysine Content in Refractory Chronic Periodontitis*	adjunct therapy
**Levin et al. (2011)**	*Association of self-perceived periodontal status with oral hygiene, probing depth and alveolar bone level among young adults*	no periodontal therapy
**Levin et al. (2018)**	*Demographic profile, Oral Health Impact Profile and Dental Anxiety Scale in patients with chronic periodontitis: a case-control study*	no periodontal therapy
**Liu et al. (2021)**	*Prognostic factors affecting the short-term efficacy of non-surgical treatment of chronic periodontitis: a multilevel modeling analysis*	no separate smoker groups
**L. M. Shaddox et al. (2007)**	*Microbiologic changes following administration of locally delivered doxycycline in smokers: a 15-month follow-up*	no non-smokers
**Lütfioglu et al. (2021) [60]**	*Effects of smoking on the gingival crevicular fluid levels of interleukin-17A, interleukin-17E, and oxidative stress following periodontal treatment process*	included
**Machion et al. (2004)**	*Locally delivered doxycycline as an adjunctive therapy to scaling and root planing in the treatment of smokers: A clinical study*	no non-smokers
**Machion et al. (2006)**	*Locally delivered doxycycline as an adjunctive therapy to scaling and root planing in the treatment of smokers: a 2-year follow-up*	no non-smokers
**Mantyla et al. (2006) [61]**	*Monitoring periodontal disease status in smokers and nonsmokers using a gingival crevicular fluid matrix metalloproteinase-8-specific chair-side test*	included
**Maged et al. (2022) [29]**	*Impact of smoking on gingival crevicular fluid and salivary periostin levels in stage II–III periodontitis patients following non-surgical periodontal therapy*	included
**Mashalkar et al. (2024) [62]**	*Comparative Analysis of Salivary Glutathione Reductase Levels in Smokers and Non-Smokers with Periodontitis Following Non-Surgical Periodontal Therapy*	included
**Maske et al. (2023) [63]**	*Evaluation of efficacy of locally delivered Herbal extract in treatment of Chronic periodontitis – A Randomised controlled trial*	included
**Matarazzo et al. (2008)**	*Clinical and microbiological benefits of systemic metronidazole and amoxicillin in the treatment of smokers with chronic periodontitis: a randomized placebo-controlled study*	no non-smokers
**Meşeli et al. (2022)**	*Pocket closure after non-surgical periodontal treatment in smoker and non-smoker patients with stage II periodontitis*	conference abstract
**Meenawat et al. (2015) [64]**	*Evaluation of the effect of nicotine and metabolites on the periodontal status and the mRNA expression of interleukin-1β in smokers with chronic periodontitis*	included
**Meulman et al. (2013)**	*One stage, full-mouth, ultrasonic debridement in the treatment of severe chronic periodontitis in smokers: a preliminary, blind and randomized clinical trial*	wrong population

**Mittal et al. (2018) [65]**	*Effect of Smoking and Tobacco Chewing on Periodontal Disease and Non-Surgical Treatment Outcome: A Clinical and Biochemical Study*	included
**Mohan et al. (2019)**	*The effect of nonsurgical periodontal therapy on pentraxin 3 levels in smokers and nonsmokers with chronic periodontitis*	wrong study duration
**Morello et al. (2002)**	*Clinical response of smokers with a periodontal condition to progressive scaling*	no non-smokers
**Morello et al. (2003)**	*Specific site analysis of CAL and PD in smokers and non-smokers with periodontitis before and after progressive scaling*	conference abstract
**Morello et al. (2005)**	*Effects of azytromycine together with root scaling and planning on smokers and non-smokers with chronic periodontitis*	adjunct therapy
**Mouzakiti et al. (2012) [66]**	*Expression of MMPs and TIMP-1 in smoker and nonsmoker chronic periodontitis patients before and after periodontal treatment*	included
**Nagarajan et al. (2020)**	*Comparative analysis of oral microbial flora of smokers and non-smokers with periodontitis*	no clinical parameters
**Nakayama et al. (2020)**	*A multicenter prospective cohort study on the effect of smoking cessation on periodontal therapies in Japan*	systemic disease
**Nascimento et al. (2020)**	*Periodontitis phenotypes and clinical response patterns to non-surgical periodontal therapy: reflections on the new periodontitis classification*	no separate smoking groups
**Nassrawin et al. (2010)**	*Effect of smoking on the response to nonsurgical periodontal therapy*	no periodontitis
**NCT et al. (2014)**	*1% Alendronate Gel in Treatment of Chronic Periodontitis Among Smokers*	clinical trial
**NCT et al. (2016)**	*Effect of Tooth Cleaning in Smokers and Non Smokers With Gum Disease*	clinical trial
**NCT et al. (2017)**	*Adjunct Photodynamic Therapy in Chronic Periodontitis Among Cigarette-smokers*	clinical trial
**NCT et al. (2017)**	*Evaluation of Antimicrobial Photodynamic Therapy as an Adjunct to Periodontal Treatment in Smokers*	clinical trial
**NCT et al. (2023)**	*The Effect of Smoking and Chlorhexidine on Periodontal Tissues After Non-Surgical Periodontal Therapy in Patients With Periodontitis*	clinical trial
**NCT et al. (2021)**	*The Influence of Smoking in Periodontitis Patients, Before and After Nonsurgical Treatment*	clinical trial
**NCT et al. (2022)**	*Effect of Smoking on Periodontal Therapy in Periodontitis*	clinical trial
**NCT et al. (2023)**	*Effect of Non-Surgical Periodontal Treatment on Biomarker Levels*	clinical trial
**NCT et al. (2024)**	*Assessment the Efficacy of Curcumin Local Delivery Gel in Treatment of Periodontitis in Smokers: RCT*	clinical trial

**NCT et al. (2023)**	*Effect of Smoking Cessation on Salivary IL-1β and PGE-2 Levels Following Non-Surgical Periodontal Therapy*	clinical trial
**NCT et al. (2023)**	*The Effect of Scaling and Root Planing on Oral Inflammatory Biomarkers Among E-cig Users and Non-smokers*	clinical trial
**NCT et al. (2020)**	*Effect of Smoking and Periodontal Therapy on Salivary and Gingival Crevicular IL-17 and IL-35*	clinical trial
**NCT et al. (2022)**	*The Effects of Smoking on miRNA-223 Before–After Non-Surgical Periodontal Therapy*	clinical trial
**NCT et al. (2003)**	*Effect of Three Periodontal Therapies in Current Smokers and Non-Smokers*	clinical trial
**NCT et al. (2013)**	*MTZ Plus AMX in the Treatment of Smokers and Non-smokers*	clinical trial
**Needleman et al. (2005)**	*Low-dose doxycycline for periodontitis in smokers*	conference abstract
**Öngöz Dede et al. (2023) [67]**	*Six miRNA expressions in the saliva of smokers and non-smokers with periodontal disease*	included
**Nwhator et al. (2009)**	*Do smokers benefit from dental hygiene oral prophylaxis? A Nigerian pilot study*	wrong outcome measurement
**Oringer et al. (2002)**	*The challenge of treating periodontal patients who smoke – the efficacy of Arestin*	no non-smokers
**Ozdemir et al. (2021) [68]**	*Photobiomodulation Therapy by 820 nm Diode Laser on Nonsurgical Periodontal Treatment of Smoker and Nonsmoker Patients: a Single-Blind Parallel Randomized Clinical Trial*	included
**Ozener et al. (2020)**	*Efficacy of Hyaluronic Acid Gel as an Adjunct to Non-Surgical Periodontal Treatment in Smokers with Periodontitis: A Retrospective Case Control Study*	no non-smokers
**Padhye et al. (2019) [69]**	*Quantification and Comparison of the Impact of the Smoking Status on Oral Polymorphonuclear Leukocyte and Malondialdehyde Levels in Individuals with Chronic Periodontitis: A Double-Blinded Longitudinal Interventional Study*	included
**Pahkla et al. (2006)**	*The efficacy of non-surgical and systemic antibiotic treatment on smoking and non-smoking periodontitis patients*	adjunct therapy
**Palmer et al. (1998)**	*Effect of smoking on non-surgical periodontal treatment*	article not found
**Palmer et al. (1999) [70]**	*Non-surgical periodontal treatment with and without adjunctive metronidazole in smokers and non-smokers*	included
**Palmer et al. (1998)**	*Adjunctive systemic and locally delivered metronidazole in the treatment of periodontitis: a controlled clinical study*	no full text

**Pamuk et al. (2023)**	*Impact of low-level laser therapy as an adjunct to non-surgical periodontal treatment on the levels of tissue plasminogen activator and plasminogen activator inhibitor 1 in Stage 3–4, Grade C periodontitis patients: a split-mouth, randomized control study*	no smokers; wrong population
**Pamuk et al. (2017) [71]**	*The effect of low-level laser therapy as an adjunct to non-surgical periodontal treatment on gingival crevicular fluid levels of transforming growth factor-beta 1, tissue plasminogen activator and plasminogen activator inhibitor 1 in smoking and non-smoking chronic periodontitis patients: A split mouth randomized control study.*	included
**Papantonopoulos et al. (1999)**	*Smoking influences decision making in periodontal therapy: a retrospective clinical study*	wrong outcome measurement
**Papantonopoulos et al. (2004)**	*Effect of periodontal therapy in smokers and non-smokers with advanced periodontal disease: results after maintenance therapy for a minimum of 5 years*	wrong outcome measurement
**Paquette et al. (2004)**	*Clinical relevance of adjunctive minocycline microspheres in patients with chronic periodontitis: secondary analysis of a phase 3 trial*	no separate smoking groups
**Pouly et al. (2022)**	*Effect of switching from cigarette smoking to the use of the tobacco heating system on periodontitis treatment outcome: Periodontal parameter results from a multicenter Japanese study*	background; no non-smokers
**Pradeep et al. (2013)**	*Local drug delivery of 0.5% azithromycin in the treatment of chronic periodontitis among smokers*	no non-smokers
**Preber et al. (1990)**	*Effect of cigarette smoking on periodontal healing following surgical therapy*	surgical treatment
**Preber et al. (1986) [72]**	*Effect of non-surgical treatment on gingival bleeding in smokers and non-smokers*	included
**Preber et al. (1986) [73]**	*The effect of non-surgical treatment on periodontal pockets in smokers and non-smokers*	included
**Preber et al. (1995) [74]**	*Periodontal healing and periopathogenic microflora in smokers and non-smokers*	included
**Preshaw et al. (2005) [75]**	*The effect of quitting smoking on chronic periodontitis*	included
**Preshaw et al. (2005)**	*Periodontal maintenance in a specialist periodontal clinic and in general dental practice*	no separate smoking groups
**Preshaw et al. (2005)**	*Adjunctive subantimicrobial dose doxycycline in smokers and non-smokers with chronic periodontitis*	wrong publication type

**Preshaw et al. (2013)**	*Outcomes of non-surgical periodontal treatment by dental hygienists in training: impact of site- and patient-level factors*	systemic disease
**Preshaw et al. (1999) [76]**	*Progression and treatment of chronic adult periodontitis*	included but results as only figures
**Preus et al. (2014)**	*Baseline adjustment and change revisited: effect of smoking on change in periodontal status following periodontal therapy*	adjunct therapy
**Pucher et al. (1997) [77]**	*Results of limited initial periodontal therapy in smokers and non-smokers*	included
**Raut et al. (2019)**	*Subgingivally delivered coenzyme Q10 in the treatment of chronic periodontitis among smokers: A randomized, controlled clinical study*	no non-smokers
**Ravidà et al. (2020)**	*Dose-dependent effect of smoking and smoking cessation on periodontitis-related tooth loss during 10–47 years periodontal maintenance—A retrospective study in compliant cohort*	no clinical parameters
**Renvert et al. (1998) [78]**	*The clinical and microbiological effects of non-surgical periodontal therapy in smokers and non-smokers*	included
**Rosa et al. (2011) [79]**	*A prospective 12-month study of the effect of smoking cessation on periodontal clinical parameters*	included
**Rosa et al. (2014)**	*Effect of smoking cessation on non-surgical periodontal therapy: results after 24 months*	wrong study duration
**Rosa et al. (2008)**	*Cigarette smoking and alveolar bone in young adults: a study using digitized radiographs*	no periodontitis
**Ryan et al. (2006)**	*Periodontal therapy effects on biomarkers of systemic inflammation in smokers*	no non-smokers
**Ryder et al. (1999) [80]**	*Effects of smoking on local delivery of controlled-release doxycycline as compared to scaling and root planing*	included
**Salhi et al. (2020) [81]**	*Respective Effects of Oral Hygiene Instructions and Periodontal Nonsurgical Treatment (Debridement) on Clinical Parameters and Patient-Reported Outcome Measures with Respect to Smoking*	included
**Salhi et al. (2022)**	*Predicting probing depth reduction after periodontal non-surgical treatment in smokers according to the nicotine dependence and the number of cigarette consumed*	no non-smokers
**Sandhya et al. (2011)**	*Comparative evaluation of the efficacy of 10% doxycycline hyclate in the periodontal treatment of smokers – a clinical and microbiological study*	no non-smokers
**Savran et al. (2024) [82]**	*Clinical effects of omega-3 fatty acids supplementation in the periodontal treatment of smokers and non-smokers with periodontitis: a retrospective study*	included
**Shah et al. (2023) [17]**	*Retrospective exploratory study of smoking status and e-cigarette use with response to non-surgical periodontal therapy*	included

**Sheokand et al (2019) [83]**	*The comparative evaluation of 1% alendronate gel as local drug delivery system in chronic periodontitis in smokers and non smokers: Randomized clinical trial*	included
**Shiloah et al. (2014)**	*The effect of long-term aspirin intake on the outcome of non-surgical periodontal therapy in smokers: a double-blind, randomized pilot study*	no non-smokers
**Show et al. (2022)**	*Effect of nonsurgical periodontal therapy and smoking status on hematological variables related to anemia of chronic disease in chronic periodontitis patient: a case-control study*	no clinical parameters
**Singh et al. (2021)**	*Evaluation of locally-administered controlled-release doxycycline hyclate gel in smokers and non-smokers in the management of periodontitis: An Indian study*	adjunct therapy
**Soder et al. (1999)**	*Longitudinal effect of non-surgical treatment and systemic metronidazole for 1 week in smokers and non-smokers with refractory periodontitis: A 5-year study*	wrong outcome measurement
**SS AlHarti et al. (2019)**	*Impact of cigarette smoking and vaping on the outcome of full-mouth ultrasonic scaling among patients with gingival inflammation: a prospective study*	no periodontitis
**Stein et al. (2004)**	*Augmented transforming growth factor-beta1 in gingival crevicular fluid of smokers with chronic periodontitis*	no clinical parameters
**Stratul et al. (2011)**	*Effect of smoking on the results of a chlorhexidine digluconate treatment extended up to 3 months after scaling and root planing – a pilot study*	adjunct therapy
**Tamashiro et al. (2023)**	*Smoking-induced subgingival dysbiosis precedes clinical signs of periodontal disease*	no clinical parameters
**Toker et al. (2012) [84]**	*Influence of smoking on interleukin-1beta level, oxidant status and antioxidant status in gingival crevicular fluid from chronic periodontitis patients before and after periodontal treatment*	included
**Tomasi et al. (2007)**	*Factors influencing the outcome of non-surgical periodontal treatment: a multilevel approach*	smoking OR
**Tomasi et al. (2011)**	*Locally delivered doxycycline as an adjunct to mechanical debridement at retreatment of periodontal pockets: outcome at furcation sites*	no separate smoking groups
**Tomasi et al. (2004) [85]**	*Locally delivered doxycycline improves the healing following non-surgical periodontal therapy in smokers*	included
**Türkoğlu et al. (2016) [86]**	*Does smoking affect gingival crevicular fluid LL-37 levels following non-surgical periodontal treatment in chronic periodontitis?*	included
**Čuk et al. (2020)**	*Influence of adjunctive azithromycin on microbiological and clinical outcomes in periodontitis patients: 6-month results of randomized controlled clinical trial*	smoking OR

**Underner et al. (2009)**	*[Effects of smoking on periodontal disease]*	not English language
**Vajawat et al. (2022) [87]**	*Local delivery of hyaluronic acid as an adjunct to scaling and root planing in the treatment of chronic periodontitis in smokers and non-smokers: A clinical and microbiological study*	included
**van Winkelhoff et al. (2001)**	*Smoking affects the subgingival microflora in periodontitis*	no clinical parameters
**Varghese et al. (2020) [88]**	*Salivary 8-hydroxyguanosine levels in smokers and non-smokers with chronic periodontitis*	included
**Vijayakumar et al. (2020) [89]**	*Effect of Nonsurgical Periodontal Therapy on Serum Levels of Interleukin-1β and Interleukin-8 in Smokers and Nonsmokers with Chronic Periodontitis*	included
**Vohra et al. (2020)**	*Effectiveness of scaling and root planing with and without adjunct probiotic therapy in the treatment of chronic periodontitis among shamma users and non-users: A randomized controlled trial*	adjunct tobacco product
**Wadhwa et al. (2023)**	*Levels of nitric oxide in smokers and nonsmokers with chronic periodontitis before and after nonsurgical periodontal treatment*	adjunct tobacco product
**Wan et al. (2009) [91]**	*Effects of smoking on healing response to non-surgical periodontal therapy: a multilevel modelling analysis*	included
**Wilder et al. (2022)**	*Randomized Clinical Trial of a Topical Botanical Patch for the Adjunctive Management of Periodontitis*	no separate smoking groups
**Xu et al. (2003)**	*[The effects of smoking on gingival crevicular fluid volume and elastase before and after initial periodontal treatment]*	not English language
**Yadav et al. (2024) [92]**	*Effect of initial periodontal therapy on metallothionein levels in smokers and non-smokers with periodontitis*	included
**Yilmaz et al. (2023) [93]**	*Baseline interleukin-10, CD163, and tumor necrosis factor-like weak inducer of apoptosis (TWEAK) gingival tissue levels in relation to clinical periodontal treatment outcomes: A 12-week follow-up study*	included
**Özdemir et al. (2021)**	*Photobiomodulation Therapy by 820 nm Diode Laser on Nonsurgical Periodontal Treatment of Smoker and Nonsmoker Patients: A Single-Blind Parallel Randomized Clinical Trial*	duplicate
**Özdoğan et al. (2022)**	*Evaluation of the GCF periostin, IL-17A and IL-17E levels of smoking and non-smoking periodontitis patients received periodontal treatment: Short-term follow-up study*	conference abstract
**Zhao et al. (2005)**	*The clinical effect of full-mouth scaling and root planning on chronic periodontitis: a preliminary report*	not English language

**Zuabi et al. (1999) [94]**	*The effect of smoking and periodontal treatment on salivary composition in patients with established periodontitis*	included

Supplementary Table 2. List of excluded articles at full-text selection with their specific reason
