# Supplementary material for: Impact of Traditional and Alternative Nicotine-Containing Product Use on Non-Surgical Periodontal Therapy: A Systematic Review and Network Meta-Analysis
Source: Dent J (Basel). 2026 Sep 22;14(10):616. doi: 10.3390/dj14100616 (PMC13703454; doi:10.3390/dj14100616)
Supplement: Supplementary file 1 [file dentistry-14-00616-s001.zip › Table S3_Summary of included studies.pdf]

Suppl. Table 3. Summary of included studies; CS, cigarette smokers; EC, e-cigarette users; NS, non-smokers; FS, former smokers; Q, quitters; ST, smokeless tobacco users; BOP, bleeding on probing, PI, plaque index, FMPS, full mouth plaque score; GI, gingival index, PPD, probing pocket depth; GCF, gingival crevicular fluid; MBL, marginal bone loss; SRP, scaling and root planning, RSD, root surface debridement; NSPT, nonsurgical periodontal therapy; CP, chronic periodontitis; RCT, randomized controlled trial; OHI, oral hygiene instructions

*Not included in the quantitative synthesis

Data are shown as mean ± SD if available

Summary of findings								
**Author (year)**	**Study type**	**Smoking status (no. of patients)**	**Examined site**	**Periodontal parameter**	**Baseline value (mean ± SD)**	**Follow up value (mean ± SD)**	**Follow up time**	**Finding**
***Abreu et al. (2019) [30]**	prospective cohort	CS (n=15)	full mouth: six sites per tooth on all the teeth, excluding third molars	PPD	NA	NA	3 months	Comparing CS and NS, at baseline, both groups had a similar periodontal status. There was a statistically significant reduction in the clinical parameters for the NS group, with a decrease in PI (p=0.004) and BOP (p=0.011) indexes, and in PPD (p<0.001) and CAL (p=0.007) measurements, and also for the SM group, with a decrease in PPD (p<0.001) and CAL (p=0.007), comparing baseline with 3 months after periodontal treatment. CS exhibited a higher level of PI (50% -p<0.001) and CAL (5.68 mm – p=0.041) after 3 months than NS (PI: 9.69% and CAL: 3.51 mm).
				CAL	NA	NA		
				BOP	NA	NA		
				FMPS	NA	NA		
		NS (n=16)	full mouth: six sites per tooth on all the teeth, excluding third molars	PPD	NA	NA		
				CAL	NA	NA		
				BOP	NA	NA		
				FMPS	NA	NA		
***Akpinar et al. (2013) [31]**	prospective cohort	CS (n=14)	at least one moderate (4–6 mm) and at least one deep (>6 mm) pockets of teeth per subject	PPD	NA	NA	6 weeks	The periodontal treatment improves the clinical parameters in both smokers and non-smokers. Mean PPD and CAL values were significantly reduced after periodontal treatment in all periodontitis patients (p < 0.05). There were statistically significant differences in PPD values between CS and NS at 6 weeks. PI and GI values at 6 weeks of all periodontitis patients were significantly lower than those at baseline (p < 0.05). There were no significant differences in the PI and GI values between smokers and non-smokers at 6 weeks (p > 0.05). Likewise, there were no significant
				CAL	NA	NA		
				BOP	NA	NA		
				PI	NA	NA		
				GI	NA	NA		

		NS (n=15)	at least one moderate (4–6 mm) and at least one deep (>6 mm) pockets of teeth per subject	PPD	NA	NA		differences in % BOP between smokers and non-smokers ($p > 0.05$) while % BOP values showed significant decreases in response to periodontal treatment in all patients (($p < 0.05$).
				CAL	NA	NA		
				BOP	NA	NA		
				PI	NA	NA		
				GI	NA	NA		
**AlAhmari et al. (2019) (SRP alone) [35]**	RCT	CS (n=22)	full mouth: at 6 sites per tooth on all maxillary and mandibular teeth	PPD	6.10±0.50	5.50±1.20	12 weeks	Outcomes of NSPT for the treatment of CP are compromised in CS compared to NS. At baseline, PI, PD and CAL were comparable among CS compared with NS. BOP was statistically significantly higher among individuals in NS compared with CS. At 3 months follow-up, PI ($P < 0.05$) was statistically significantly higher, and BOP was comparable among CS compared with NS ($P < 0.05$). PPD and CAL ($P < 0.05$) were statistically significantly higher among individuals in CS compared with NS.
				CAL	7.10±0.70	6.00±0.40		
				BOP	35.30±6.90	32.70±2.40		
				FMPS	52.60±9.30	38.50±5.60		
		NS (n=21)	full mouth: at 6 sites per tooth on all maxillary and mandibular teeth	PPD	6.60±0.40	4.10±0.50		
				CAL	7.20±0.50	4.90±0.60		
				BOP	61.20±9.60	20.30±4.70		
				FMPS	57.30±8.40	23.50±4.10		
**Al-Hamoudi et al. (2020) [32]**	prospective cohort	EC (n=36)	full mouth: at 6 sites per tooth excluding third molars	PPD	5.20±0.87	3.70±0.20	3 months	At baseline, there were no differences in PI, PPD, CAL, and GCF between EC users and NS. At the 3-month follow-up, there were no significant differences in PI, GI, PPD, CAL in EC users compared to baseline, while there were significant reductions in PI, GI, and PPD among NS. There was no statistically significant difference in CAL between groups at the 3-month follow-up. At the 3-month follow-up, there were significant reductions in the GCF volume collected from EC users ($P<0.05$) and NS ($P<0.05$) compared with their respective baseline volumes. The GCF volume collected at the 3-month follow-up was significantly higher in EC users than in NS ($P<0.05$).
				CAL	3.40±0.20	3.20±0.10		
				PI	2.62±0.43	1.91±0.18		
				GI	0.36±0.08	0.34±0.06		
				GCF	1.65±0.37	0.66±0.14		
		NS (n=35)	full mouth: at 6 sites per tooth excluding third molars	PPD	5.00±0.06	1.80±0.07		
				CAL	3.10±0.20	2.70±0.08		
				PI	2.25±0.16	0.62±0.14		
				GI	2.55±0.08	0.86±0.10		
				GCF	1.74±0.41	0.18±0.09		
**Al-Kheraif et al. (2022)**	RCT	CS (n=15)	split-mouth design, at 6 sites of the	PPD	6.43±0.51	4.49±0.42	6 months	A significant reduction in PI was observed in both the study groups ($p < .0001$). For BOP, the observed values showed a statistically significant difference between the study groups (CS and NS) at baseline ($p < .05$). For the NS group, BOP reported a significant reduction at 3-and 6-month follow-
				CAL	7.32±1.41	6.52±0.35		
				BOP	35.41±6.82	25.45±7.21		

**(RSD alone) [33]**			tooth on each tooth	FMPS	42.14±10.12	19.41±7.23		ups (p < .05). RSD depicted no significant difference within the CS group at any assessment period. Probing depth and CAL reported statistically significant reductions within both groups and also within the NS and CS (p < .05). An exception was observed for PD values that only reported a significant reduction at the 6-month follow-up in the NSPT subgroup in the CS group (p < .05).
		NS (n=14)	split-mouth design, at 6 sites of the tooth on each tooth	PPD	6.21±0.71	3.43±0.41		
				CAL	5.56±1.03	4.59±0.74		
				BOP	57.25±16.37	20.89±8.31		
				FMPS	39.32±9.21	11.20±2.54		
**Al-Kheraif et al. (2022) (DS alone) [34]**	RCT	CS (n=13)	split-mouth design, at 6 sites of the tooth on each tooth	PPD	4.51±0.77	3.76±0.49	6 months	Plaque scores significantly reduced among both NS and CS (p < .0001). For BOP, a statistically significant difference was noted between CS and NS at baseline (p < .05). BOP was significantly reduced in NS group at 3 and 6 months (p < .05). No statistically significant difference was observed at any time point in CS group. Mean PD and CAL statistically significantly reduced among both NS and CS groups (p < .05), except for PD that only showed significant reduction only at 6 months in the DS subgroup in CS group (p < .05).
				CAL	5.46±1.41	4.62±0.65		
				BOP	34.62±7.99	24.66±7.46		
				FMPS	40.04±13.63	17.83±8.09		
		NS (n=13)	split-mouth design, at 6 sites of the tooth on each tooth	PPD	4.72±0.69	3.03±0.38		
				CAL	5.56±1.03	4.59±0.74		
				BOP	55.27±18.23	18.69±6.51		
				FMPS	38.45±10.34	10.15±4.60		
**Apatzidou et al. (2005) [36]**	prospective cohort	CS (n=15)	1 site/quadrant with the deepest PPD, (not less than 5mm)	PPD	5.90±0.60	3.60±0.50	6 months	At baseline, smokers showed significantly less gingival inflammation and lower GCF volume compared with non-smokers. After treatment, a compromised clinical outcome was noted for smokers in terms of pocket depth reduction and gain in attachment levels. Statistically significant improvements in all clinical indices were found post-operatively in each group (p<0.001). At baseline, the whole-mouth clinical parameters were similar for CS and NS and no significant differences between the two groups were found at reassessement (RAS). At the selected sites a compromised treatment outcome was seen for CS, in terms of PD reduction (p=0.0005) and gain in CAL (p=0.0009). At RAS, smokers presented with higher PD (p=0.0015) and attachment loss (p=0.0350) than NS.
			at 6 sites/tooth;	PPD	4.40±0.70	2.70±0.30		
				CAL	5.30±1.10	4.20±1.10		
				BOP	67.30±16.60	12.10±7.90		
		CS (n=25)	1 site/quadrant with the deepest PPD, (not less than 5mm)	PPD	6.20±0.80	3.10±0.40		
			at 6 sites/tooth;	PPD	4.40±0.60	2.50±0.30		
				CAL	4.90±0.80	3.70±0.80		
				BOP	70.60±18.70	11.60±5.80		
				PPD	3.46±0.47	2.69±0.33	3 months	

**Ardais et al. (2014) [37]**	quasi-experimental study	CS (n=11)	full mouth: at 6 sites per tooth excluding third molars	CAL	4.94±1.53	4.69±1.58		All subjects had significant reductions in mean PPD and percentages of sites with BoP, with no difference between the groups. Only NS presented statistically significant CAL gain. BoP was significantly and consistently reduced at sites with initial PPDs of 1–3 mm and 4–6 mm in both groups. At sites with deep PPD (≥ 7mm), never smokers showed a greater mean reduction in the number of sites with BoP than did smokers (p < 0.05). Thus, periodontal therapy reduced BoP in both groups. However, smoking could negatively affect the BoP reduction at deeper sites after nonsurgical periodontal therapy.
				BOP	8.50±5.95	8.74±4.74		
				FMPS	71.36±17.04	28.53±14.92		
		NS (n=14)	full mouth: at 6 sites per tooth excluding third molars	PPD	3.26±0.44	2.37±0.17		
				CAL	2.95±1.01	2.53±0.97		
				BOP	42.82±16.38	19.14±12.33		
				FMPS	53.2±24.55	34.99±22.46		
***Arikan et al. (2023) [38]**	prospective cohort	CS (n=20)	full mouth: at 6 sites per tooth excluding third molars	PPD*	NA	NA	3 months	At baseline only whole mouth PD and percent of sites with PD>5mm were higher in smokers than non-smoker periodontitis patients (p<.05). All periodontal measurements significantly improved in both periodontitis groups after NSPT (p<.001). After NSPT, the reduction in GI, BOP, and mean PD in sites initially PD≥5mm were lower in smokers than nonsmoker periodontitis patients (p>.05). CS are less responsive to NSPT.
				CAL*	NA	NA		
				PI*	NA	NA		
				BOP*	NA	NA		
				GI*	NA	NA		
		NS (n=20)	full mouth: at 6 sites per tooth excluding third molars	PPD*	NA	NA		
				CAL*	NA	NA		
				PI*	NA	NA		
				BOP*	NA	NA		
				GI*	NA	NA		
**Aziz et al. (2015) [39]**	longitudinal study	CS (n=80)	full mouth: six sites around each tooth	PPD	5.82±0.48	5.63±0.47	3 months	The comparison of baseline clinical measurements between the CS and NS groups indicate a significant difference (P < 0.001 for all) in clinical parameters. Following therapy, the paired t-test showed significantly lowered (P < 0.001 for all) clinical parameters compared to their corresponding baseline values in both groups. The relative % change in clinical parameters with respect to baseline values in CS and NS ranged between 3.31-36.6% and 14.58-43.21%, respectively, and was significantly lower (P < 0.001 for all except GI, P = 0.098) in CS than NS Group.
				CAL	8.45±0.96	8.06±0.94		
				PI	2.98±0.51	2.32±0.53		
				GI	1.83±0.45	1.15±0.30		
		NS (n=51)	full mouth: six sites around each tooth	PPD	5.41±0.45	4.65±0.51		
				CAL	7.63±0.90	6.10±1.20		
				PI	2.35±0.55	1.39±0.49		
				GI	2.40±0.47	1.47±0.48		
**Buduneli et al. (2009) [40]**	prospective cohort	CS (n=10)	sampling sites were PPD≥5 mm, CAL≥4 mm,	PPD	7.10±1.40	3.70±0.90	4 weeks	All clinical periodontal index values had decreased significantly 4 weeks after completion of the initial periodontal treatment in both patient groups (all P <0.01). In both groups, the volume of sampled GCF had decreased significantly after the initial periodontal treatment (P <0.01).
				CAL	7.50±1.10	5.60±1.20		
				PI	1.60±1.00	0.90±0.80		

			and the presence of BOP in single-rooted teeth	GI	1.90±0.90	0.50±0.40		
				GCF	1.15±0.47	0.75±0.53		
		NS (n=10)	sampling sites were PPD≥5 mm, CAL≥4 mm, and the presence of BOP in single-rooted teeth	PPD	5.90±1.20	4.30±1.60		
				CAL	7.80±1.70	6.90±1.60		
				PI	1.10±1.30	0.90±0.80		
				GI	1.90±0.60	0.90±0.40		
				GCF	0.84±0.40	0.50±0.40		
***Camargo et al. (2016) [41]**	prospective cohort	CS (n=24)	full mouth: at 6 sites per tooth excluding third molars	PPD*	NA	NA	6 months	At baseline, both groups had a similar periodontal status. Clinical conditions improved (decrease in PI and BOP indexes and in PPD and CAL measurements) in both groups, when baseline and 3 months after periodontal treatment were compared. Significant reductions were noted for PI, PPD, and CAL for NS and for PI, BOP, PPD, and CAL for CS, when the results for baseline and 6 months were compared. There was no statistical difference between the groups in periodontal parameters at 3 and 6 months, except for BOP in CS patients. Medians of PI, BOP, and PPD were lower at 3 months in NS. The same was observed for PI at 6 months, indicating that NS had a better response to periodontal treatment. Statistical differences between the groups were observed for PPD and CAL when comparing baseline and 3 months of treatment.
				CAL*	NA	NA		
				FMPS*	NA	NA		
				BOP*	NA	NA		
		NS (n=24)	full mouth: at 6 sites per tooth excluding third molars	PPD*	NA	NA		
				CAL*	NA	NA		
				FMPS*	NA	NA		
				BOP*	NA	NA		
***Chandra et al. (2011) (control) [42]**	prospective cohort	CS (n=3)	141 sites from six patients (67 sites from three non-smoker patients and 74 sites from three smoker patients) with PPD 5-7	PI*	1.30±NA	1.30±NA	90 days	Non-smokers showed a higher reduction in GI and smokers showed a higher reduction in PI. There was no significant gain in relative attachment level of both smokers and non-smokers.
				GI*	1.30±NA	1.30±NA		
		NS (n=3)		PI*	1.20±NA	1.20±NA		
				GI*	1.80±NA	1.80±NA		
**Chandra et al. (2012)**	RCT	CS (n=48)	interdental suprabony periodontal	PPD	6.02±1.49	5.16±1.52	6 months	PD and CAL at baseline and 3 and 6 months showed a significant reduction in mean scores in CS and NS (p<0.01) with the differences being more significant from the baseline to 3 and 6 months. PI and GI at baseline, 1 and
				CAL	6.20±1.22	5.41±1.07		

**(placebo) [43]**			pockets in molars with PPD ≥5 mm	GI	2.75±0.45	0.82±0.54		3 and 6 months showed a significant reduction in mean scores in CS and NS (p<0.01) with the differences being more significant from the baseline to 3 and 6 months.
				PI	3.59±0.68	1.84±0.57		
		NS (n=48)	interdental suprabony periodontal pockets in molars with PPD ≥5 mm	PPD	6.31±1.27	4.81±1.34		
				CAL	6.20±1.22	4.80±1.13		
				GI	2.80±0.43	0.86±0.66		
				PI	3.52±0.66	1.63±0.55		
**D'Aiuto et al. (2005) [44]**	prospective cohort	CS (n=40)	full mouth: at 6 sites per tooth	PPD	4.47±NA	3.43±NA	6 months	80% of variability in PD reductions was attributed to site level parameters, while 12% was at the tooth level and 8% at the patient level.The multilevel analysis associated PD reductions with patient factors, such as cigarette smoking status and carriage of the rare allele of a specific polymorphism for the interleukin-6 gene, tooth factors (tooth mobility and tooth type), and site factors (mesial and distal location).
		NS (n=57)	full mouth: at 6 sites per tooth	PPD	4.30±NA	3.03±NA		
**Darby et al. (2005) (CP) [45]**	prospective cohort	CS (n=10)	In each patient, 4 sites with PPD≥5 mm were selected, where possible one in each quadrant, with no furcation involvement.	PPD	5.90±1.50	4.90±1.40	8 weeks	CP non-smokers had significantly greater reductions in BOP (49% compared with 12.5%, p 0.02) and PD (1.7± 1.4 mm compared with 1.0 ±1.3 mm, p=0.007). Smokers had significantly deeper pockets (4.9 ±1.4) mm compared with 4.2 ± 1.6) mm, p=0.03) and greater relative attachment loss (13.8 ± 2.1) mm compared with 12.7 ± 2.3) mm, p=0.02. This group also had slightly more gingival inflammation, BOP, but less plaque than NS. However, these differences were not significant.
				GI	2.30±0.70	1.30±0.80		
				PI	1.50±1.10	0.80±0.80		
				BOP*	69.00±NA	56.00±NA		
		NS (n=48)	In each patient, 4 sites with PPD≥5 mm were selected, where possible one in each quadrant, with no furcation involvement.	PPD	5.90±1.20	4.20±1.60		
				GI	2.40±0.70	1.20±0.90		
				PI	1.50±0.90	1.10±0.90		
				BOP*	92.50±NA	44.00±NA		

**Do et al. (2023) [46]**	quasi-experimental study	CS (n=20)	full mouth	PPD	3.67±0.67	2.97±0.45	3 months	At baseline, there was no significant difference in any periodontal parameters between the two groups (p > 0.05). The PI, GI, and BOP also showed no significant difference between the two groups at T1 and T3 (p > 0.05). The PPD of the non-smoker group was significantly lower than those of the smoker group after NSPT (p < 0.05). The CAL of the non-smoker group was lower than that of the smoker group at T3; however, there was no significant difference in the CAL between the two groups (p > 0.05).
				CAL	4.10±0.77	3.67±0.08		
				GI*	NA	NA		
				PI*	NA	NA		
				BOP*	NA	NA		
		NS (n=20)	full mouth	PPD	3.38±0.41	2.54±0.39		
				CAL	3.90±1.10	3.18±0.97		
				GI*	NA	NA		
				PI*	NA	NA		
				BOP*	NA	NA		
**Eltas et al. (2012) (placebo) [47]**	RCT	CS (n=26)	test sites: PPD between 4 and 6 mm	PPD	5.40±0.70	4.20±0.50	6 months	At baseline, the mean PD, CAL, and PI were similar among the groups (p > 0.05); however, the GI and GCF volume were lower for the CS than for the NS (p < 0.05). Intragroup analysis revealed that the mean change in the PI, GI, PD, and GCF volume for all groups showed statistically significant differences at time points (p < 0.05). Only the changes in CAL for all groups proved to not be statistically significant at follow up (p > 0.05).
				CAL	6.60±1.20	6.50±1.10		
				GI	1.53±0.50	0.80±0.50		
				PI	1.70±0.60	0.70±0.50		
				GCF	1.03±0.31	0.57±0.25		
		NS (n=26)	test sites: PPD between 4 and 6 mm	PPD	5.30±0.80	3.60±0.50		
				CAL	6.10±0.90	5.80±1.00		
				GI	1.91±0.60	0.86±0.60		
				PI	1.80±0.50	0.60±0.50		
				GCF	1.29±0.45	0.62±0.29		
**Erdemir et al. (2004) [49]**	prospective cohort	CS (n=22)	4 sample site/pt >5mm	PPD	5.28±0.24	5.33±0.28	6 months	In smokers, only CAL was significantly higher at the 3rd month compared with non-smokers (p<0.05). GI and BOP were higher in non-smokers than smokers in both periods (p<0.05). PI showed increases from the initial to the 6th month in smokers (p<0.05).
				CAL	5.31±0.78	5.17±0.72		
			full mouth	GI	1.48±0.40	1.40±0.28		
				PI	1.56±0.37	1.90±0.30		
				BOP	48.00±40.00	40.00±28.00		
		NS (n=19)	4 sample site/pt >5mm	PPD	5.21±0.29	5.17±0.19		
				CAL	4.54±0.49	4.52±0.58		
			full mouth	GI	1.59±0.34	1.89±0.17		

				PI	1.33±0.31	1.84±0.24		
				BOP	59.00±34.00	88.00±15.00		
***Erdemir et al. (2007) [50]**	prospective cohort	CS (n=45)	full mouth: at six sites per tooth of all teeth	PPD*	4.05±NA	3.08±NA	6 months	Mean PI was significantly greater in smokers compared with non-smokers throughout the observation period (p<0.001). During the first month, GI levels significantly decreased in both groups. From months 1 through 6, a significant return towards an increased GI level was observed in smokers (p<0.001). PD and CAL levels significantly decreased during the first month in both groups. Thereafter, increasing levels of PD and CAL were seen in both groups, although significantly more pronounced in smokers. Throughout the observation period, the mean CAL was significantly greater in smokers relative to nonsmokers (p<0.001).
				CAL*	3.84±NA	3.3±NA		
				GI*	1.82±NA	1.42±NA		
				PI*	2.06±NA	1.73±NA		
		NS (n=43)	full mouth: at six sites per tooth of all teeth	PPD*	3.85±NA	2.88±NA		
				CAL*	3.41±NA	2.85±NA		
				GI*	1.92±NA	1.43±NA		
				PI*	1.89±NA	1.15±NA		
**Feres et al. (2015) [50]**	prospective cohort	CS (n=15)	full mouth: at 6 sites per tooth excluding third molars	PPD	3.90±0.60	3.30±0.60	6 months	An improvement in clinical condition was observed for smokers and non-smokers; however, non-smokers showed a greater reduction in mean clinical attachment level in intermediate sites in comparison with smokers (p < 0.05). Smokers were more susceptible to the re-establishment of a pathogenic subgingival biofilm than nonsmokers.
				CAL	4.70±1.20	4.20±0.90		
				FMPS	66.60±22.90	35.70±19.90		
				BOP	67.00±18.20	53.90±24.60		
		NS (n=15)	full mouth: at 6 sites per tooth excluding third molars	PPD	3.80±0.70	3.10±0.40		
				CAL	4.40±1.00	3.90±0.90		
				FMPS	82.20±12.00	44.50±14.40		
				BOP	62.00±19.30	23.60±14.10		
**Gaind et al. (2024) [51]**	prospective cohort	CS (n=20)	full mouth	PI	2.25±0.36	1.72±0.28	3 months	Both groups' clinical periodontal parameters improved significantly following non-invasive periodontal therapy. Compared to the CS group, NS group had better treatment outcomes.
				GI	1.94±0.41	1.58±0.27		
		NS (n=20)	full mouth	PI	1.87±0.50	1.54±0.31		
				GI	2.23±0.29	1.88±0.37		
**Ghallab et al. (2010) [52]**	prospective cohort	CS (n=11)	full mouth: at 6 sites per tooth excluding third molars	PI	0.64±0.50	0.54±0.52	1 months	The two studied groups showed similar mean levels of PI at baseline. Although both groups showed a reduction in plaque scores at 1 month, it was not significant compared to baseline values. A highly significant difference was noticed in the baseline GI values (P value = 0.001) when CS were compared to NS. At 1 month both CS and NS showed a reduction in GI but no significant differences were observed between the two studied groups.
				GI	0.55±0.52	0.27±0.47		
				PI	0.64±0.50	0.45±0.52		

		NS (n=11)	full mouth: at 6 sites per tooth excluding third molars	GI	1.64±0.50	0.36±0.50		However, this reduction was highly significant compared to baseline values in the NS group (P value = 0.002)
**Grossi et al. (1997) [53]**	prospective cohort	CS (n=60)	PPD ≥5 mm	PPD	6.00±NA	NA	3 months	Results indicate that CS have less healing after treatment compared to FS and NS, suggesting that smoking impairs periodontal healing. Reduction in whole mouth mean PPD was significantly less (P < .04) in CS compared to FS and NS. Although not significant, the gain in mean whole-mouth CAL was also less in CS compared to FS and NS. Each group showed a significant reduction (P < .003) in PI at 3 months. FS showed significantly greater reductions (P < .002) compared to CS and NS. CS showed significantly less gingival bleeding (P < .0001) at baseline compared to FS and NS. All three groups showed a reduction in GI at 3 months, with the greatest reduction occurring in FS. As the healing a of former smokers is comparable to that of NS, smoking cessation may restore the normal periodontal healing response.
				CAL	6.40±NA	NA		
			full mouth: at 6 sites per tooth excluding third molars	PPD	3.01±NA	NA		
				CAL	3.40±NA	NA		
				GI	0.23±NA	NA		
				PI	1.30±NA	NA		
		FS (n=55)	PPD ≥5 mm	PPD	6.10±NA	NA		
				CAL	6.60±NA	NA		
			full mouth: at 6 sites per tooth excluding third molars	PPD	3.00±NA	NA		
				CAL	3.40±NA	NA		
				GI	0.38±NA	NA		
				PI	1.30±NA	NA		
		NS (n=28)	PPD ≥5 mm	PPD	6.40±NA	NA		
				CAL	6.70±NA	NA		
			full mouth: at 6 sites per tooth excluding third molars	PPD	3.00±NA	NA		
				CAL	3.20±NA	NA		
				GI	0.37±NA	NA		
				PI	1.00±NA	NA		
**Guentsch et al. (2008) [54]**	prospective cohort	CS (n=15)	full mouth: at 6 sites per tooth	PPD	3.79±0.72	2.59±0.75	6 months	There were no statistically significant differences in mean PPD between smokers and non-smokers. Mean probing depths were significantly reduced after periodontal treatment in the periodontitis patients (p<0.05). After periodontal treatment, a significant reduction in BOP was recorded in both groups (p<0.05).
				BOP	93.87±6.67	17.95±14.98		
		NS (n=15)	full mouth: at 6 sites per tooth	PPD	3.85±0.66	2.30±0.75		
				BOP	90.2±21.06	21.62±9.86		
			full mouth	PPD	4.95±0.65	3.10±0.75	1 months	

**Guru et al. (2018) [55]**	prospective cohort	CS (n=25)		CAL	4.87±0.24	3.55±0.57		Although CS benefitted from the periodontal treatment, the clinical improvements were less than that those observed for NS. Both CS and NS showed a statistically-significant reduction in mean PI, PPD, CAL between baseline and 30 days' post-treatment (P < .001). Results showed a statistically-significant difference in mean PPD, and CAL between the two groups 30 days' post-treatment, however there was no statistical difference in PI scores.
				PI	2.19±0.43	0.90±0.10		
		NS (n=25)	full mouth	PPD	4.31±0.82	2.36±0.42		
				CAL	4.14±0.35	2.91±0.79		
				PI	2.09±0.43	0.83±0.14		
**Hadžić et al. (2023) [56]**	prospective cohort	CS (n=7)	full mouth: at 6 sites per tooth	PPD	6.20±0.21	5.02±0.28	3 months	CS had significant reduction in PPD and FMPS 3 months after periodontal treatment, NS had significant reduction in PPD and BOP (<.0001).
				CAL	9.93±1.04	9.36±0.85		
				FMPS	64.02±10.32	48.20±7.79		
				BOP	47.87±20.43	34.28±14.01		
		NS (n=7)	full mouth: at 6 sites per tooth	PPD	6.13±0.09	5.03±0.09		
				CAL	9.75±1.03	9.09±1.08		
				FMPS	65.47±10.28	54.18±8.96		
				BOP	62.33±9.73	47.71±9.75		
***Hendek et al. (2015) [57]**	case-control study with intervention arm	CS (n=24)	full mouth: at 6 sites per tooth; 8 GCF sample teeth/patient with PPD 4-7 mm and ≥30% bone loss	GI*	NA	NA	3 months	After initial periodontal therapy, periodontal clinical parameters of whole mouth and sampling teeth significantly decreased from baseline to the 1st and the 3rd months in both CS and NS (p<0.001) and no differences were observed between the 1st and the 3rd months. Only whole mouth (WM) GI values more significantly decreased in CS group than the other group. When comparisons were evaluated with respect to time, WMPI values at the 1st month and WMPD values at the 3rd month were significantly higher in CS group compared to NS group. Sampling teeth (ST) PD and STGI values more significantly decreased in NS group than CS group. When comparisons were evaluated with respect to time, STGI values at the 1 month and STPD and STGI values at the 3 month were significantly higherd in CS group compared to NS group. After periodontal therapy, GCF volume significantly decreased from baseline to the 1st and the 3rd months in both groups (p<0.001). This reduction was significantly greater in CS compared to NS.
				PI*	NA	NA		
				PPD*	NA	NA		
				CAL*	NA	NA		
				GCF*	NA	NA		
		NS (n=23)	full mouth: at 6 sites per tooth; 8 GCF sample teeth/patient with PPD 4-7 mm and ≥30% bone loss	GI*	NA	NA		
				PI*	NA	NA		
				PPD*	NA	NA		
				CAL*	NA	NA		
				GCF*	NA	NA		
**Hendek et al. (2020) [59]**	prospective cohort	CS (n=20)	sample teeth: single-rooted teeth with	PPD	5.48±0.48	2.94±0.48	6–8 weeks	PPD, CAL, and PI values in full mouth and CAL and PI values in sample teeth were significantly higher in CS, whereas GI values in both full mouth and sample teeth were significantly higher in NS (p < .01). All clinical periodontal parameters (both full mouth
				CAL	6.04±0.70	3.38±0.85		
				GI	1.59±0.30	0.92±0.25		

			≥4 and <7 mm PPD and ≥30% bone loss	PI	1.36±0.31	0.54±0.31		and sample teeth) showed a significant decrease after initial periodontal therapy in both CP groups (p < .01)
				GCF	0.26±0.11	0.16±0.07		
			full mouth: at 6 sites per tooth;	PPD	3.79±0.45	2.67±0.44		
				CAL	4.29±0.57	3.02±0.60		
				GI	1.56±0.23	1.12±0.33		
				PI	1.35±0.30	0.69±0.34		
		NS (n=20)	sample teeth: single-rooted teeth with ≥4 and <7 mm PPD and ≥30% bone loss	PPD	5.36±0.34	2.75±0.46		
				CAL	5.66±0.50	3.68±0.99		
				GI	1.91±0.14	0.62±0.14		
				PI	1.13±0.26	0.44±0.28		
				GCF	0.41±0.09	0.16±0.06		
			full mouth: at 6 sites per tooth;	PPD	3.45±0.46	2.41±0.41		
				CAL	3.87±0.50	2.89±0.60		
				GI	1.78±0.23	0.87±0.17		
				PI	1.11±0.25	0.53±0.27		
**Ikhar et al. (2024) [58]**	prospective cohort	CS (n=20)	full mouth: at 6 sites per tooth	PPD	8.06±0.19	5.60±0.19	3 months	Greater values of PPD, CAL, PI while lower GI scores were seen in CS as compared to NS, which reduced significantly from baseline to 3months in both the groups after NSPT.
				CAL	8.94±0.19	6.85±0.19		
				GI	1.72±0.19	0.74±0.18		
				PI	2.58±0.19	0.74±0.18		
		NS (n=20)	full mouth: at 6 sites per tooth	PPD	6.86±0.19	4.10±0.19		
				CAL	7.07±0.19	4.88±0.23		
				GI	2.15±0.19	1.10±0.19		
				PI	1.83±0.19	0.54±0.18		
**Lütfioğlu et al. (2021) [60]**	prospective cohort	CS (n=15)	full mouth: at 6 sites per tooth, each highest value per tooth was taken	PPD	7.34±1.47	7.09±1.36	4 weeks	Clinical parameters in both smokers and non-smokers improved following periodontal treatment (P < .001) and their clinical data were similar for all the examination times (P > .05).
				CAL	8.67±1.39	8.26±1.32		
				PI	2.06±0.31	1.11±0.47		
				GI	1.15±0.28	0.73±0.20		

			for evaluations	BOP	68.73±4.62	8.39±2.98		
		NS (n=15)	full mouth: at 6 sites per tooth, each highest value per tooth was taken for evaluations	PPD	7.32±1.03	7.21±1.01		
				CAL	8.09±1.08	7.40±1.03		
				PI	1.73±0.40	0.90±0.21		
				GI	1.56±0.29	0.65±0.13		
				BOP	81.84±3.76	13.49±3.24		
**Maged et al. (2022) [29]**	non-randomized clinical trial	CS (n=20)	full mouth: at 6 sites per tooth	PPD	6.40±1.17	3.00±0.67	3 months	In all clinical parameters (GI, PI, PPD, CAL), there was no significant difference between group NS and CS either at baseline or 3 month after treatment. In both groups, there was a statistically significant improvement in all clinical parameters 3 months after nonsurgical treatment.
				CAL	7.20±1.87	5.40±1.81		
				PI	2.70±0.48	0.30±0.48		
				GI	2.30±0.82	0.70±0.82		
		NS (n=20)	full mouth: at 6 sites per tooth	PPD	5.90±0.88	2.90±0.57		
				CAL	6.40±1.78	4.80±1.32		
				PI	2.60±0.52	0.30±0.48		
				GI	2.30±0.67	0.30±0.48		
**Mantyla et al. (2006) [61]**	longitudinal study	CS (n=11)	7–10 sites/patient with PPD ≥4 mm	PPD	4.90±1.70	3.60±1.60	12 months	The treatment outcome measured as PPD was significantly poorer in smokers' sites than in nonsmokers' sites, and the difference remained during the maintenance phase. In CAL values the difference was not as clear.
				CAL	4.40±3.50	3.40±2.40		
				BOP*	63.00±NA	13.00±NA		
		NS (n=5)	7–10 sites/patient with PPD ≥4 mm	PPD	5.00±2.10	2.80±1.30		
				CAL	4.40±3.50	3.40±2.90		
				BOP*	86.00±NA	28.00±NA		
**Mashalkar et al. (2024) [62]**	prospective cohort	CS (n=50)	full mouth	PPD	6.50±1.20	5.30±0.90	6 months	Following NSPT, both groups showed significant improvements in periodontal health, with non-smokers experiencing greater gains: probing depth reduced by 21.7% in non-smokers and 18.5% in smokers, clinical attachment level improved by 21.1% and 16.7%, bleeding on probing decreased by 30.8% and 28.6%, and plaque index reduced by 30.4% and 24.0%, respectively. Improvements were more pronounced in non-smokers.
				CAL	4.20±1.00	3.50±0.70		
				BOP	70.00±15.00	50.00±8.00		
				PI	2.50±0.60	1.90±0.30		
		NS (n=50)	full mouth	PPD	6.00±1.10	4.70±0.80		
				CAL	3.80±0.90	3.00±0.60		
				BOP	65.00±12.00	45.00±7.00		

				PI	2.30±0.50	1.60±0.20		
**Maske et al. (2023) (control) [63]**	RCT	CS (n=20)	split mouth study, total 80 sites with PPD≥ 5 mm	PI	2.17±0.40	0.82±0.23	6 months	Control sites showed significant improvement in PI and GI values after periodontal therapy in all groups. Control sites also showed significant improvement in PPD at 6 months, but CAL showed non-significant changes at 6 months after periodontal therapy in group CS and NS.
				GI	1.91±0.30	0.88±0.37		
				PPD	5.30±0.73	5.10±0.78		
				CAL	5.30±0.73	5.10±0.85		
		NS (n=20)	split mouth study, total 80 sites with PPD≥ 5 mm	PI	2.25±0.40	0.98±0.28		
				GI	2.27±0.44	0.58±0.24		
				PPD	5.25±0.91	4.20±0.95		
				CAL	5.30±0.92	4.70±1.08		
**Meenawat et al. (2015) [64]**	prospective cohort	CS (n=12)	full mouth	PPD	5.97±0.55	5.52±0.53	1 months	The improvement in the periodontal parameters was statistically significant in NS, while CS showed lesser improvement. The mean PI reduced significantly in CS and NS after 1-month (P= 0.0001). Regarding GI, the percentage improvement in NS was 75.1% (P= 0.0002), and in smokers it was 52.6% (P= 0.0001). Regarding PPD, following periodontal therapy there was a 12.2% improvement in NS, (P= 0.0001) and for CS it was 7.5% (P= 0.0001). Regarding CAL, improvements of 27.6% and 22.9% were seen in NS and CS after treatment (P= 0.0002), though intergroup comparison posttreatment showed no statistical significance (P= 0.0223)
				CAL	4.05±0.66	3.12±0.58		
				PI	2.51±0.26	1.61±1.16		
				GI	1.34±0.36	0.64±0.21		
		NS (n=12)	full mouth	PPD	5.79±0.58	5.09±0.60		
				CAL	3.46±0.68	2.50±0.61		
				PI	2.46±0.21	1.01±0.33		
				GI	2.46±0.23	0.61±0.18		
**Mittal et al. (2018) [65]**	prospective cohort	CS (n=50)	full mouth	PPD	4.84±0.59	4.11±0.57	3 months	Tobacco consumption in both forms affects the severity of periodontal disease. CS had significantly more PPD at baseline examination, while tobacco chewers had more gingival recession. Gingival inflammation, response to non-surgical treatment and oral hygiene maintenance were more suppressed in smokers as compared to tobacco chewers.
				PI	1.71±0.43	1.22±0.28		
				GI	1.15±0.26	0.98±0.22		
				GCF	0.04±0.006	0.041±0.002		
		ST (n=50)	full mouth	PPD	4.25±0.53	3.42±0.68		
				PI	1.30±0.56	0.92±0.56		
				GI	1.52±0.58	1.20±0.47		
				GCF	0.047±0.07	0.045±0.007		
		NS (n=50)	full mouth	PPD	4.38±0.37	3.00±0.77		
				PI	1.37±0.31	0.86±0.27		

				GI	1.78±0.43	0.74±0.19		
				GCF	0.052±0.008	0.033±0.004		
**Mouzakiti et al. (2012) [66]**	prospective cohort	CS (n=15)	sampling site	PPD	5.90±1.2	4.50±1.2	6 weeks	The CS and NS groups did not statistically significantly differ in pretreatment clinical measurements. Periodontal treatment led to statistically significant reduction in all clinical measurements in both experimental groups. Changes in all clinical measurements achieved with periodontal treatment were not statistically significantly different between the two groups.
				CAL	6.50±1.5	5.40±1.8		
			full mouth	PPD	3.60±0.70	2.90±0.60		
				CAL	4.00±NA	3.20±NA		
				FMPS	71.70±21.00	14.70±7.10		
				BOP	68.90±19.00	17.10±9.40		
		NS (n=15)	sampling site	PPD	7.10±2.10	5.60±1.70		
				CAL	7.40±2.40	6.70±2.40		
			full mouth	PPD	3.70±0.80	3.00±0.70		
				CAL	3.50±NA	3.00±NA		
				FMPS	77.30±16.10	16.30±7.20		
				BOP	69.30±15.90	23.90±13.80		
**Öngöz-Dede et al. (2023) [67]**	prospective cohort	CS (n=15)	full mouth: six sites around each tooth	PPD	3.09±0.43	2.45±0.43	6 weeks	For both smokers and non-smokers, a significant reduction was observed after the NSPT in PI, GI, and BOP values ($p < .001$). GI and BOP values were found to be significantly lower in smokers than in non-smokers ($p < .001$). There was no statistically significant difference between CS and NS periodontitis groups in the PD and CAL ($p > .05$). There was a statistically significant decrease in PD in the periodontitis groups with smokers and non-smokers ($p < .001$).
				CAL	4.57±0.99	3.90±0.79		
				PI	1.99±0.47	0.64±0.27		
				GI	0.94±1.23	0.20±0.2		
				BOP	38.02±10.78	13.04±2.92		
		NS (n=15)	full mouth: six sites around each tooth	PPD	3.22±0.54	2.23±0.48		
				CAL	5.04±0.92	4.38±0.93		
				PI	1.72±0.47	0.58±0.3		
				GI	1.36±1.83	0.56±0.32		
				BOP	64.68±19.99	18.15±11.52		
**Özdemir et al. (2021) (control) [68]**	RCT	CS (n=15)	full mouth: on six surfaces of each tooth	PPD	4.96±0.42	2.54±0.42	6 weeks	There were no statistical differences between all groups at baseline ($P > 0.05$) regarding clinical data. The PI, GI, PPD and CAL levels were significantly improved after nonsurgical periodontal treatment, comparison of baseline and 6w after treatment data ($P < 0.05$) in all groups. BOP parameters were increased in all groups after the nonsurgical periodontal
				CAL	8.27±1.14	7.00±1.35		
				PI	1.90±0.5	0.10±0.3		
				GI	2.00±0.4	0.40±0.2		

			except third molars	BOP*	100±NA	6.80±NA		treatment. There were no statistical differences between CS and NS groups at the comparison in each other.
		NS (n=15)	full mouth: on six surfaces of each tooth except third molars	PPD	4.75±0.48	2.82±0.65		
				CAL	8.56±1.69	7.53±0.94		
				PI	1.80±0.5	0.20±0.1		
				GI	2.00±0.4	0.30±0.2		
				BOP*	100±NA	8.50±NA		
***Padhye et al. (2019) [69]**	longitudinal study	CS (n=15)	diseased site	PPD	6.07±NA	5.27±NA	12 weeks	Greater periodontal destruction is seen in current smokers than former and nonsmokers with CP. Increased PPD (P = 0.01) and decreased GI (P = 0.021) was noted in CS as compared to FS and NS at baseline. A sharp decrease in the GI, BOP, PPD were seen in Groups FS and NS as compared to Group CS. Periodontal intervention caused a greater resolution of inflammation in Groups FS and NS compared to CS.
			full mouth	GI*	0.37±NA	0.14±NA		
				BOP*	37.52±NA	20.71±NA		
				PPD*	4.44±NA	2.84±NA		
				CAL*	4.62±NA	3.33±NA4.35±NA		
		FS (n=15)	diseased site	PPD	5.33±NA	4.35±NA		
			full mouth	GI*	0.44±NA	0.11±NA		
				BOP*	40.18±NA	19.39±NA		
				PPD*	3.37±NA	2.51±NA		
				CAL*	4.58±NA	3.34±NA		
		NS (n=15)	diseased site	PPD	5.65±NA	4.65±NA		
			full mouth	GI*	0.45±NA	0.12±NA		
				BOP*	44.44±NA	18.44±NA		
				PPD*	4.65±NA	2.92±NA		
				CAL*	5.33±NA	3.59±NA		
**Palmer et al. (1999) (SRP) [70]**	prospective cohort	CS (n=9)	sites with baseline probing depths equal to or greater than 5 mm	PPD	5.73±0.51	4.61±0.7	6 months	Reductions in PPD at 6 months were significantly less (p<0.001) in CS than in NS. Attachment level gains were approximately 0.55 mm and there was no statistically significant difference between smokers and non-smokers. In BOP and plaque score, there was no statistically significant difference between CS and NS.
				CAL	NA	NA		
				FMPS	18.20±9.1	11.2±7.8		
				BOP	21.40±9.4	12.10±6.9		
		NS (n=18)	sites with baseline probing	PPD	5.92±0.49	3.96±0.37		
				CAL	NA	NA		

			depths equal to or greater than 5 mm	FMPS	19.30±14.8	13.10±10.9		
				BOP	24.20±18.7	18.20±16.3		
***Pamuk et al. (2017) (SRP+Sham) [71]**	split-mouth RCT	CS (n=15)	For each subject, five teeth with the highest probing depth and clinical attachment level values	PI*	NA	NA	30 days	All clinical parameters showed significant reductions between baseline and day 30 following NSPT in both groups (P<.001). No significant differences were observed between smokers or non-smokers (P>.05).
				GI*	NA	NA		
				BOP*	NA	NA		
				PPD*	NA	NA		
				CAL*	NA	NA		
		NS (n=15)	For each subject, five teeth with the highest probing depth and clinical attachment level values	PI*	NA	NA		
				GI*	NA	NA		
				BOP*	NA	NA		
				PPD*	NA	NA		
				CAL*	NA	NA		
**Preber et al. (1986) [72]**	prospective cohort	CS (n=10)	full mouth: on four sites of all teeth	PI	0.58±0.31	0.12±0.12	1 months	The treatment caused a reduction in PI and gingival bleeding both in CS and in NS. The plaque reduction was significantly greater in CS. Nevertheless, the reduction in gingival bleeding was significantly less pronounced than that attained in NS. The findings suggest that the gingival bleeding response to treatment is reduced in smokers. It would seem that in response to a given amount of plaque reduction the changes in gingival bleeding will be less apparent under the influence of smoking.
				BOP	27.10±12.2	12.70±6.4		
			pathological pockets (≥4 mm)	PI	0.74±0.4	0.19±0.17		
				BOP	41.50±11.6	16.10±9.1		
		NS (n=10)	full mouth: on four sites of all teeth	PI	0.31±0.24	0.07±0.07		
				BOP	39.90±9.8	13.10±5.9		
			pathological pockets (≥4 mm)	PI	0.46±0.39	0.1±0.09		
				BOP	75.90±14.5	27.60±8.7		
**Preber et al. (1985) [73]**	prospective cohort	CS (n=40)	full mouth	PPD	4.50±0.24	3.37±0.36	1 months	Plaque index was reduced to a minitnum in both smokers and non-smokers following treatment. An average reduction in probing pocket depth of 1,1 mm in smokers and 1,2 mm in non-smokers was observed. The reduction
				PI	1.46±0.48	0.17±0.15		
			full mouth	PPD	4.45±0.20	3.22±0.31		

		NS (n=35)		PI	1.23±0.39	0.13±0.1		attaitied was less in smokers than in non-stnokers for all regions of the dentition investigated.
**Preber et al. (1995) [74]**	prospective cohort	CS (n=17)	full mouth	GI	1.00±0.3	NA	2 months	The treatment resulted in significant reductions towards very low plaque and gingival indices in smokers and nonsmokers alike (p<0.05). Although probing depth was reduced in both smokers and non-smokers, probing pocket depth reduction was significantly smaller in smokers than non-smokers (p<0.05).
				PI	0.90±0.3	NA		
				PPD	4.30±0.6	3.40±0.4		
			1 site with a PPD of more than 5 mm	PPD	7.10±0.8	NA		
		NS (n=15)	full mouth	GI	1.40±0.3	NA		
				PI	0.80±0.3	NA		
				PPD	3.80±0.7	2.70±0.4		
			1 site with a PPD of more than 5 mm	PPD	7.40±1.1	NA		
***Preshaw et al. (1999) [76]**	prospective cohort	CS (n=15)	Eight periodontally involved test sites per subject	PI*	NA	NA	7 months	Pretreatment: no clinically significant changes in mean PI, PPD, BOP or CAL were detected (P >0.05). Post-treatment: statistically and clinically significant reductions were observed in mean PI, BOP, and PD (P <0.05). A statistically significant reduction in CAL was noted (P <0.05). Smokers, non-smokers, and ex-smokers did not differ significantly in PI, BOP, CAL, radiographic, or biochemical parameters at any time. Mean PD was significantly greater in current smokers than in non- and ex-smokers (P <0.005). PD reduced comparably in all 3 smoking subgroups following treatment (P <0.01).
				PPD*	NA	NA		
				BOP*	NA	NA		
		FS (n=14)	Eight periodontally involved test sites per subject	PI*	NA	NA		
				PPD*	NA	NA		
				BOP*	NA	NA		
		NS (n=12)	Eight periodontally involved test sites per subject	PI*	NA	NA		
				PPD*	NA	NA		
				BOP*	NA	NA		
**Preshaw et al. (2005) [75]**	prospective cohort	CS (n=10)	diseased sites (>3 mm)	PPD	4.84±0.33	3.69±0.38	12 months	Quitting smoking has an additional beneficial effect in reducing PPD following non-surgical treatment over a 12-month period. There were no differences between the groups following treatment with respect to mean clinical or radiographic parameters. Analysis of PPD reductions between baseline and month 12 demonstrated a significant difference in favour of quitters (p<0.05). Furthermore, quitters were significantly more likely to
				CAL	10.70±0.8	10.5±1.4		
			full mouth	PPD	3.44±0.62	2.83±0.23		
				BOP	68.80±23.00	54.60±7.7		

				FMPS	86.8±10.7	69.70±30.8		demonstrate PPD reductions ≥2 and ≥3mm than non-quitters and oscillators (p<0.05).
		NS (n=10)	diseased sites (>3 mm)	PPD	5.31±0.43	3.71±0.54		
				CAL	12.20±1.40	12.30±1.3		
			full mouth	PDP	3.98±0.68	3.02±0.52		
				BOP	65.10±24.60	45.80±20.7		
				FMPS	87.80±10.00	73.40±14.0		
**Pucher et al. (1997) [77]**	prospective cohort	CS (n=33)	target sites (PPD 5-8 mm, bleeding upon probing)	PPD	5.70±0.66	5.05±0.75	9 months	Both NS and CS had a statistically significant decrease (P < 0.05) in PPD at 3 months which was maintained throughout the study. Both CS and NS displayed a significant gain (P < 0.05) in CAL after initial therapy when compared to baseline readings. PI scores remained consistent for smokers and non-smokers for the duration of the study. The GI at baseline was significantly (P < 0.05) lower in CS than NS. By 9 months only the GI of NS decreased significantly compared to baseline. At 9 months both CS and NS had a significant decrease in bleeding on probing compared to baseline. At 9 months there were no significant differences between smokers and non-smokers comparing all indices. The data have shown that smokers and non-smokers responded similarly.
				CAL	5.79±0.86	5.20±0.82		
				PI	1.32±0.57	1.16±0.66		
				GI	1.32±0.45	1.29±0.53		
				BOP	100.0±9.0	67.00±39.0		
		NS (n=54)	target sites (PPD 5-8 mm, bleeding upon probing)	PPD	5.46±0.46	4.86±0.7		
				CAL	5.50±0.75	5.03±0.87		
				PI	1.48±0.57	1.45±0.67		
				GI	1.45±0.40	1.26±0.37		
				BOP	100.0±7.0	78.0±30.0		
**Renvert et al. (1998) [78]**	prospective cohort	CS (n=13)	experimental sites	PI	0.70±0.32	0.28±0.25	6 months	Baseline values regarding clinical data did not differ significantly between the groups. 6 months following therapy the full-mouth bleeding score among smokers was 36.5% as compared to 22.7% for non-smokers (p<0.05). Probing depth was reduced by 1.9 mm for smokers and by 2.5 mm for non-smokers. This difference was statistically significant (p<0.05).
				BOP	97.00±6.0	67.00±32.0		
				PPD	7.10±0.80	5.20±0.9		
			full mouth	FMPS	61.50±17.40	22.80±11.6		
				BOP	63.00±21.30	36.50±19.9		
		NS (n=15)	experimental sites	PI	0.72±0.29	0.20±0.25		
				BOP	98.00±6.00	70.00±25.0		
				PPD	7.60±1.10	5.10±0.80		
			full mouth	FMPS	54.70±18.60	24.00±16.0		
				BOP	53.00±24.30	22.70±12.3		
**Rosa et al. (2011) [79]**	prospective cohort	CS (n=35)	sites presenting	CAL	5.76±1.21	4.91±1.39	12 months	Smoking cessation promoted clinical attachment gain in chronic periodontitis subjects after 1 year of follow-up. After 1 year, only quitters presented significant clinical attachment gain (p<0.04). However, there were
				PPD	4.72±0.50	3.63±0.76		

			baseline PPD ≥4mm					no differences between the groups regarding clinical attachment level, probing depth, bleeding on probing and plaque index after 1 year (p<0.05).
			full mouth	FMPS	81.62±19.66	72.09±20.88		
				CAL	4.18±1.39	4.05±1.21		
				PPD	3.07±0.73	2.77±0.65		
				BOP	19.75±13.83	20.04±13.15		
		Q (n=17)	sites presenting baseline PPD ≥4mm	CAL	5.71±1.28	4.39±1.18		
				PPD	4.66±0.49	3.42±0.73		
			full mouth	FMPS	85.08±20.86	58.62±28.97		
				CAL	3.73±0.68	3.52±0.55		
				PPD	2.76±0.50	2.47±0.34		
				BOP	25.61±17.62	20.41±11.63		
**Ryder et al. (1999) (SRP) [80]**	RCT	CS (n=61)	sites with PPD ≥5mm	CAL	5.68±NA	NA	9 months	In the scaling and root planing treated group in general, there were significant differences in clinical attachment gain and pocket depth reduction,<br>with non-smokers responding better than former smokers and current smokers at 6 and 9 months.
				PPD	5.72±NA	NA		
		FS (n=72)	sites with PPD ≥5mm	CAL	6.00±NA	NA		
				PPD	5.94±NA	NA		
		NS (n=48)	sites with PPD ≥5mm	CAL	5.31±NA	NA		
				PPD	5.99±NA	NA		
**Salhi et al. (2020) [81]**	prospective cohort	CS (n=34)	full mouth: at 6 sites of each tooth	PPD	3.78±1.05	2.30±0.53	3 months	OHI led to a highly significant decrease of mean BOP (%) values (p < 0.0001) in FS and NS but in not in CS (p = 0.94). OHI also significantly reduced the mean PI in all three groups (p < 0.0001) with reductions of 74.8% in FS, 64.7% in NS, and 45.4% in CS. Mean and max PD, mean and max CAL, % PD > 6 mm remained unchanged. Full mouth subgingival debridement induced an additional significant reduction (p < 0.0001) in all periodontal parameters in all three smoking groups except for mean BOP (%), and mean PI where values remained unchanged. Of important note, the mean PD dropped by 1.44 mm in CS, 1.30 mm in FS, and by 1.07 mm in NS. Similarly, max PD decreased by 1.68, 3.08, and 2.96 mm, respectively. The percentage of PD >6 mm went down from 18.2 to 2.92 in CS, 12.7 to 0.25 in FS, and 11.7 to 0.42 in NS. FS had comparable outcomes to NS, suggesting that smoking cessation should be encouraged.
				CAL	4.34±1.4	2.72±0.67		
				BOP	12.00±23	0.42±1.58		
				FMPS	86.40±17.7	30.40±21.1		
		FS (n=25)	full mouth: at 6 sites of each tooth	PPD	3.27±0.7	1.84±0.28		
				CAL	3.40±0.81	1.88±0.3		
				BOP	52.60±26.1	1.21±4.69		
				FMPS	80.9±17.8	11.30±15.3		
		NS (n=32)	full mouth: at 6 sites of each tooth	PPD	3.05±0.63	1..86±0.33		
				CAL	3.10±0.68	1.92±0.38		

				BOP	54.50±27.3	1.55±3.22		
				FMPS	77.80±22.9	9.38±10.1		
**Savran et al. (2024) (control) [82]**	retrospective	CS (n=20)	full mouth: at 6 sites of each tooth	PPD	3.15±0.49	2.26±0.39	3 months	All clinical parameters were reduced at 1 and 3 months after periodontal therapy compared to baseline in all groups ($p < 0.001$). Whole mouth PD was lower at 3 months compared to 1 month for all groups ($p < 0.05$). There was no significant difference between all groups at baseline in terms of whole mouth clinical parameters ($p \geq 0.05$). At 1 and 3 months after therapy, whole mouth PD were lower in NS than CS ($p < 0.05$). Whole mouth CAL, GI and BOP (%) were significantly lower in NS, compared to CS ($p < 0.05$) at 1 and 3 months postoperatively. There was no significant difference between study groups in terms of PI ($p > 0.05$). Multiple regression analysis showed that smoking was negatively associated with improvements in PD and CAL changes at baseline and 3 months post-treatment for moderately deep pockets and deep pockets.
				CAL	3.62±1.1	2.54±1.08		
				GI	1.63±0.53	0.65±0.39		
				PI	1.57±0.44	0.76±0.31		
				BOP	51.98±25.57	16.22±9.89		
		NS (n=20)	full mouth: at 6 sites of each tooth	PPD	3.51±0.61	2.73±0.49		
				CAL	4.33±1.33	3.41±1.04		
				GI	1.66±0.14	1.08±0.46		
				PI	1.79±0.61	0.97±0.32		
				BOP	65.81±22.5	28.68±14.64520		
**Shah et al. (2023) [17]**	retrospective	CS (n=20)	full mouth	PPD	3.48±1.3	2.86±0.89	28.7±14.7 weeks	Compared with NS, EC users had a less favorable treatment response after professional-mechanical-plaque removal. This included statistically significant increased "need for surgery", as well as increased number of sextants with PD ≥5 mm, number of sites with PD >5mm and mean PD. There were no statistically significant differences between EC users and CS. FS responded statistically significantly better than EC users for the primary outcome (need for surgery) as well as for the number of sextants and sites with PD≥5mm and mean PD. Overall, EC users had a statistically significantly less favorable response than NS; their response was not statistically significantly different to those of CS.
				CAL	4.17±1.40	3.74±1.30		
				FMPS	47.80±21.60	23.50±12.20		
				BOP	43.80±23.60	22.60±18.40		
		FS (n=60)	full mouth	PPD	3.44±0.95	2.72±0.59		
				CAL	3.92±1.06	3.52±0.96		
				FMPS	49.60±21.20	24.80±17.30		
				BOP	43.40±27.30	19.70±14.30		
		EC (n=20)	full mouth	PPD	3.53±0.95	3.15±0.74		
				CAL	4.42±1.43	4.31±1.46		
				FMPS	48.60±21.40	27.90±24.0		
				BOP	33.70±26.10	26.10±19.70		
		NS (n=120)	full mouth	PPD	3.21±0.78	2.49±0.56		
				CAL	3.72±1.07	3.18±0.98		
				FMPS	46.30±19.10	20.30±12.30		
				BOP	41.20±23.10	20.10±13.80		

**Sheokand et al. (2019) (placebo) [83]**	RCT	CS	15-15 sites with PPD ≥5 mm, CAL ≥3 mm and radiographic evidence of vertical osseous defects 3–6 mm	PI	1.55±0.32	0.55±0.14	6 months	Clinical parameters PPD and CAL showed no difference ($p < 0.05$) at baseline in both the groups. At 3 months the reduction in PPD and CAL gain was maximum with respect to Alendronate non smoker group. Although there was significant PPD reduction and CAL gain at 6 months in both smoker and non smoker ($p < 0.001$), overall, the result achieved was better with non smoker group.
				GI	1.50±0.39	0.58±0.15		
				PPD	7.00±0.93	4.73±0.88		
				CAL	7.60±1.24	5.73±0.88		
		NS	15-15 sites with PPD ≥5 mm, CAL ≥3 mm and radiographic evidence of vertical osseous defects 3–6 mm	PI	1.93±0.54	0.62±0.40		
				GI	1.88±0.51	0.63±0.41		
				PPD	6.93±0.80	3.27±0.27		
				CAL	7.80±1.01	4.13±0.83		
**Toker et al. (2012) [84]**	prospective cohort	CS (n=15)	GCF sample site	PPD	4.80±0.50	2.70±0.70	6 weeks	Mean PPD and CAL values were significantly reduced after periodontal treatment in all periodontitis patients ($p < 0.05$). There were no statistically significant differences in PPD and CAL values between CS and NS 6 weeks after treatment. The PI and GI values of all periodontitis patients 6 weeks after treatment were significantly lower than those at baseline ($p < 0.05$). There were no significant differences in the PI and GI values between CS and NS at 6 weeks ($p > 0.05$). Likewise, there were no significant differences in the percentage of BOP between CS and NS ($p > 0.05$), and the percentage of BOP values showed significant decreases in response to periodontal treatment in all patients ($p < 0.05$).
				CAL	8.20±1.10	7.50±1.30		
				PI	2.00±0.60	0.10±0.30		
				GI	2.00±0.30	0.00±0.20		
				BOP*	100.00±NA	0.00±NA		
		NS (n=15)	GCF sample site	PPD	4.80±0.30	2.80±0.60		
				CAL	8.30±1.00	7.50±0.90		
				PI	1.60±0.60	0.20±0.40		
				GI	1.90±0.50	0.20±0.40		
				BOP*	100.00±NA	0.00±NA		
**Tomasi et al. (2004) (control) [85]**	RCT	CS (n=20)	sites with PPD of ≥5 mm and BOP+, with an average of 22 sites/patient	PPD	5.70±0.35	4.61±NA	3 months	The baseline examination revealed no significant difference in mean PPD between CS and NS. The mean PPD reduction in the control group at 3-month was 1.1mm (SD=0.45) for smokers and 1.5mm (0.67) for non-smokers. The mean CAL gain for smokers and non-smokers amounted to 0.5mm (0.56) and 0.8mm (0.71), respectively, in the control group. Multiple regression analysis revealed that smoking and initial PPD negatively
				FMPS*	23.00±NA	16.00±NA		
				BOP*	100±NA	58.00±NA		

		NS (n=20)	sites with PPD of ≥5 mm and BOP+, with an average of 22 sites/patient	PPD	5.70±0.38	4.22±NA		influenced the treatment outcome in terms of PPD reduction and CAL gain, while the use of doxycycline had a significant positive effect.
				FMPS*	38.00±NA	28.00±NA		
				BOP*	100.00±NA	62.00±NA		
**Türkoglu et al. (2016) [86]**	prospective cohort	CS (n=16)	full mouth (at six sites per tooth excluding third molars)	PPD	3.76±0.51	2.61±0.29	3 months	There were significant improvements in PPD and CAL values of whole mouth and sampling sites at 1 and 3 months compared to baseline in both CS and NS (p < 0.001). Whole mouth PPD values of CS were significantly higher compared to those of NS at 1 and 3 months (p = 0.042, p = 0.005). No significant differences were detected in whole mouth CAL values between CS and NS at 1 and 3 months (p > 0.05). The mean PPD value of sampling sites in CS was not different from NS at 1 and 3 months (p > 0.05), while CAL value of CS was significantly higher than NS at 3 months (p < 0.05). Whole mouth BOP and PI values significantly decreased in CS and NS at 1 and 3 months compared to baseline (p < 0.05). Similar improvements in PI values were also observed in sampling sites of both groups at 1 and 3 months compared to baseline (p < 0.001).
				CAL	4.87±0.58	3.92±0.67		
				PI*	NA	NA		
				BOP	61.00±22.00	7.90±4.10		
			GCF sampling sites (CAL ≥6 mm and PPD ≥5 mm)	GCF	0.44±0.19	0.31±0.22		
		NS (n=15)	full mouth (at six sites per tooth excluding third molars)	PPD	3.53±0.62	2.17±0.28		
				CAL	4.50±0.98	3.56±0.72		
				PI*	NA	NA		
				BOP	88.00±16.00	9.70±5.50		
			GCF sampling sites (CAL ≥6 mm and PPD ≥5 mm)	GCF	0.57±0.27	0.26±0.16		
**Vajawat et al. (2022) (control) [87]**	split mouth RCT	CS (n=12)	split mouth design: sites randomly divided into test and control sites	PPD	7.00±1.67	5.36±1.56	3 months	Within the control sites, there was statistical difference in the CAL scores between CS and NS at baseline, 1 and 3 months, with the CS showing greater CAL gain (P<0.05). Within the CS and NS groups, there was no statistical difference in PPD values at any time interval (P> 0.05). No difference in the plaque, gingival, and BOP scores was seen between CS and NS at any time interval (P> 0.05)
				CAL	4.36±1.62	3.82±1.16		
		NS (n=12)		PPD	5.73±1.01	3.55±1.44		
				CAL	2.45±1.03	2.27±0.90		
**Varghese et al. (2020) [88]**	case-control	CS (n=10)	full mouth	PPD	6.40±1.35	5.30±1.70	3 months	All clinical parameters demonstrated reduction in measurements at the 3rd month recall interval. NS displayed significant reduction in GI values compared to CS (P < 0.001). Intergroup comparison within CP groups demonstrated significant reduction in all parameters, except in PPD levels in NS.
				CAL	7.00±1.56	5.50±2.42		
				PI	2.17±0.18	0.72±0.12		
				GI	1.59±0.11	0.69±0.10		
			full mouth	PPD	5.90±0.88	5.60±0.97		

		NS (n=10)		CAL	7.50±2.46	6.10±2.64		
				PI	1.88±0.25	0.67±0.1		
				GI	1.80±0.06	0.33±0.06		
**Vijayakumar et al. (2020) [89]**	comparative interventional	CS (n=26)	full mouth: at six sites of all teeth	PPD	3.11±0.76	2.61±0.65	4 weeks	The periodontal parameters such as PI, GI, recession, PPD, and CAL were reduced after NSPT. CS showed statistically significant lower GI and higher PI, gingival recession, PPD, and CAL as compared to NS. Statistically significant reduction in periodontal parameters was seen in both groups after periodontal therapy. CS showed more periodontal destruction and systemic inflammatory markers compared to NS. After periodontal therapy both groups showed statistically significant improvement in clinical parameters and biochemical parameters excluding IL-8.
				CAL	4.30±1.56	3.56±1.45		
				PI	1.45±0.48	0.39±0.15		
				GI	1.08±0.33	0.44±0.22		
		NS (n=26)	full mouth: at six sites of all teeth	PPD	2.91±0.67	2.40±0.57		
				CAL	3.31±1.03	2.76±0.58		
				PI	1.23±0.42	0.36±0.16		
				GI	1.26±0.42	0.42±0.22		
**Vohra et al. (2020) (SRP) [90]**	RCT	ST (n=32)	full mouth: at six sites of all teeth	PPD	6.50±0.50	5.60±0.40	6 months	Habitual shamma use compromises the outcome of NSPT in patients with CP. In shamma users, at 6-month follow-up, all periodontal parameters were comparable with their respective baseline values. At 3-month follow-up, PI (P< 0.05) and BOP (P < 0.05) were significantly higher among shamma users than controls. There was no significant difference in CAL and PPD in all groups at 3- and 6-month follow-ups. At 6-month follow-up, there was no significant difference in all clinical and radiographic inflammatory parameters among shamma users and controls.
				CAL	4.10±0.30	4.20±0.05		
				FMPS	64.5±5.10	53.4±7.60		
				BOP	71.20±9.30	58.40±8.20		
		NS (n=33)	full mouth: at six sites of all teeth	PPD	5.80±0.60	3.05±0.50		
				CAL	4.30±0.40	4.10±0.06		
				FMPS	52.70±5.10	29.60±4.70		
				BOP	69.30±6.60	31.10±3.40		
**Wan et al. (2009) [91]**	prospective cohort	CS (n=20)	diseased sites (with initial PPD≥5 mm)	FMPS	92.53±10.24	58.27±21.86	12 months	Both NS and CS achieved favourable improvements. This was demonstrated by significant reductions of FMPS, BOP and full-mouth mean PPD in both groups. The proportion of sites with PPD≥5.0mm was significantly reduced after the NSPT in both smokers and non-smokers. However, at 12 months, smokers showed less favourable results in terms of significantly higher percentage residual pockets (PPD≥5.0 mm) than NS.
				BOP	71.89±19.54	42.42±21.54		
				PPD	5.85±0.48	3.00±0.80		
			full mouth (six sites of each tooth,	FMPS	77.36±10.96	33.79±15.07		
				BOP	54.32±13.68	26.91±10.85		

			excluding third molars)	PPD	2.89±0.52	2.01±0.38		
		NS (n=20)	diseased sites (with initial PPD≥5 mm)	FMPS	86.48±14.67	42.45±25.57		
				BOP	90.25±15.86	35.86±22.49		
				PPD	5.94±0.47	2.49±0.50		
			full mouth (six sites of each tooth, excluding third molars)	FMPS	75.45±14.95	26.55±14.19		
				BOP	73.45±21.02	24.92±10.44		
				PPD	2.82±0.77	1.71±0.28		
***Yadav et al. (2024) [92]**	prospective case-control	CS (n=27)	full mouth (six sites of each tooth, excluding third molars)	PPD*	NA	NA	3 months	At baseline, there were no significant differences in clinical parameters between CS and NS periodontitis groups. After 3 months of NSPT, a significant reduction from baseline (P < 0.001) was observed for all the clinical parameters in both CS and NS periodontitis groups.
				CAL*	NA	NA		
				PI*	NA	NA		
				GI*	NA	NA		
				GCF*	NA	NA		
		NS (n=25)	full mouth (six sites of each tooth, excluding third molars)	PPD*	NA	NA		
				CAL*	NA	NA		
				PI*	NA	NA		
				GI*	NA	NA		
				GCF*	NA	NA		
**Yilmaz et al. (2023) [93]**	prospective cohort	CS (n=18)	periodontitis sites	PPD	7.42±0.97	5.11±1.74	12 weeks	While initial clinical measurements did not differ between CS and NS (P > 0.05), at 2, 6, and 12 weeks CS demonstrated higher PD, lower PD reduction (mm, %), fewer sites with PD reduction > 30%, and fewer closed pockets in comparison to non-smokers. PI and BoP scores did not differ between the sites of CS and NS at any time point (P > 0.05).
				CAL	7.64±1.15	5.56±1.78		
				FMPS*	88.90±NA	58.30±NA		
				BOP*	100±NA	38.9±NA		
		NS (n=26)	periodontitis sites	PPD	7.38±0.95	3.69±1.50		
				CAL	7.56±1.04	4.27±1.79		
				FMPS*	84.60±NA	42.30±NA		
				BOP*	100±NA	34.60±NA		
**Zuabi et al. (1999) [94]**	prospective cohort	CS (n=12)	full mouth (six sites of each tooth,	PPD	4.16±NA	3.28±NA	NA	Smokers responded favourably to treatment with significant improvement in most clinical indices. Post-treatment PPD and CAL were similar in CS and NS, while CS had significantly greater post-treatment PI than NS (p=0.013).
				CAL	4.49±NA	3.81±NA		
				PI	0.87±NA	0.34±NA		

			excluding third molars)	GI	1.17±NA	0.61±NA		PPD reduction was greater in CS (p=0.024), while other variables did not show any differences.
		NS (n=14)	full mouth (six sites of each tooth, excluding third molars)	PPD	3.52±NA	3.02±NA		
				CAL	3.87±NA	3.43±NA		
				PI	0.65±NA	0.13±NA		
				GI	1.20±NA	0.50±NA		

Suppl. Table 3. Summary of included studies; CS, cigarette smokers; EC, e-cigarette users; NS, non-smokers; FS, former smokers; Q, quitters; ST, smokeless tobacco users; BOP, bleeding on probing, PI, plaque index, FMPS, full mouth plaque score; GI, gingival index, PPD, probing pocket depth; GCF, gingival crevicular fluid; MBL, marginal bone loss; SRP, scaling and root planning, RSD, root surface debridement; NSPT, nonsurgical periodontal therapy; CP, chronic periodontitis; RCT, randomized controlled trial; OHI, oral hygiene instructions

*Not included in the quantitative synthesis

Data are shown as mean ± SD if available
